# D-lactate and glycerol as potential biomarkers of sorafenib activity in hepatocellular carcinoma

**DOI:** 10.1038/s41392-025-02282-z

**Published:** 2025-06-27

**Authors:** Silvia Pedretti, Francesca Palermo, Miriana Braghin, Gabriele Imperato, Pasquale Tomaiuolo, Meral Celikag, Marta Boccazzi, Veronica Vallelonga, Lorenzo Da Dalt, Giuseppe Danilo Norata, Giorgia Marisi, Ilario Giovanni Rapposelli, Andrea Casadei-Gardini, Serena Ghisletti, Maurizio Crestani, Emma De Fabiani, Nico Mitro

**Affiliations:** 1 DiSFeB, Dipartimento di Scienze Farmacologiche e Biomolecolari “Rodolfo Paoletti”, Università degli Studi di Milano, Milano, Italy; 2 Department of Experimental Oncology, European Institute of Oncology (IEO) IRCCS, Milano, Italy; 3 Biosciences Laboratory, IRCCS Istituto Romagnolo per lo Studio dei Tumori (IRST) “Dino Amadori”, Meldola, Italy; 4 Department of Medical Oncology, IRCCS Istituto Romagnolo per lo Studio dei Tumori (IRST) “Dino Amadori”, Meldola, Italy; 5 Department of Oncology, Vita-Salute San Raffaele University, IRCCS San Raffaele Scientific Institute Hospital, Milano, Italy

**Keywords:** Cancer metabolism, Cancer therapy

## Abstract

Sorafenib, a multi-kinase inhibitor for advanced hepatocellular carcinoma (HCC), often encounters resistance within months of treatment, limiting its long-term efficacy. Despite extensive efforts, reliable plasma biomarkers to monitor drug activity remain elusive. Here, we demonstrate that metabolic reprogramming is a strategic response implemented by cancer cells to survive the therapeutic pressure. Sorafenib suppresses oxidative phosphorylation by disrupting electron transport chain supercomplex assembly and enhancing glycolysis. To mitigate the accumulation of harmful glycolytic byproducts such as advanced glycation end-products (AGEs), sorafenib-treated cells reroute excess dihydroxyacetone phosphate (DHAP) toward glycerol-3-phosphate (G3P) synthesis, supporting glycerolipid metabolism, NAD^+^ regeneration, and redox balance, rather than producing D-lactate via the glyoxalase pathway. Alongside, resistant cells enhance serine metabolism to boost glutathione synthesis, reinforcing antioxidant defenses. Additionally, sorafenib increases reliance on exogenous non-esterified free fatty acids and triglycerides for phospholipid remodeling. The combined effects of glycerolipid remodeling and enhanced antioxidant capacity facilitate ferroptosis escape, diminishing sorafenib’s activity. Leveraging these metabolic insights, we validate our findings by investigating plasma metabolites alteration in HCC patients. We identify D-lactate accumulation as a predictor of treatment response and glycerol accumulation as a marker of resistance, highlighting their potential as novel biomarkers for sorafenib activity. As sorafenib is used in advanced HCC, early detection of treatment response is critical to guiding the therapeutic decision, optimizing treatment strategies, and improving patient outcomes.

## Introduction

Hepatocellular carcinoma (HCC) is the predominant form of primary liver cancer and ranks as the sixth most frequently diagnosed cancer worldwide.^1,2^ It also stands among the leading causes of cancer-related mortality, primarily due to the challenges in early detection and limited treatment efficacy in advanced stages. The development of HCC is closely associated with chronic liver disease, particularly conditions such as chronic hepatitis B and C virus infections, metabolic dysfunction-associated steatotic liver disease (MASLD), alcohol-induced liver damage, and other metabolic disorders.^3^ These underlying liver conditions contribute to a gradual progression toward cirrhosis and eventually malignant transformation. Despite notable advances in diagnostic imaging and biomarker development, HCC often remains asymptomatic in its early stages. As a result, a significant number of cases are diagnosed at an advanced or unresectable stage, leaving few curative treatment options and contributing to a poor prognosis. For patients who are not candidates for surgical resection, liver transplantation, or local ablative therapies, systemic treatments become the cornerstone of clinical management. However, advanced-stage HCC still carries a median survival of less than one year.^3^

Over the past decade, significant progress has been made in the treatment landscape of HCC, particularly with the advent of systemic therapies and their combinations. Alongside immunotherapy,^4^ oral tyrosine kinase inhibitors (TKIs) remain a significant treatment for advanced-stage HCC. Among them, sorafenib, the first systemic agent approved by the U.S. Food and Drug Administration (FDA) for HCC treatment in 2007, has played a foundational role.^5^ Sorafenib exerts its anti-cancer effects by targeting a variety of protein kinases involved in tumor cell proliferation and angiogenesis. Specifically, it inhibits Raf kinase, a key component of the RAF/MEK/ERK signaling pathway, along with vascular endothelial growth factor receptors (VEGFR-1, -2, and -3), platelet-derived growth factor receptor-beta (PDGFR-β), and KIT Proto-Oncogene, Receptor Tyrosine Kinase (c-Kit), among others.^6^ Through this multi-faceted inhibition, sorafenib suppresses tumor growth, vascularization, and metastatic potential, making it a valuable therapeutic agent in HCC management.^7^ Despite its clinical relevance, sorafenib’s therapeutic benefit is often compromised by the rapid emergence of resistance. Many patients initially respond to the drug but subsequently develop resistance, leading to treatment failure and continued disease progression.^8^ This phenomenon underscores the urgent need to identify reliable and predictive biomarkers to monitor treatment efficacy and detect early resistance. However, identifying such biomarkers remains an unmet challenge due to the considerable heterogeneity in HCC biology, encompassing genetic, epigenetic, and metabolic variability. To date, research has primarily focused on genetic mutations^9^ and transcriptomic signatures.^10^ In contrast, the role of metabolism-associated molecules as indicators of therapeutic response remains underexplored.

The emergence of resistance to sorafenib is a complex and multifactorial process influenced by diverse cellular mechanisms, including mitochondrial dysfunction and metabolic reprogramming.^11^ Recent studies have demonstrated that sorafenib affects mitochondrial integrity by altering both organelle morphology and function.^12–16^ These alterations impact mitochondrial bioenergetics and promote shifts in cellular metabolism that can ultimately support tumor survival and growth. However, the specific biochemical pathways by which sorafenib induces these mitochondrial changes and reconfigures cellular metabolism are still not fully understood, posing a significant barrier to overcoming drug resistance. Cancer cells adapt to meet increased energy demands during tumor progression, particularly under therapeutic stress. Dysregulated mitochondrial function and metabolism play crucial roles in activating survival pathways, reducing therapy efficacy, and supporting tumor progression.^17^ Understanding how sorafenib influences these metabolic shifts can help identify potential vulnerabilities in cancer cells, thereby uncovering novel therapeutic targets and strategies to enhance drug efficacy. Additionally, characterizing these changes may also facilitate the discovery of metabolic biomarkers that can predict therapeutic response, ultimately enabling a more personalized approach to HCC treatment.

Here, we show that sorafenib disrupts mitochondrial function by impairing the assembly of electron transport chain (ETC) supercomplexes, thereby forcing cells to shift their metabolism toward glucose as the dominant energy source. The enhancement of glycolysis could lead to the accumulation of harmful byproducts, such as advanced glycation end-products (AGEs). To mitigate this toxicity, cells convert the glycolytic intermediate dihydroxyacetone phosphate (DHAP) into methylglyoxal (MG), which is subsequently detoxified by glyoxalase I (Glo1) and hydroxyacylglutathione hydrolase (Hagh), ultimately producing D-lactate. D-lactate can also contribute to the induction of ferroptosis,^18^ which represents a potential mechanism through which sorafenib may exert its therapeutic effects.^19,20^ In contrast, in sorafenib-resistant cells, metabolic reprogramming redirects excess DHAP toward glycerol-3-phosphate (G3P) synthesis, supporting glycerolipid metabolism, NAD^+^ regeneration, and redox balance rather than producing D-lactate. Additionally, glycerolipid synthesis facilitates membrane phospholipid remodeling, releasing polyunsaturated fatty acids (PUFAs) from the membrane, thereby regulating lipid peroxidation to preserve cellular integrity. Sorafenib compels cells to rely on exogenous free fatty acids and triglycerides for phospholipid remodeling, circumventing lipid peroxidation and enabling evasion of ferroptosis. The excess glycerol, likely resulting from triglyceride breakdown, may cause osmotic imbalances; thus, cells fine-tune its concentration by releasing this molecule into the extracellular space.^21^ These metabolic shifts result in altered plasma levels of D-lactate and glycerol in patients treated with sorafenib, positioning them as potential biomarkers of therapeutic efficacy and resistance in HCC.

## Results

### Sorafenib negatively affects mitochondrial function

The *p53*^−/−^; *Myc* hepatoblast cell line were previously developed and characterized as a model to investigate HCC in vitro.^22^ These cells, marked by p53 loss and Myc overexpression, became immortal in culture and acquired tumorigenic potential, forming liver-derived tumors when injected into nude mice (Supplementary Fig. 1a).^22^ This cell line exhibits a relatively high IC_50_ of 8.5 µM for sorafenib, indicating tolerance to drug treatment similar to other sorafenib-resistant HCC cell models reported in the literature (Supplementary Fig. 1b).^23^

**Fig. 1:**
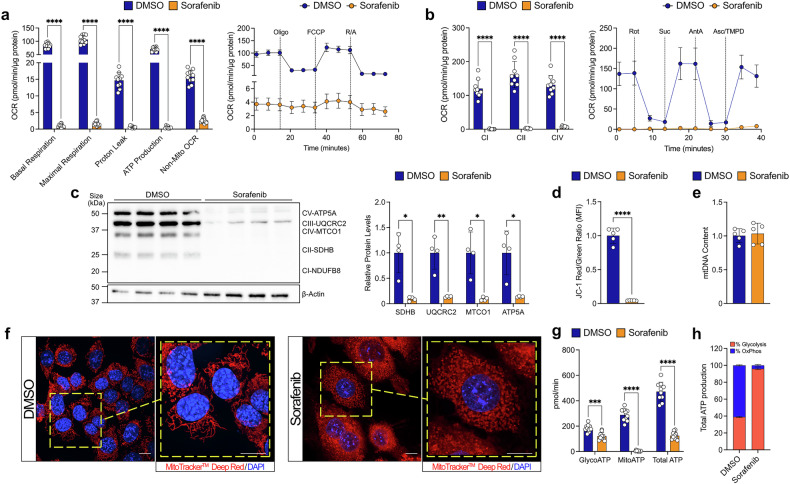
Sorafenib induces mitochondrial dysfunction in *p53*^−/−^; *Myc* hepatoblasts. **a** Seahorse Mitostress Test after 7 days exposure to sorafenib (8.5 µM) or vehicle (DMSO). **b** Mitochondrial respiratory complex activity after 7 days of sorafenib treatment (8.5 µM) or vehicle (DMSO). **c** Levels of several OxPhos subunit proteins in cells treated for 7 days with sorafenib (8.5 µM) or DMSO, with their relative quantification (right). **d** Mitochondrial membrane potential assessed with the JC-1 probe after 7 days of DMSO or sorafenib exposure (8.5 µM). **e** mtDNA quantification after long exposure to sorafenib (7 days, 8.5 µM) compared to control condition (DMSO). **f** Representative morphological analysis of mitochondria using confocal microscopy in cells maintained in control condition (DMSO) or exposed for 7 days to sorafenib treatment (scale bar = 10 µm). In red were shown mitochondria and in blue nuclei. **g**, **h** Real-Time ATP Production Assay showing total ATP production as well as contributions from glycolytic ATP (glycoATP) and mitochondrial ATP (mitoATP), with both absolute quantities (**g**) and percentages relative to total ATP (**h**) after 7 days of DMSO or sorafenib treatment (8.5 µM). For **a,** **g,** **h**, *n* = 10 samples; **b**, *n* = 9 samples; **c**, *n* = 4 samples; **d,** **e**, *n* = 5 samples. All experiments were conducted on *p53*^−/−^; *Myc* hepatoblasts. Data are represented as mean ± SD or as fold change relative to the control condition (DMSO), with p-values determined using multiple t-test corrected for multiple comparisons via the Holm-Sidak method (**a,** **b,** **c,** **g**). For (**d**, **e**), unpaired two-tailed t-test were used. Statistical significance is indicated as follows: * corresponds to *p* < 0.05, ** to *p* < 0.01, *** to *p* < 0.001, and **** to *p* < 0.0001. Oligo oligomycin, FCCP carbonyl cyanide 4-(trifluoromethoxy) phenylhydrazone, R/A rotenone and antimycin A, Rot rotenone, Suc succinate, AntA antimycin A, Asc ascorbate, TMPD N,N,N′,N′- Tetramethyl-p-phenylenediamine

Sorafenib is known to inhibit mitochondrial function.^12–16^ To uncover the metabolic adaptations associated with this effect, we systematically analyzed the impact of drug treatment on mitochondrial function in our *p53*^*−/−*^; *Myc* HCC cell model. A 48-hour treatment with sorafenib significantly reduced basal and maximal respiration, along with ATP production (Supplementary Fig. 1c), and inhibited the activities of ETC complexes I, II, and IV (Supplementary Fig. 1d). Prolonged treatment (7 days) led to an even more pronounced suppression of all the mitochondrial functions (Fig. 1a, b). Additionally, sorafenib treatment lowered levels of several protein subunits involved in oxidative phosphorylation (OxPhos) (Fig. 1c and Supplementary Fig. 1e) and decreased mitochondrial membrane potential at both timepoints (Fig. 1d and Supplementary Fig. 1f). However, mitochondrial DNA (mtDNA) content slightly increased after 48 hours of sorafenib treatment (Supplementary Fig. 1g) and remained unchanged with prolonged treatment (Fig. 1e). Moreover, sorafenib affected also mitochondrial network (Fig. 1f and Supplementary Fig. 1h, 1i) leading to a metabolic shift from OxPhos to glycolysis (Fig. 1g, h, and Supplementary Fig. 1j and 1k). Similar mitochondrial respiratory changes were observed in IR-Huh7, a sorafenib-resistant cell line, compared to its parental (non-resistant) counterpart (Supplementary Fig. 1l).^23^ These shared metabolic features indicate that *p53*^*−/−*^*; Myc* hepatoblasts provide a suitable platform for studying the metabolic mechanism driving drug resistance.

### Sorafenib treatment impairs mitochondrial supercomplex assembly

**Fig. 2:**
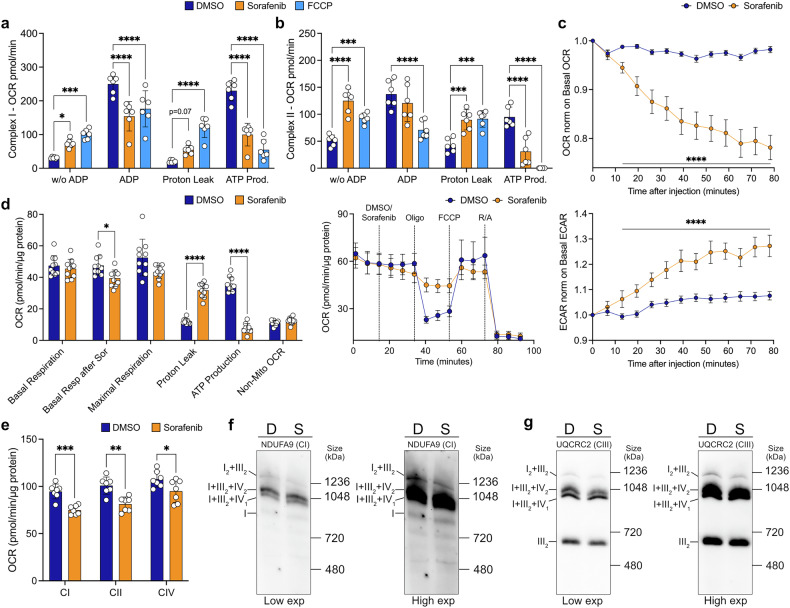
Sorafenib acts as a mitochondrial uncoupler and affects mitochondrial supercomplex assembly. **a** Complex I and (**b**) complex II activities to evaluate uncoupling potential of sorafenib (8.5 µM), FCCP (as positive control, 1 µM) or vehicle (DMSO). **c** OCR (upper) and ECAR (lower) after DMSO or sorafenib injection (8.5 µM), showing the acute metabolic response. **d** Mitostress test with acute injection of DMSO or sorafenib (8.5 µM). **e** Mitochondrial respiratory complexes activities measured after 1 hour exposure to DMSO or sorafenib (8.5 µM). **f** BN-PAGE analysis using the NDUFA9 antibody to detect complex I within mitochondrial supercomplexes in cells treated with DMSO (D) or sorafenib (S) (8.5 µM) for 1 hour. The same membrane was shown with low exposure on the left or high exposure on the right to better visualize signal intensities. **g** BN-PAGE analysis using the UQCRC2 antibody to detect complex III within mitochondrial supercomplexes in cells treated with DMSO (D) or sorafenib (S) (8.5 µM) for 1 hour. The same membrane was shown with low exposure on the left or high exposure on the right to better visualize signal intensities. For (**a,** **b**), n = 6 samples; (**c,** **d**), *n* = 10 samples; (**e**), *n* = 7 samples. All experiments were conducted on *p53*^−/−^; *Myc* hepatoblasts. Data are represented as mean ± SD, with p-values determined using two-way ANOVA with Dunnett’s multiple comparisons test (**a,** **b**), or multiple t-test corrected for multiple comparisons via the Holm-Sidak method (**c**–**e**). Statistical significance is indicated as follows: * corresponds to p < 0.05, ** to *p* < 0.01, *** to *p* < 0.001, and **** to *p* < 0.0001. Oligo oligomycin, FCCP carbonyl cyanide 4-(trifluoromethoxy) phenylhydrazone, R/A rotenone and antimycin A

After thoroughly evaluating the effect of sorafenib on mitochondrial function, we uncovered the specific mechanisms behind sorafenib’s impact on mitochondria. Sorafenib has previously been described as a mitochondrial uncoupler in nonalcoholic steatohepatitis,^16^ and we confirmed this uncoupling activity in our cellular model. Acute treatment of *p53*^*−/−*^; *Myc* hepatoblasts with sorafenib (8.5 µM) immediately before analysis increases the oxygen consumption rate (OCR) driven by complex I substrates (pyruvate and malate) in the absence of ADP. This nucleotide is essential for ATP generation through the ETC, and an increase in OCR in the absence of ADP should indicate mitochondrial uncoupling. Indeed, this effect mirrored that of the well-known uncoupler carbonyl cyanide 4-(trifluoromethoxy) phenylhydrazone (FCCP), indicating significant proton leak (Fig. 2a and Supplementary Fig. 2a). However, ADP-stimulated OCR and ATP production dropped significantly with both sorafenib and FCCP treatments. A similar effect was seen on complex II activity, with sorafenib significantly boosting OCR induced by complex II substrate, succinate, in the absence of ADP while inhibiting ATP production and increasing proton leak (Fig. 2b and Supplementary Fig. 2b). Notably, these effects were not limited to liver cells, as Hek-293 (Supplementary Fig. 2c) and HeLa cells (Supplementary Fig. 2d) exhibited a similar respiratory profile. Altogether, these findings reveal that sorafenib disrupts the crucial coupling between electron transport and ATP synthesis in mitochondria.

Uncouplers like FCCP typically function as protonophores, allowing protons to re-enter the mitochondrial matrix independently of ATP synthase, which ramps up OCR without generating ATP. To test whether sorafenib acts as a protonophore, we acutely treated cells with sorafenib, FCCP, or vehicle in culture medium containing glucose, pyruvate, and glutamine (injection of the drugs was performed at time 0, Fig. 2c and Supplementary Fig. 2e). As expected, FCCP triggered a rapid and sustained rise in OCR (Supplementary Fig. 2e). In contrast, sorafenib treatment led to a progressive OCR decline and a concomitant increase in extracellular acidification rate (ECAR) (Fig. 2c). Further Mitostress assays confirmed that following acute sorafenib treatment, basal respiration, and ATP production decreased, while proton leak increased (Fig. 2d). Strikingly, even a brief 1-hour sorafenib exposure significantly impaired the mitochondrial parameters (Supplementary Fig. 2f) as well as complex I, II, and IV activities (Fig. 2e). Together, these findings indicate that, unlike classical protonophores, sorafenib disrupts mitochondrial function by rapidly impairing OxPhos complex activity.

These results tie directly into the dynamic assembly of ETC complexes into supercomplexes within the inner mitochondrial membrane, which enhances electron transport efficiency while minimizing reactive oxygen species (ROS) production.^24,25^ To investigate whether sorafenib disrupts the structural organization of the mitochondrial respiratory chain, we performed blue-native polyacrylamide gel electrophoresis (BN-PAGE, Supplementary Fig. 3a), followed by immunoblotting for complex I (NADH:ubiquinone oxidoreductase subunit A9, NDUFA9) and complex III (ubiquinol-cytochrome C reductase core protein III, UQCRC2) subunits. Interestingly, 1 hour treatment with sorafenib slightly increased the abundance of free complex I and decreased the I₂ + III₂ band (Fig. 2f). Additionally, we observed a reduction in the UQCRC2 (complex III) levels within mitochondrial supercomplexes (such as I + III₂ + IV_2_ and I₂ + III₂) as well as in its dimeric form, III_2_, as evidenced by the diminished intensity of the corresponding bands (Fig. 2g).

Consistent results were observed using an OxPhos antibody cocktail, which highlighted a decrease in the levels of supercomplexes (I₂ + III₂, I + III₂ + IV_2_, I + III₂ + II_2,_ and III_2_) after 1-hour sorafenib treatment (Supplementary Fig. 3b). Notably, sodium dodecyl sulfate polyacrylamide gel electrophoresis (SDS-PAGE) on isolated mitochondria revealed no differences in the level of different subunits composing ETC complexes (Supplementary Fig. 3c), suggesting that the observed changes are specific to supercomplex assembly rather than a global alteration in mitochondrial protein expression after 1 hour of sorafenib treatment. However, these effects were largely reversible after 24 hours of drug removal in cells treated for 48 hours (Supplementary Fig. 3d), indicating that the impact of sorafenib on mitochondrial function is due to the drug’s presence.

Altogether, these findings indicate that sorafenib impairs mitochondrial supercomplex assembly, likely compromising electron transport chain efficiency and contributing to mitochondrial dysfunction.

### Sorafenib rewires energetic metabolism

Sorafenib disrupts mitochondrial function, but the mechanism underlying its impact on cellular metabolism or the adaptive strategies that enable HCC cells to survive treatment remain poorly understood. To address this open question, we performed mRNA-sequencing on *p53*^*−/−*^*; Myc* hepatoblasts exposed to sorafenib for 48 hours and 7 days. We extended the analysis to include IR-Huh7 and their respective controls, leveraging expression data published by Gao and colleagues.^23^ Across all conditions, metabolic processes (Gene Ontology, GO:0008152) emerged as the most enriched category (Supplementary Fig. 4a). These findings suggest that sorafenib-induced disruption of ETC supercomplexes triggers metabolic adaptation mechanisms that enable cell survival under therapeutic stress.

**Fig. 3:**
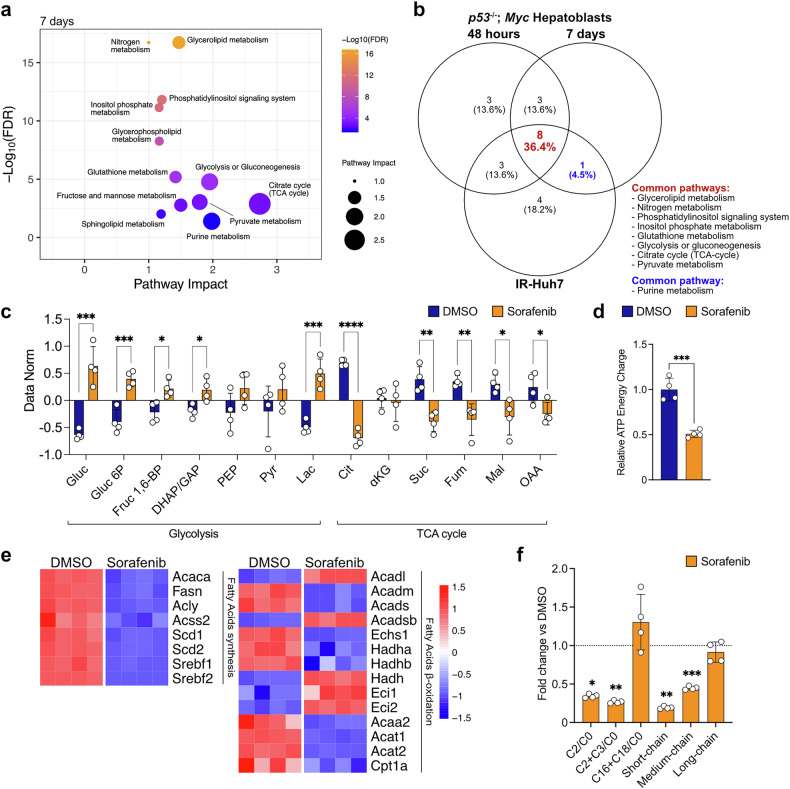
Sorafenib induces metabolic reprogramming toward glycolysis while suppressing fatty acids synthesis and oxidation. **a** Metabolic pathway impact maps using transcriptomics (mRNA-seq) and metabolomics (targeted metabolomics) results after 7 days of sorafenib treatment (8.5 µM) on *p53*^−/−^; *Myc* hepatoblasts, highlighting the most affected metabolic pathway. **b** Venn diagram displaying common affected pathways identified by merged metabolic pathway impact maps across experimental groups (*p53*^−/−^; *Myc* hepatoblasts after 48 hours or 7 days of sorafenib treatment, and IR-Huh7 cell line). **c** Quantification of metabolites associated with glycolysis, gluconeogenesis, the TCA cycle, and pyruvate metabolism following 7 days of DMSO or sorafenib (8.5 µM) treatment. **d** ATP energy charge calculation ([ATP] + 1/2[ADP]/[ATP] + [ADP] + [AMP]) in cells treated with DMSO or sorafenib (8.5 µM) for 7 days. **e** Heatmap of gene expression related to fatty acid synthesis and β-oxidation after 7 days of DMSO/sorafenib (8.5 µM) treatment. **f** Evaluation of acylcarnitine ratios (C2/C0, C2 + C3/C0, C16 + C18/C0) and the sum of carnitines bound to fatty acids of various chain lengths (short, medium and long chains) after 7 days of exposure to DMSO (dotted line) or sorafenib (8.5 µM). For (**a**, **c**, **d**–**f**), *n* = 4 samples. Experiments were conducted on *p53*^−/−^; *Myc* hepatoblasts (**a,** **c,** **d**–**f**). Data are expressed as normalized value (mean ± SD) (**c**) or using fold changes relative to DMSO-treated cells (**d,f**). Only pathways with FDR < 0.05 are showed (**a**). Heatmap analysis showing only genes with FDR < 0.05 (**e**). *p*-values were determined using multiple t-test without multiple comparisons correction (**c**), or corrected via the Holm-Sidak method (**f**). For (**d**), unpaired two-tailed t-test was used. Statistical significance is indicated as follows: * corresponds to *p* < 0.05, ** to *p* < 0.01, *** to *p* < 0.001, and **** to *p* < 0.0001

To deepen our insights, we performed targeted metabolomic analyses and integrated these data with expression profiles. The resulting metabolic pathway impact maps highlight the most affected metabolic pathways (Fig. 3a and Supplementary Fig. 4b, c). Merging the results from all the experimental conditions (*p53*^*−/−*^*; Myc* hepatoblasts exposed to sorafenib for 48 hours or 7 days, and IR-Huh7) revealed a 36% overlap, underscoring the shared metabolic pathways regulated by sorafenib (Fig. 3b). The most prominently shared pathways include glycerolipid metabolism, nitrogen metabolism, phosphatidylinositol signaling system, inositol phosphate metabolism, glutathione metabolism, glycolysis or gluconeogenesis, citrate cycle (tricarboxylic acid (TCA) cycle), and pyruvate metabolism. Notably, purine metabolism was the only common pathway between the 7 days treatment and IR-Huh7, potentially reflecting a long-term metabolic adaptation to sorafenib.

According to the OCR analysis (Fig. 1g, h, and Supplementary Fig. 1j, k), TCA cycle metabolites were decreased, while glycolysis intermediates were generally increased after 7 days of sorafenib treatment (Fig. 3c). These changes resulted in a diminished ATP energy charge (Fig. 3d). Moreover, the expression of genes involved in both fatty acid biosynthesis and β-oxidation was markedly reduced (Fig. 3e). Additionally, indirect indicators of fatty acid β-oxidation, such as the ratio of acetyl-carnitine (C2) to free carnitine (C0), C2 + propionyl-carnitine (C3) to C0, and the total levels of short and medium acyl-carnitines, were reduced after 7 days of sorafenib treatment compared to control condition (Fig. 3f), confirming impaired fatty acid oxidation under sorafenib treatment. In contrast, the levels of long-chain acyl-carnitines and the activity of the mitochondrial fatty acid carrier carnitine palmitoyl transferase I (Cpt1a), assessed by the ratio of palmitoyl-carnitine (C16) + stearoyl-carnitine (C18) to C0, remained unchanged (Fig. 3f). All detected metabolites are shown in Supplementary Table 1.

### Sorafenib-induced D-lactate accumulation is a marker of treatment efficacy

As demonstrated above, sorafenib impairs OxPhos while simultaneously triggering a compensatory increase in glycolysis (Fig. 1g, h and Supplementary Fig. 1j, k), leading to the accumulation of glycolytic intermediates glyceraldehyde-3-phosphate (GAP) and DHAP (Fig. 3c). When glycolysis becomes the primary pathway for energy production, cellular metabolic imbalance arises, promoting the accumulation of toxic byproducts, which contribute to the formation of AGEs. MG is a pivotal player in this process, a highly reactive dicarbonyl compound generated through the non-enzymatic degradation of DHAP and GAP.^26^

**Fig. 4:**
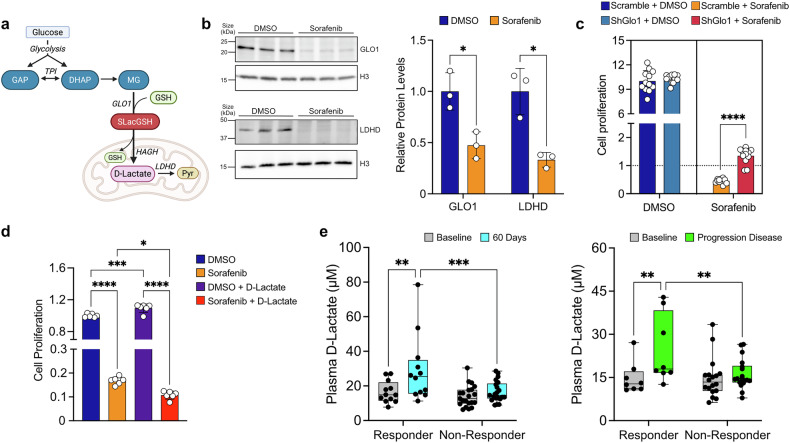
D-lactate as a possible new biomarker for sorafenib efficacy in HCC patients. **a** Graphical representation of D-lactate biosynthesis from glycolytic intermediate through the glyoxalase system, detoxifying MG. TPI triose phosphate isomerase, GAP = glyceraldehyde 3-phosphate, DHAP = dihydroxyacetone phosphate, MG methylglyoxal, GSH = glutathione, SLacGSH = S-Lactoylglutathione, Pyr = pyruvate. Image created with BioRender.com with permission (agreement number: PG289VAMJS; citation to use: https://BioRender.com/vvrxnav). **b** Protein levels of GLO1 and LDHD after 7 days of sorafenib treatment (8.5 µM) compared to control (DMSO), with relative quantification (right). **c** Cell proliferation normalized to the baseline (t0, before starting the treatment) in Scramble and ShGlo1 cells treated for 48 hours with either DMSO or sorafenib (8.5 µM). Proliferation rates were compared between the groups (scramble vs ShGlo1 + DMSO/sorafenib) to evaluate the impact of GLO1 knockdown and drug treatment on cell growth. **d** Cell proliferation following D-lactate treatment (16 mM) in combination with either DMSO or sorafenib (8.5 µM) for 48 hours. Data are expressed as fold change relative to the DMSO + vehicle group. **e** Plasma levels of D-lactate (µM) in HCC patients, comparing responder and non-responder to sorafenib treatment at baseline and after 60 days of administration (left) or at PD (right). For (**b**), *n* = 3 samples; (**c**), *n* = 12 samples; (**d**), *n* = 6 samples; (**e**), 60 days, *n* = 12 Responder, *n* = 21 Non-Responder patients, PD, *n* = 8 Responder, *n* = 18 Non-Responder patients. Experiments were conducted on *p53*^−/−^; *Myc* hepatoblasts (**b**−**d**) or using human plasma derived from HCC patients (**e**). Data are represented as mean ± SD, with p-values determined using unpaired t-test (**c**), multiple t-test corrected for multiple comparisons via the Holm-Sidak method (**b**), Ordinary one-way ANOVA corrected for multiple comparisons using Tukey (**d**) or Two-way Repeated Measures ANOVA (**e**). Statistical significance is indicated as follows: * corresponds to *p* < 0.05, ** to *p* < 0.01, *** to *p* < 0.001, and **** to *p* < 0.0001

Normally, MG detoxification occurs via the glyoxalase system: cytosolic glyoxalase I (GLO1) converts MG into S-lactoylglutathione (SLacGSH), which is transported into the mitochondria and converted by hydroxyacylglutathione hydrolase (HAGH) into D-lactate. Beyond its metabolic role, D-lactate can also promote ferroptosis,^18^ prompting cells to either transport it out of cells or convert it into pyruvate by mitochondrial lactate dehydrogenase D (LDHD) (Fig. 4a). D-lactate is typically present at very low concentration in mammalian cells, especially compared to L-lactate, which increases when OxPhos is disrupted. Similarly, SLacGSH and MG exist at low, transient, and difficult-to-detect levels. Due to the challenges in directly quantifying these metabolites, assessing GLO1 and LDHD protein levels provides an indirect measure of MG detoxification activity.

In our cellular model, both GLO1 and LDHD protein levels decreased after 7 days of sorafenib treatment (Fig. 4b). Indeed, *Glo1* knockdown (Supplementary Fig. 5a), followed by 48 hours of sorafenib treatment, enhanced cell proliferation (Fig. 4c), suggesting that drug-resistant cells avoid MG toxicity and D-lactate production by diverting DHAP/GAP to alternative metabolic routes.

To explore the cytotoxic role of D-lactate, we tested whether adding exogenous D-lactate could enhance sorafenib-induced cell death. Indeed, the combination significantly increased drug efficacy (Fig. 4d). A similar effect was observed with L-lactate (Supplementary Fig. 5b), suggesting that both enantiomers contribute to treatment response. These findings imply that sorafenib-resistant cells minimize intracellular lactate (both D- and L-isoforms) to prevent cell death. Specifically, they efficiently eliminate lactate, as evidenced by the increased ECAR (Fig. 2c) and lactate levels in culture media (Supplementary Fig. 5c), to maintain glycolysis and energy production. At the same time, cells suppress the glyoxalase pathway to prevent D-lactate accumulation. However, while L-lactate remained detectable in the extracellular compartment during sorafenib treatment due to its higher concentration, D-lactate was undetectable in vitro.

These findings suggest that D-lactate levels may be associated with the effectiveness of sorafenib treatment. To test this hypothesis, we measured D-lactate plasma levels in a cohort of HCC patients treated with sorafenib in the INNOVATE study.^27^ Plasma samples were collected at baseline and two-time points: 60 days after starting sorafenib therapy (orally administrated at 400 mg twice daily) or upon the progression of disease (PD). We compared findings between responders (progression-free survival ≥6 months) and non-responders (progression-free survival <6 months) following sorafenib treatment. As expected, L-lactate plasma levels increased in both responder and non-responder groups at both 60 days and PD, reflecting a shift from OxPhos to glycolysis following sorafenib treatment (Supplementary Fig. 5d). However, D-lactate levels were significantly elevated only in responders at both time points, confirming that this harmful metabolite increased in plasma when sorafenib is effective (Fig. 4e). In contrast, non-responders showed no significant changes in D-lactate levels between baseline, 60 days, and PD (Fig. 4e).

Collectively, these findings demonstrate that D-lactate accumulation upon sorafenib treatment is a potential biomarker of the drug’s activity in HCC.

### Glycerol 3-phospahte shuttle represents a metabolic vulnerability to improve sorafenib efficacy

Our previous data indicate that in sorafenib-exposed hepatoblasts, DHAP/GAP are not significantly utilized in the glyoxalase pathway (Fig. 4a–d and Supplementary Fig. 5a-c). Therefore, we investigated alternative metabolic fates for DHAP/GAP, which can be directed toward two pathways: conversion into phosphoenolpyruvate (PEP) to sustain glycolysis or into G3P via glycerol-3-phosphate dehydrogenase 1 (GPD1), a component of glycerophosphate shuttle (G3P shuttle).

**Fig. 5:**
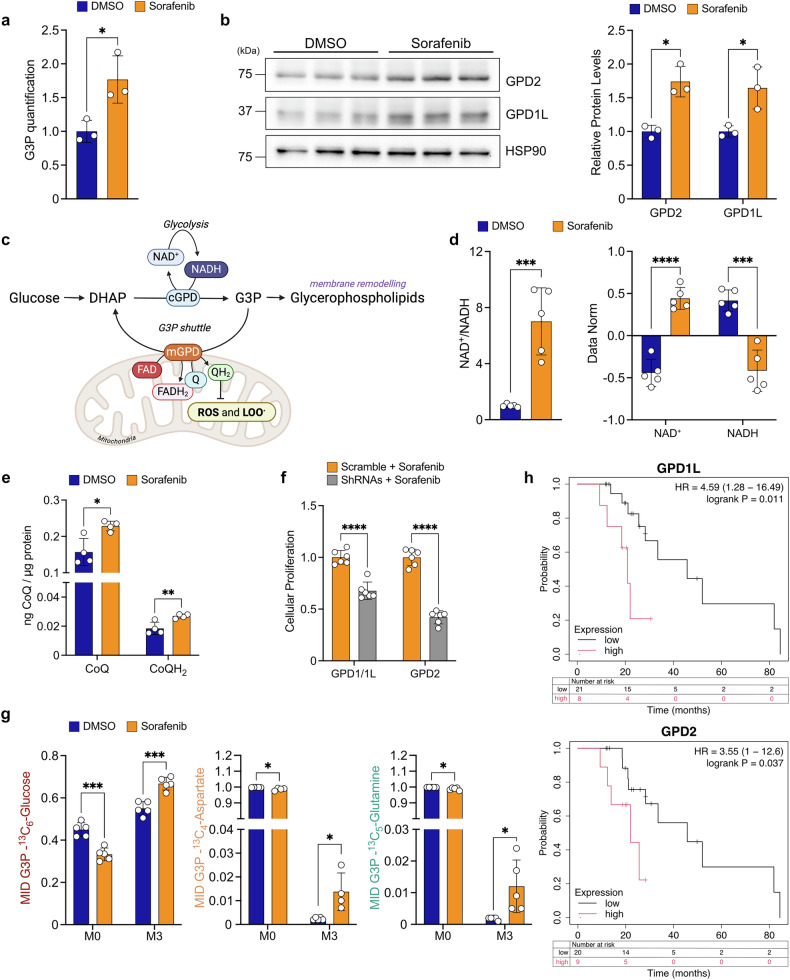
Sorafenib-treated cells rely on glycerol 3-phosphate shuttle to sustain glycolysis and strengthen the defense against oxidative stress. **a** Relative quantification of G3P following treatment with DMSO or sorafenib (8.5 µM) for 48 hours. **b** Protein levels of GPD2 and GPD1L and their quantification (right) after 48 hours of DMSO or sorafenib treatment (8.5 µM). **c** Schematic representation of the G3P shuttle, involving cGPD (cytoplasmic isoform, GPD1/1 L) and mGPD (mitochondrial isoform, GPD2). This process regenerates NAD^+^ and forms FADH_2_, which donates electrons to CoQ (Q), converting it to CoQH_2_ (QH_2_), which acts as an antioxidant against ROS and lipid peroxidation products (LOO^.^). G3P glycerol-3-phosphate, Q ubiquinone, QH_2_ ubiquinol. Image created with BioRender.com with permission (agreement number: PX289LU0TP; citation to use: https://BioRender.com/prnqsrx). **d** NAD^+^/NADH ratio (fold change relative to DMSO) and normalized NAD^+^ and NADH levels after 48 hours of DMSO or sorafenib treatment (8.5 µM). **e** Quantification of CoQ and CoQH_2_ levels after 48 hours of DMSO or sorafenib treatment (8.5 µM). **f** Effect of downregulation of GPD1/1 L and GPD2 on cell proliferation following sorafenib treatment (8.5 µM) for 48 hours. **g** Mass isotopomer distribution (MID) of G3P (M0 and M3) derived from [U-^13^C_6_]-glucose, [U-^13^C_4_]-aspartate, and [U-^13^C_5_]-glutamine, with 6-hour labeling for glucose and 24-hour labeling for amino acids, after 48 hours treatment with DMSO or sorafenib (8.5 µM). **h** Kaplan-Meier survival plot for GPD1L and GPD2. Data from the Kaplan-Meier Plotter database (http://kmplot.com/analysis/), where 29 liver cancer patients treated with sorafenib were divided into high-expression and low-expression groups based on the median expression of these genes, with prognosis analyzed using Kaplan-Meier survival analysis. For (**a,** **b**), *n* = 3 samples; (**d,** **g**), *n* = 5 samples; **e**, *n* = 4 samples; (**f**), *n* = 6 samples. Experiments were conducted on *p53*^−/−^; *Myc* hepatoblasts (**a,** **b,** **d**–**g**). Data are presented as mean ± SD. Statistical analyses were performed using unpaired t-test (**a,** **d** left panel), multiple unpaired t-test (**e,** **g**), with Holm-Sidak correction (**b,** **d,** **f**), or Kaplan-Meier survival analysis (**h**). Statistical significance is indicated as follows: * corresponds to *p* < 0.05, ** to *p* < 0.01, *** to *p* < 0.001, and **** to *p* < 0.0001

Notably, sorafenib-treated cells exhibited elevated G3P levels, suggesting enhanced flux through the G3P shuttle, a crucial system for redox balance and metabolic adaptation (Fig. 5a).

Our cellular model predominantly expressed *Gpd1-like* (*Gpd1l*) over *Gpd1* isoform and glycerol-3-phosphate dehydrogenase 2 (*Gpd2*) (Supplementary Fig. 6a) with both GPD1L and GPD2 proteins significantly upregulated in response to sorafenib treatment (Fig. 5b).

The G3P shuttle plays a vital role in regenerating NAD^+^, which is essential for maintaining glycolysis under conditions of mitochondrial dysfunction (Fig. 5c).^28^ Accordingly, sorafenib-treated cells exhibited elevated NAD^+^/NADH ratio and NAD^+^ levels (Fig. 5d), underscoring the indispensable role of the G3P shuttle in metabolic adaptation.

Beyond NAD^+^ recycling, GPD2 links G3P oxidation to ubiquinone (coenzyme Q, CoQ) reduction, generating ubiquinol (CoQH_2_), a potent antioxidant that mitigates oxidative stress (Fig. 5c).^29^

Notably, sorafenib significantly increased both oxidized and reduced CoQ (Fig. 5e), enhancing its role as a radical-scavenging antioxidant and contributing to the low mitochondrial ROS levels observed in treated cells (Supplementary Fig. 6b).

To validate the pivotal roles of GPD1L in NAD^+^ regeneration and GPD2 in antioxidant defense, we employed a genetic knockdown approach (Supplementary Fig. 6c). ShRNA-mediated silencing of *Gpd1l* or *Gpd2* significantly enhanced the anti-proliferative effects of sorafenib, indicating that disrupting this pathway sensitizes cells to the drug (Fig. 5f).

Isotope labeling experiments using ^13^C_6_-glucose, ^13^C_4_-aspartate, and ^13^C_5_-glutamine revealed that glucose is the primary contributor to G3P synthesis, as indicated by the dominant M3 isotopomer signal derived from ^13^C_6_-glucose (Fig. 5g). However, sorafenib treatment enhanced ^13^C incorporation into G3P from both glucose and amino acids, indicating metabolic flexibility in G3P synthesis (Fig. 5g).

A schematic representation of the metabolic fluxes of glucose, glutamine, and aspartate leading to G3P generation is provided in Supplementary Fig. 6d, illustrating the main changes induced by sorafenib treatment. Essentially, glucose tracing showed a decrease in DHAP/GAP with a concomitant increase in M3 PEP and G3P, indicating that glucose is utilized to fuel glycolysis and G3P synthesis via the G3P shuttle (Supplementary Fig. 6e). Amino acids tracings (glutamine and aspartate) highlight the important function of phosphoenolpyruvate carboxykinase (*Pck*). Notably, RNA-seq analysis revealed that *Pck2*, the mitochondrial isoform, is the only one expressed in these cells, and its expression was further upregulated by sorafenib, indicating its central role in driving M3 PEP production from oxaloacetic acid (OAA) (Supplementary Fig. 6f). These amino acids were converted into OAA and then in PEP to proceed toward G3P synthesis (Supplementary Fig. 6g, h).

Finally, we performed Kaplan-Meier survival analysis using The Cancer Genome Atlas (TCGA) datasets for HCC,^30^ which validated our in vitro findings and revealed a clinical correlation: elevated GPD1L and GPD2 expression in sorafenib-treated patients was associated with reduced overall survival (Fig. 5h).

These findings highlight the relevance of the G3P shuttle in sorafenib resistance, identifying it as a metabolic vulnerability that could be targeted to enhance treatment efficacy and improve patient outcomes.

### Antioxidant adaptation and phospholipid remodeling facilitate ferroptosis escape in sorafenib-treated cells

Sorafenib is well-established as a ferroptosis inducer.^19,20^ Various factors, including p53 dysregulation, may influence cellular tolerance to sorafenib-induced ferroptosis. Typically, p53 inhibits cystine uptake by targeting solute carrier family 7 member 11 (Slc7a11), a key component of the membrane cystine-glutamate antiporter system (xCT), sensitizing cells to ferroptosis. Without p53, this inhibition is lost, potentially allowing cells to resist ferroptotic stimuli better.

To assess sorafenib-induced ferroptosis in *p53*^−/−^; *Myc* cells, we quantified lipid peroxidation, a central biochemical hallmark of ferroptotic cell death. After 48 hours of sorafenib exposure, lipid peroxidation was significantly increased, indicating that sorafenib promotes oxidative stress and lipid peroxidation (Supplementary Fig. 7a). On the contrary, the expression of ferroptosis-related genes was changed, showing a downregulation of pro-ferroptosis genes and an upregulation of ferroptosis-suppressing genes after 48 hours of sorafenib treatment (Supplementary Fig. 7b).

Moreover, upon sorafenib exposure, *p53*^*−/−*^; *Myc* hepatoblasts exhibited increased cysteine levels, likely due to the upregulation of the xCT system, composed of *Slc7a11* and its partner, the solute carrier family 3 member 2 (*Slc3a2*), and/or increased endogenous cysteine synthesis via the transsulfuration pathway (Supplementary Fig. 7b, c). Since cysteine is a precursor of glutathione (GSH), this resulted in significantly elevated GSH levels (Supplementary Fig. 7c). Moreover, protein analysis further confirmed an increase in glutathione peroxidase 4 (GPX4), a key antioxidant enzyme preventing ferroptosis, following sorafenib treatment (Supplementary Fig. 7d). Co-treatment with sorafenib and RSL3, a GPX4 inhibitor, led to complete cell death, confirming that ferroptosis escape contributes to the resistant phenotype (Supplementary Fig. 7e). To exert its antioxidant activity, GSH is oxidized to glutathione disulfide (GSSG) and subsequently regenerated by NADPH-dependent glutathione reductase, maintaining redox balance (Supplementary Fig. 7f). In sorafenib-treated cells, both GSH and GSSG levels were increased; however, the GSH/GSSG ratio was reduced, and the NADP⁺/NADPH ratio was elevated, indicating heightened oxidative stress (Supplementary Fig. 7g, 7h).

One mechanism to sustain NADPH homeostasis and redox balance is through serine metabolism. In our model, sorafenib upregulated genes involved in serine uptake and endogenous synthesis, including phosphoglycerate dehydrogenase (*Phgdh*), as well as enzymes producing NADPH via folate metabolism (Supplementary Fig. 7i). Additionally, serine levels and the purine/pyrimidine ratio were significantly increased (Supplementary Fig. 7j, k). Finally, total and mitochondrial ROS measurements confirmed that sorafenib-resistant cells effectively counteract oxidative stress, underscoring their robust antioxidant adaptations under therapeutic pressure (Supplementary Fig. 6b). All together, these data indicated that sorafenib-treated *p53*^*−/−*^; *Myc* hepatoblasts show an increased antioxidant response.

In addition, we further explored metabolic adaptations that may contribute to ferroptosis escape. Revisiting our metabolic pathway analysis, we identified glycerophospholipid and sphingolipid metabolism as potentially involved pathways (Fig. 3a and Supplementary Fig. 4b, c).

**Fig. 6:**
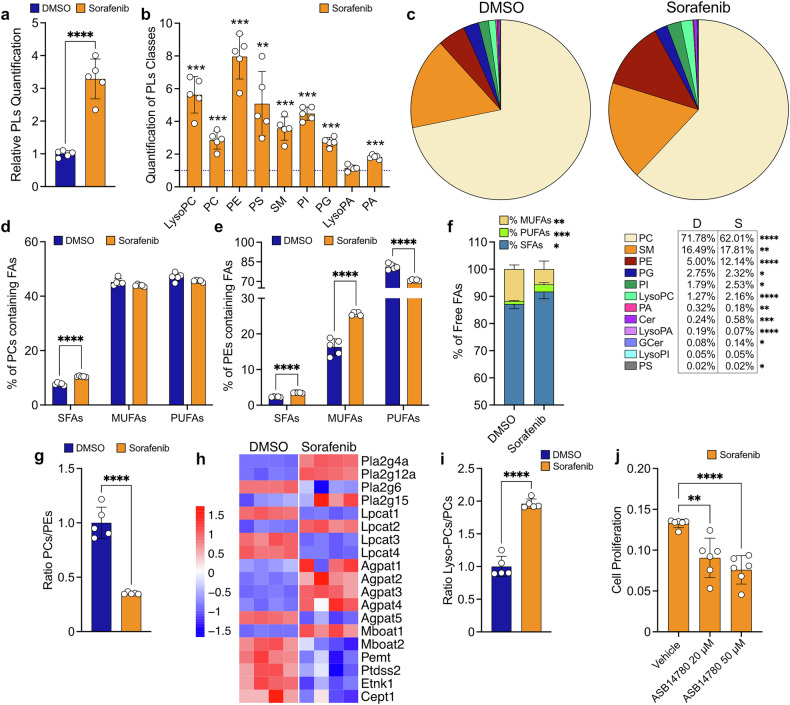
Cells adapt to sorafenib by remodeling membrane phospholipids to counteract ferroptosis. **a** Relative quantification of total phospholipids (PLs) detected in cells treated with DMSO or sorafenib (8.5 µM) for 48 hours. **b** Relative quantification of phospholipids (PLs) categorized by class. The graph shows the fold change relative to control condition (DMSO), with bars representing sorafenib-treated cells (8.5 µM, 48 hours) and a dotted line to 1 corresponding to DMSO-treated cells. **c** Distribution of phospholipids classes (%) in cells treated with DMSO or sorafenib (8.5 µM) for 48 hours, shown as a pie chart with values reported in the table below. **d** Percentage of SFAs, MUFAs, and PUFAs bound in PC after 48 hours of sorafenib treatment (8.5 µM) compared to DMSO. **e** Percentage of SFAs, MUFAs, and PUFAs bound in PE after 48 hours of sorafenib treatment (8.5 µM) compared to DMSO. **f** Percentage of SFAs, MUFAs, and PUFAs detected as free fatty acids inside cells in control (DMSO) and sorafenib-treated conditions (8.5 µM, 48 hours). **g** Ratio of PCs to PEs after 48 hours of DMSO or sorafenib treatment (8.5 µM). **h** Heatmap of gene expression related to genes involved in phospholipids remodeling after 48 hours of DMSO or sorafenib treatment (8.5 µM), showing only genes with FDR < 0.05. **i** Ratio lyso-PCs to PCs after 48 hours of DMSO or sorafenib treatment (8.5 µM). **j** Cell proliferation using ASB14780 (cPLA2α inhibitor, 20 or 50 µM) in combination with either DMSO or sorafenib (8.5 µM) for 48 hours. Data are presented as fold change relative to the corresponding DMSO + ASB14780 (20 or 50 µM) group. Final comparisons were made with the sorafenib vehicle and ASB14780-treated group. For (**a**–**i**), *n* = 5 samples; (**h**), *n* = 4 samples; (**j**) *n* = 6 samples. All the experiments were conducted on *p53*^−/−^; *Myc* hepatoblasts. Data are represented as mean ± SD. Statistical significance was assessed using an unpaired t-test (**a,** **g,** **i**), multiple t-test (**c,** **f**), multiple t-test corrected for multiple comparisons via the Holm-Sidak method (**d,** **e**) the FDR method with a two-stage step-up procedure (Benjamini, Krieger, and Yekutieli) (**b**) or ordinary one-way ANOVA corrected for multiple comparisons using Dunnett’s test (**j**). Statistical significance is indicated as follows: * corresponds to *p* < 0.05, ** to *p* < 0.01, *** to *p* < 0.001, and **** to *p* < 0.0001

Lipid metabolism plays a critical role in ferroptosis regulation, particularly through the control of membrane lipid composition. Ferroptosis is driven by the peroxidation of PUFAs within phospholipids, so the alterations in glycerophospholipid and sphingolipid pathways could contribute to resistance. Additionally, the activation of the glycerophosphate shuttle to maintain redox balance under mitochondrial dysfunction further supports these lipid metabolic adaptations. To gain deeper insight into how sorafenib affects these pathways, we performed a lipidomic analysis to assess changes in the phospholipid composition of *p53*^*−/−*^; *Myc* hepatoblasts (Fig. 6 and Supplementary Fig. 8).

Cells treated with sorafenib for 48 hours exhibited a significant increase in total phospholipid content (pg/µg protein) compared to the control condition (Fig. 6a). This increase was consistent across various phospholipid classes (Fig. 6b), suggesting an upregulation of phospholipid biosynthesis in response to sorafenib treatment.

To delve deeper into lipid class distribution changes, we assessed the relative abundance (percentage, %) of individual phospholipid classes (Fig. 6c). Sorafenib treatment led to a decrease in the percentage of phosphatidylcholines (PCs), phosphatidylglycerols (PGs), phosphatidic acids (PAs), and lyso-phosphatidic acids (lyso-PAs), while increasing the proportion of phosphatidylethanolamines (PEs), phosphatidylinositols (PIs), lyso-phosphatidylcholines (lyso-PCs), ceramides (Cers), glucosylceramides (GCers), and sphingomyelins (SMs) following 48 hours drug treatment (Fig. 6c). Notably, the most abundant classes, PCs and PEs, underwent marked alterations with a decline in the overall percentage of PC species and the concomitant increase of PE species upon sorafenib treatment.

Most importantly, we observed a significant enrichment of PC and PE species containing saturated fatty acids (SFA) (Fig. 6d, e). However, sorafenib treatment led to a specific decrease in PUFA-containing PEs, despite an overall increase in total PE abundance (Fig. 6e). Additionally, intracellular levels of free PUFAs and SFAs (fatty acids not bound to membranes) increased, whereas monounsaturated fatty acids (MUFAs) decreased (Fig. 6f), further supporting the idea that lipid remodeling facilitates ferroptosis escape by limiting peroxidation-prone PUFA incorporation into membranes.

Moreover, sorafenib reduced the PCs/PEs ratio (Fig. 6g), suggesting that cells actively remodel their membranes to adapt to stress, potentially facilitating resistance to the drug.

The transcriptomic analysis supported these lipidomic findings, revealing differential expression of genes involved in phospholipid remodeling (Fig. 6h). Specifically, *Pla2g4a*, the gene encoding cytosolic phospholipase A2 (cPLA2), was one of the top upregulated (Fig. 6h). cPLA2 is responsible for hydrolyzing the *sn*-2 ester bond of membrane phospholipids, releasing the PUFA arachidonic acid and lyso-phospholipids. To further probe the role of PLA2 activity, we evaluated the lyso-PCs/PCs ratio, a marker of PLA2-mediated PCs turnover. The observed increase in the lyso-PCs/PCs ratio (Fig. 6i), along with elevated expression of cPLA2 (Fig. 6h), suggests heightened phospholipase activity, which could be driving phospholipid remodeling and membrane turnover in sorafenib-treated cells. To investigate the contribution of cPLA2 to sorafenib resistance, we inhibited its activity using a specific inhibitor, ASB14780. Co-treatment with sorafenib and ASB14780 at two different concentrations significantly reduced cell proliferation, highlighting the importance of PLA2-mediated phospholipid remodeling in promoting cell survival during drug treatment (Fig. 6j).

The major changes observed, particularly those in PCs and PEs, persisted after 7 days of sorafenib exposure (Supplementary Fig. 8a–g).

Interestingly, the reduced expression of lyso-phosphatidylcholine acyltransferase 3 (*Lpcat3*, Fig. 6h) links phospholipid remodeling to ferroptosis evasion, as *Lpcat3* plays a key role in incorporating PUFAs into phospholipids, rendering them more susceptible to peroxidation. These findings reveal phospholipid remodeling as an ingenious mechanism that cancer cells exploit to evade the ferroptotic effects of sorafenib,^19,20,31^ highlighting potential vulnerabilities for the development of more effective therapies.

### Sorafenib-treated cells rely mainly on extracellular triglycerides

**Fig. 7:**
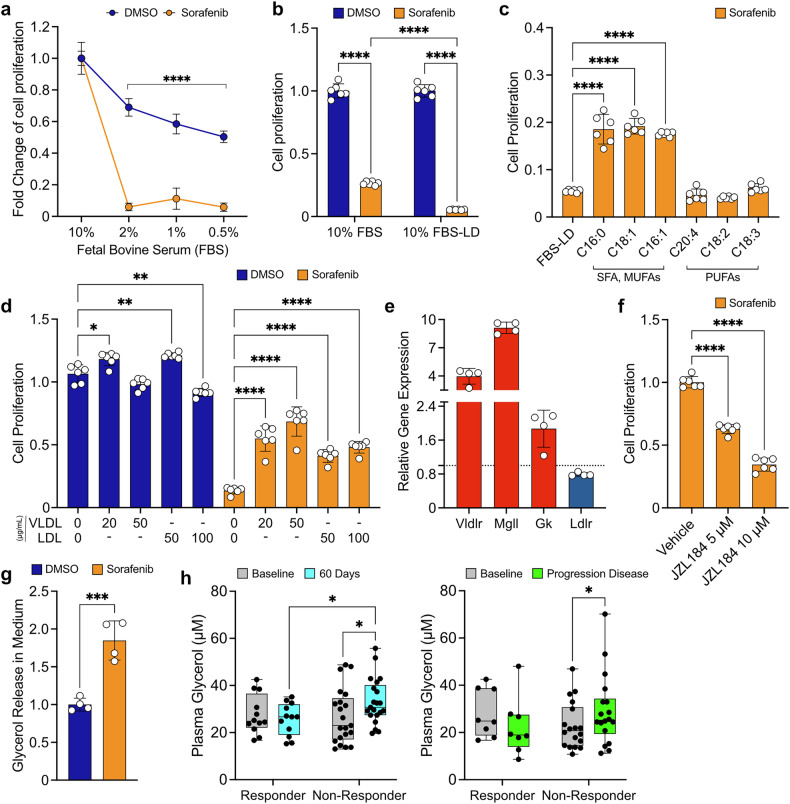
Effects of non-esterified free fatty acids and triglycerides on cell viability in sorafenib-treated cells. **a** Effect of FBS starvation on cell viability during DMSO or sorafenib treatment (8.5 µM) for 48 hours. **b** Impact on the proliferation of cells exposed to DMSO or sorafenib (8.5 µM) for 48 hours in a culture medium containing 10% FBS or FBS-LD. **c** Cell proliferation following the addition of different fatty acids (SFA, MUFAs, and PUFAs; 100 µM) in combination with FBS-LD and sorafenib treatment (8.5 µM) for 48 hours. Data are expressed as fold change relative to DMSO in FBS-LD. **d** Effect of VLDLs or LDLs supplementation in FBS-LD medium on cell proliferation during DMSO or sorafenib treatment (8.5 µM) for 48 hours. The indicated concentrations of VLDLs and LDLs refer to protein quantification associated to the individual lipoproteins. **e** Relative gene expression based on mRNA-seq data (normalized counts and fold change vs. DMSO showed as dotted line to 1) for genes involved in lipoprotein uptake and metabolism (*Vldlr*, *Mgll*, *Gk*, and *Ldlr*). Genes upregulated by sorafenib are shown in red, and downregulated genes are shown in blue. **f** Cell proliferation assay with JZL184 (Mgll inhibitor; 5 or 10 µM) in combination with sorafenib (8.5 µM) for 48 hours. Data are expressed as fold change relative to the sorafenib + vehicle group. **g** Relative glycerol release in extracellular media following 48 hours treatment with DMSO or sorafenib (8.5 µM). **h** Plasma glycerol levels (µM) in HCC patients, comparing responders and non-responders to sorafenib treatment after 60 days of administration (left) or at progression of disease (right). For (**a**–**d,** **f**), *n* = 6 samples; (**e,** **g**), *n* = 4 samples; **h**, 60 days, *n* = 12 Responder, *n* = 21 Non-Responder patients, PD, *n* = 8 Responder, n = 18 Non-Responder patients. Experiments were conducted on *p53*^−/−^; *Myc* hepatoblasts (**a**–**g**) or using human plasma derived from HCC patients (**h**). Data are presented as mean ± SD. Statistical analyses were performed using multiple unpaired t-tests with Holm-Sidak correction (**a**), two-way ANOVA with Tukey’s correction (**b**), ordinary one-way ANOVA with Dunnett’s (**c,** **f**) or Sidak’s correction (**d**), the FDR method with a two-stage step-up procedure (Benjamini, Krieger, and Yekutieli) (**e**), unpaired t-test (**g**), or Two-way Repeated Measures ANOVA (**h**). Statistical significance is indicated as follows: * corresponds to *p* < 0.05, ** to *p* < 0.01, *** to *p* < 0.001, and **** to *p* < 0.0001

Cells require fatty acids to sustain phospholipid synthesis; however, sorafenib-treated cells fail to produce endogenous lipids due to the downregulation of key genes (Fig. 3e) and a significant reduction in citrate, a precursor for fatty acid synthesis (Fig. 3c). One possibility is that they rely on exogenous lipids for membrane turnover and remodeling. To test this dependence, we cultured these cells under fetal bovine serum (FBS) starvation, using decreasing concentrations of FBS, and assessed proliferation. As expected, reducing FBS levels impaired proliferation in DMSO-treated cells (approximately 50% at 0.5% FBS, Fig. 7a). However, the combination of FBS starvation and sorafenib treatment dramatically increased cell death, even at 2% FBS, suggesting that sorafenib-treated cells are heavily reliant on exogenous FBS-derived lipids for survival (Fig. 7a).

To further delineate this dependency, we cultured *p53*^*−/−*^*; Myc* hepatoblasts in medium containing 10% of lipid-depleted (LD) serum (FBS-LD). Lipidomic analysis of FBS-LD revealed significantly reduced triglycerides (TGs) and non-esterified fatty acids (NEFAs), with total cholesterol virtually absent compared to standard FBS (Supplementary Fig. 9a). Interestingly, medium containing 10% FBS-LD significantly enhanced sorafenib’s efficacy compared to that supplemented with 10% FBS, evident as early as 24 hours post-treatment and even more pronounced at 48 hours (Fig. 7b and Supplementary Fig. 9b).

Next, we explored which lipid components increase the resistance to sorafenib. Supplementing FBS-LD with different NEFAs as SFA (palmitic acid, C16:0), MUFAs (oleic acid, C18:1, and palmitoleic acid, C16:1), and PUFAs (arachidonic acid, C20:4; linoleic acid, C18:2 and γ-linolenic acid, C18:3), revealed that SFA and MUFAs significantly improved proliferation in sorafenib-treated cells, whereas PUFAs were ineffective (Fig. 7c). As reported for other cells, among which HepG2,^32^ PUFAs exhibited anti-proliferative effects even in DMSO-treated cells, while SFAs and MUFAs did not promote proliferation in control condition as in sorafenib-treated cells (Supplementary Fig. 9c).

We then tested the other major exogenous lipid components, such as TGs and cholesterol, delivered via very low-density lipoproteins (VLDL) and low-density lipoproteins (LDL). As known, VLDLs are richer in TGs, while LDLs are primarily composed of cholesterol (Supplementary Fig. 9d). Both lipoprotein classes increased proliferation in sorafenib-treated cells cultured in FBS-LD-containing media. Notably, VLDL demonstrated a dose-dependent effect, unlike LDL (Fig. 7d and Supplementary Fig. 9e). This discrepancy likely stems from their triglyceride content: 20 µg/mL of VLDL contains a triglyceride concentration (89 µg/mL) comparable to 100 µg/mL of LDL (triglycerides concentration 64 µg/mL), producing similar proliferation effects (Supplementary Fig. 9d, e). These results highlight triglycerides, rather than cholesterol, as the primary drivers controlling the cellular response to drug treatment.

Consistent with this, cells showed upregulated expression of the VLDL receptor (*Vldlr*), monoglyceride lipase (*Mgll*), and glycerol kinase (*Gk*), while the LDL receptor (*Ldlr*) was downregulated (Fig. 7e). TGs contained within lipoproteins can be hydrolyzed to release free fatty acids (FFAs) and glycerol through the action of Mgll (Supplementary Fig. 9f). Inhibiting Mgll activity using JZL184, a blocker of intracellular free fatty acid and glycerol production, potentiated sorafenib’s efficacy in FBS-containing medium in a dose-dependent manner (Fig. 7f and Supplementary Fig. 9g). On the other hand, JZL184 slightly increased proliferation in DMSO-treated cells (Supplementary Fig. 9h). Taken together, these results indicate that Mgll inhibition selectively enhances sorafenib sensitivity by disrupting triglyceride metabolism.

Furthermore, we observed a significant increase in glycerol release into the culture media of sorafenib-treated cells (Fig. 7g), driven by enhanced intracellular triglyceride breakdown. This finding suggests that glycerol release may represent a potential marker of resistance.

To test this hypothesis, we analyzed plasma glycerol levels in INNOVATE study patients.^27^ Data revealed significantly elevated levels in sorafenib non-responders at 60 days post-treatment and during PD, while responder patients maintained stable glycerol levels (Fig. 7h).

In summary, our findings identify plasma glycerol accumulation as a biomarker of sorafenib resistance in HCC, underscoring the critical role of triglyceride metabolism in this phenotype.

## Discussion

Drug resistance is a significant challenge in oncology, allowing cancer cells to evade therapeutic pressure and drive tumor recurrence. Overcoming this barrier requires early tumor detection, continuous monitoring of adaptive responses, and the identification of novel vulnerabilities for targeted therapies. Identifying reliable biomarkers of treatment efficacy or resistance is essential. Sorafenib, a multi-kinase inhibitor used in the advanced stage of HCC, is often ineffective due to the rise of resistance within a few months after beginning the therapy.^8^ While several strategies have been proposed to counteract this issue,^8^ clinically validated biomarkers for predicting sorafenib efficacy remain limited.

Our study investigates the biochemical mechanisms underlying metabolic adaptations to sorafenib resistance. We identify D-lactate and glycerol as potential biomarkers, reflecting the metabolic rewiring that enables cancer cells to survive sorafenib-induced death. Integrating these markers with existing clinical parameters could improve patient stratification and guide personalized treatments. Sorafenib exerts its effects through multiple mechanisms, including the induction of cell death through ferroptosis,^19,20,33^ an iron-dependent process leading to lipid peroxide accumulation on cellular membranes. Metabolic reprogramming in cancer cells can alter substrate utilization, lipid metabolism, and antioxidant defenses, influencing ferroptosis susceptibility.

Our results show that sorafenib induces mitochondrial dysfunction, disrupting the organization of ETC supercomplexes and shifting metabolism from OxPhos to glycolysis. While this reprogramming supports survival, it could also generate toxic byproducts. The observed accumulation of GAP/DHAP led us to hypothesize that sorafenib-resistant cells may produce more methylglyoxal, a toxic precursor of AGEs,^26^ that is detoxified through the glyoxalase system, leading to D-lactate production. However, our data reveal that the resistant phenotype is characterized by reduced expression of both GLO1 and LDHD, the rate-limiting enzymes of the glyoxalase pathway. Notably, co-treatment with sorafenib and D-lactate enhances the drug’s efficacy. Recently, D-lactate has been proposed to be a ferroptosis inducer.^18^ Based on this, elevated D-lactate levels in the plasma of sorafenib-responsive patients may reflect increased production within tumor cells, leading to ferroptosis-induced cell death and subsequent D-lactate release into circulation, supporting its potential as a therapeutic biomarker of treatment efficacy. Despite strong glycolysis induction in sorafenib-resistant cells, the fate of GAP/DHAP remains unclear. These metabolites can contribute to G3P synthesis, the precursor of glycerophospholipids that control the cellular membrane structure. Additionally, G3P participates in the G3P shuttle, through the action of GPD1L, which sustains glycolysis through the regeneration of NAD^+^ during mitochondrial dysfunction,^28^ and GPD2, which manages redox stress.^29^ Our results show that G3P synthesis is sustained not only by glucose but also by aspartate and glutamine through the action of *Pck2*. This aligns with a prior study indicating that *Pck2* is essential for sorafenib-resistant cells, and its depletion enhances drug efficacy both in vitro and in vivo.^34^ G3P synthesis is closely linked with phospholipid remodeling. Following sorafenib treatment, resistant cells upregulate the expression of *Pla2g4a* (encoding the cytosolic PLA2), which reduces PUFA incorporation into phospholipids and protects membranes from ferroptosis. Supporting this concept, pharmacological inhibition of cytosolic PLA2 enhances sorafenib’s activity. Additionally, sorafenib promotes the incorporation of SFAs into the two major phospholipid classes, PCs and PEs. Notably, the drug increases the levels of PEs, particularly those containing MUFAs, while reducing those with PUFAs. This dual effect alters both the phospholipid and fatty acid composition of the cellular membrane, further reshaping the cell’s lipid profile and enhancing resistance by decreasing vulnerability to ferroptosis.^35^ Previous studies have shown that distinct metabolic adaptations, such as enhanced fatty acids oxidation^36^ or increased fatty acid synthesis,^37^ can influence sorafenib-induced ferroptosis. Given the role of lipid metabolism in shaping phospholipid composition and maintaining membrane integrity, a common feature across these studies and our findings is that, regardless of which lipid metabolic pathway is affected, the PUFA content in membranes must be tightly regulated to prevent ferroptosis. Therefore, targeting distinct lipid metabolic pathways could be a strategy to enhance sorafenib efficacy.

Phospholipids synthesis requires both G3P and fatty acids. However, sorafenib-treated *p53*^*−/−*^*; Myc* hepatoblasts cannot synthesize lipids endogenously and instead depend on exogenous NEFAs and triglycerides. Notably, reducing lipid availability in the culture medium enhances sorafenib sensitivity, whereas supplementation with VLDL or LDL, the primary carriers of triglycerides, confers resistance. Furthermore, blocking triglyceride catabolism through inhibiting MGLL activity reveals an additional metabolic vulnerability, further sensitizing the cells to sorafenib. Interestingly, our data also highlight an increase in glycerol release in the extracellular medium of sorafenib-treated cells, likely originating from triglyceride catabolism or G3P metabolism. While the primary contributor remains unclear, cells actively secrete glycerol to maintain osmotic balance.^21^ This phenomenon highlights glycerol release as a marker of metabolic rewiring and resistance, as observed in the plasma of HCC patients who do not respond to sorafenib treatment. The multifaceted resistance mechanism is further reinforced by the upregulation of antioxidant pathways, including serine metabolism and the transsulfuration pathway, which sustain GSH synthesis. Indeed, the inactivation of *Phgdh*, the rate-limiting enzyme in serine biosynthesis, has been linked to elevated ROS levels, depleted NADPH, and enhanced sensitivity to sorafenib.^38^ Moreover, p53 loss in our cellular model leads to the upregulation of xCT, promoting cystine import and GSH synthesis to shield cells from ferroptotic cell death.^39^

**Fig. 8:**
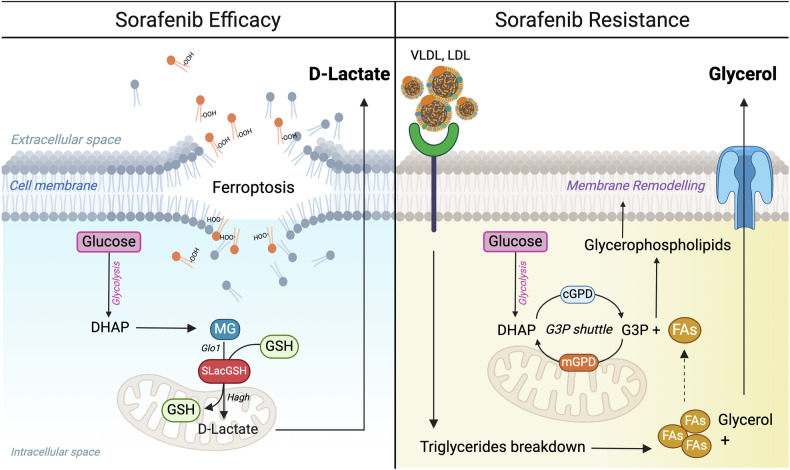
Schematic representation of sorafenib-induced metabolic rewiring in responsive and resistant cancer cells. The figure illustrates the distinct metabolic adaptations in cancer cells in response to sorafenib treatment. On the left side, the mechanism by which sorafenib induces cell death in HCC cells is depicted. Sorafenib triggers mitochondrial dysfunction, leading to increased glycolytic flux and elevated of DHAP production, which is then converted into the toxic metabolite MG. MG is detoxified via the glyoxalase system, involving GLO1 and HAGH, producing D-lactate. The accumulation of D-lactate, together with oxidative stress generated by mitochondrial impairment, promotes ferroptosis by damaging the cellular membrane, ultimately leading to cell death and D-lactate release. On the right side, the mechanism by which cells develop resistance to sorafenib is shown. DHAP is diverted through the G3P shuttle, involving cytosolic GPD1L (cGPD) and mitochondrial GPD2 (mGPD), to form G3P. This metabolite combines with fatty acids (FAs) released from triglyceride (TG) breakdown, which occurs following VLDL receptor-mediated lipid uptake. G3P and FAs support glycerophospholipid synthesis and membrane remodeling, allowing cells to resist ferroptosis. The excess glycerol generated during TG catabolism is exported from the cell. Image created with BioRender.com with permission (agreement number: KH289LLM51; citation to use: https://BioRender.com/oqq1tme)

In summary, sorafenib-resistant cells reprogram their metabolism to cope with the disruption of ETC supercomplex assembly and the resulting dysfunction of OxPhos by enhancing glycolysis. This metabolic shift underpins several adaptive mechanisms: i) inhibiting the production of the D-lactate; ii) generating sufficient G3P to support glycerolipid synthesis^40^ and maintain NAD^+^ levels for sustained glycolysis; iii) utilizing G3P to alleviate oxidative stress via the G3P shuttle and iv) supporting serine synthesis to boost GSH production through the transsulfuration pathway, cystine uptake, and NADPH regeneration.^38,41–43^ Moreover, sorafenib enforces reliance on exogenous NEFAs and/or TGs to support phospholipid remodeling and limit ferroptosis-induced cell death (Fig. 8). Combining sorafenib with RSL3, a GPX4 inhibitor, effectively overcomes resistance by triggering ferroptotic cell death. Our study underscores the importance of understanding the biochemical basis of drug resistance to uncover metabolic vulnerabilities that can be leveraged to improve treatment efficacy and find clinical biomarkers. By analyzing the metabolic response to sorafenib in HCC, we identified serum D-lactate and glycerol as promising indicators of drug response. While further validation in a larger cohort of patients is needed, these findings provide a valuable metabolic framework that may extend beyond HCC to other cancers characterized by metabolic adaptations that promote therapy resistance, thereby supporting personalized treatment strategies and improving therapeutic outcomes.

## Materials and Methods

### Cell lines

All experiments were conducted using an in vitro model of HCC, consisting of mouse-derived hepatoblasts that are knockout for the tumor suppressor *p53* and overexpress the *Myc* oncogene (*p53*^−/−^; *Myc*). This cell line was obtained as described in a previous study^22^ and maintained in high-glucose DMEM growth medium, supplemented with 10% fetal bovine serum (FBS) (cat.no. ECS0165L, Euroclone) and 1% penicillin-streptomycin (cat.no. ECB3001D, Euroclone), at 37 °C, 5% CO_2_ and 90% humidified atmosphere. Cells were treated with sorafenib (8.5 µM) at two different time points: short-term (48 hours) or long-term (7 days).

IR-Huh7, a model of sorafenib-resistant cell line, were obtained from a previous publication.^23^ This cell line was developed through a process of gradually increasing concentrations of sorafenib during culture. Both the parental (sorafenib-sensitive) and IR-Huh7 cells were maintained in high-glucose DMEM growth medium, supplemented with 10% FBS (cat.no. ECS0165L, Euroclone), and 1% penicillin-streptomycin (cat.no. ECB3001D, Euroclone), at 37 °C, 5% CO_2_ and 90% humidified atmosphere. IR-Huh7 cells were cultured in the presence of sorafenib at a concentration of 7μM.

Hek-293 and HeLa cell lines were cultured using high-glucose DMEM growth medium, supplemented with 10% FBS (cat.no. ECS0165L, Euroclone), and 1% penicillin-streptomycin (cat.no. ECB3001D, Euroclone), at 37 °C, 5% CO_2_ and 90% humidified atmosphere. To ensure the reliability of the cell lines used in this study, all were tested for mycoplasma contamination using a PCR detection kit.

### Mice

All procedures involving mice were conducted in accordance with the ethical standards of the Institutional Animal Care and Use Committee of the University of Milan (Italian Ministry of Health permit 1012/2020-PR). Animals were maintained in cages with free access to food and water in a controlled environment with a 12 h/12 h light/dark cycle and a controlled temperature of 22 °C.

Two million *p53*^−/−^; *Myc* hepatoblasts cells were injected subcutaneously into the flask of female CD1-*Foxn1*^*nu*^ outbred immunodeficient mice (6–8 weeks of age, strain code: 086, homozygous, obtained from Charles River Laboratories) to evaluate the capacity of cells to generate tumors in vivo. Following the injection, the animals were monitored continuously. As soon as the tumor became visible (day 18), it was measured every two days using calipers to assess its growth. In accordance with the approved experimental protocol and considering animal welfare, the mice were euthanized when the tumor reached a maximum volume of 2000 mm³ or if ulcerations were developed.

### Patients

INNOVATE (NCT02786342) is a prospective multicenter phase IV study conducted at 10 Italian centers.^27^ The study population consisted of patients with advanced-stage (BCLC C) and intermediate-stage (BCLC B) HCC who were not eligible for loco-regional treatments. The eligibility criteria also included an Eastern Cooperative Oncology Group (ECOG) performance status score ≤2, Child–Pugh class A or B7 with bilirubin <2, adequate hematologic, hepatic and renal function. Patients who had previously received systemic therapy were excluded.

All eligible patients were treated with sorafenib at a dose of 400 mg twice daily. Treatment interruptions and up to two dose reductions (first to 400 mg once daily, and then to 400 mg every 2 days) were permitted to manage drug-related adverse effects.

For each patient, a blood sample was collected at baseline (the same day of sorafenib initiation). Samples were also collected at other timepoints, including 60 days from treatment start (at the same time of first radiological evaluation of disease) and at disease progression (PD).

Treatment continued until the occurrence of radiologic progression, as defined by mRECIST,^44^ symptomatic progression, unacceptable toxicity, or death.

All patients provided written informed consent before the enrollment in the study. The study was approved by ethics committee at each center and complied with the provisions of the Good Clinical Practice guidelines and the Declaration of Helsinki and local laws.

For the purpose of the present study, we analyzed samples from patients in the INNOVATE trial and compared findings between responders and non-responders, defined as patients with progression-free survival upon sorafenib ≥6 months and <6 months, respectively. The cut-off has been selected according to the median progression-free survival previously reported with sorafenib (5.5 months in the phase 3 trial). The baseline patients’ characteristics are reported in Table 1.

**Table 1:** Patients baseline characteristics

Age	median 64 (range 23-84)	
	n	%
**Sex**		
female	7	18.4
male	31	81.6
**ECOG PS**		
0	28	73.7
1	4	10.5
unknown	6	15.8
Aetiology*		
HBV	3	
HCV	17	
alcohol	7	
other/unknown	14	
**BCLC stage**		
B	7	18.4
C	25	65.8
unknown	6	15.8
**AFP**		
<400	17	44.7
>400	12	31.6
unknown	9	23.7
**Portal vein thrombosis**		
yes	20	52.6
no	12	31.6
unknown	6	15.8
**Child-Pugh class**		
A	31	81.6
B	1	2.6
unknown	6	15.8
Previous treatment*		
none	14	
surgery	9	
ablation	14	
TACE	3	
liver transplantation	4	

*Multiple for some patients

*ECOG PS* Eastern Cooperative Oncology Group Performance Status, *HBV* hepatitis B virus, *HCV* hepatitis C virus, *BCLC* Barcelona Clinic Liver Cancer, *AFP* a-fetoprotein, *TACE* transarterial chemoembolization

### Adenoviral transduction for gene downregulation

*Glo1, Gpd2, Gpd1*, and *Gpd1l* were downregulated using adenoviral vectors carrying specific short hairpin RNAs (ShRNAs). Cells were infected with Ad(RGD)-GFP-U6-m-GLO1-shRNA (cat.no. shADV-260185, Vector Biolabs) for *Glo1*, Ad-GFP-U6-m-GPD2-shRNA (cat.no. shADV-279685, Vector Biolabs) for *Gpd2*, and with Ad-GFP-U6-mGPD1L-shRNA-U6-m-GPD1-shRNA (Vector Biolabs) for simultaneous downregulation of *Gpd1* and *Gpd1l*. A control vector containing a scrambled sequence (cat.no. 1122, Vector Biolabs) was used in parallel, using the corresponding multiplicity of infection (MOI). For the downregulation of *Gpd1/1l* and *Gpd2*, cells were infected with 50 MOI, while for *Glo1* with 20 MOI. Infected cells were cultured under standard conditions, and gene knockdown efficiency was confirmed through Western blot.

### Total and Mitochondrial DNA (mtDNA) quantification

Total genomic and mtDNA were isolated using the NucleoSpin DNA kit (cat.no. 740952, Macherey-Nagel). Briefly, cells were lysed in 200 μL of Buffer T1, and then DNA was isolated following the manufacturer’s instructions. Samples were eluted in 100 µl of DNase and RNase-free water and quantified by UV spectrophotometry (NanoDrop 1000 Spectrophotometer, Thermo Fisher Scientific). Mitochondrial DNA content was evaluated by assessing *mt-Nd1* and *36b4* content as mitochondrial and nuclear-encoded genes, respectively. Primers and probes were obtained from Eurofins Genomics MWG-Operon and are available upon request.

### Total RNA isolation, library preparation and mRNA-seq

Total RNA was obtained using a commercial kit (cat.no. 740955.250, Macherey-Nagel). Briefly, cells were washed in ice-cold PBS and lysed in 350 μL of lysis buffer (RA1) supplemented with 15 mM Dithiothreitol (DTT). Total RNA was then isolated following the manufacturer’s instructions and eluted from columns with 60 µl of RNase-free water. The total RNA amount was then quantified by UV spectrophotometry (NanoDrop 1000 Spectrophotometer, Thermo Fisher Scientific). The RNA integrity index (RIN) was evaluated using a TapeStation instrument, and 1000 ng of total RNA were used to prepare the library with the Illumina Stranded mRNA Prep kit (cat.no. 20040532, Illumina) following manufacturer’s protocol. The library was sequenced using IDT for Illumina RNA UD indexes Set A (cat.no. 20091655, Illumina) on Novaseq 6000 sequencer (Illumina).

Quality controls of the FASTQ files was performed using FastQC software, and reads were aligned to the mouse reference genome mm39 (GRCm39) using STAR v2.7.1a.

Filtering was conducted to remove genes with fewer than 10 total read counts across all samples. Differential gene expression analyses between experimental conditions were performed using the R package DESeq2 v1.40.2. Gene Ontology (GO) Biological Process enrichment analysis of DEGs was conducted with WebGestalt (WEB-based Gene SeT AnaLysis Toolkit, https://www.webgestalt.org/#).

### Western blot analyses

#### SDS-Page

Protein quantification analyses were carried out by separating cell lysates on SDS-PAGE. Cells were rinsed in ice-cold phosphate-buffered saline (PBS) and lysed in RIPA buffer (50 mM Tris HCl, pH 8.0, 150 mM NaCl, 1% NP-40, 0,5% sodium deoxycholate, and protease inhibitors). Protein concentration was measured using a BCA Protein Assay Kit (cat.no. EMP014250, Euroclone), and then proteins were loaded on 12 or 15% SDS-PAGE gel. After the electrophoretic run, proteins were transferred to a nitrocellulose membrane using iBlot2 Gel transfer Device and blocked in 0.1% TBS-Tween20 and 5% (w/v) bovine serum albumin (BSA) for 1 hour at room temperature (RT). Membranes were then incubated overnight (O/N) at 4 °C with primary antibodies, previously diluted in 0.1% TBS-Tween20 and 3% BSA. After extensive washes, membranes were incubated with HRP-conjugated secondary antibodies for 1 hour at RT. After washing, membranes were incubated with ECL substrate for band detection.

Primary antibodies were diluted as follows: OXPHOS cocktail 1:2000 (cat.no. ab110413, Abcam), β-actin 1:5000 (cat. no. A5441, Sigma-Aldrich), Histone H3 1:10000 (cat.no. 1B1B2, Cell Signaling or cat.no. ab1791, Abcam for the blot detecting both GPX4 and H3), GLO1 1:1000 (cat.no. ab137098, Abcam), LDHD 1:1000 (car.no. 14398-1-AP, Life technologies), GPX4 1:1000 (cat.no. 52455S, Cell Signaling Technology), GPD2 1:1000 (cat.no. 17291, Proteintech), GDP1L 1:1000 (cat.no. 17263, Proteintech). Blots were quantified by ImageJ software (https://imagej.nih.gov/ij/).

#### Blue Native electrophoresis (BN-Page) - mitochondrial supercomplex assembly

Mitochondria were isolated from cells using mitochondria isolation buffer (sucrose 0.2 M, Tris-HCl pH 7.4 10 mM, EGTA 1 mM, protease and phosphatase inhibitors). Cells were homogenized with 50 strokes using a Dounce homogenizer on ice and then centrifuged at 600 g for 10 min at 4 °C. Mitochondrial proteins were then quantified using the BCA Protein Assay Kit (cat.no. EMP014250, Euroclone), and 50μg of mitochondrial proteins were used for BN-PAGE analysis. A digitonin/protein ratio of 4 g/g was used to solubilize mitochondria. Solubilized mitochondria were incubated on ice for 20 minutes, and then Coomassie G-250 was added (the final concentration is 1/4^th^ of the detergent concentration). Native-PAGE 3-12% gradient gel, Native-PAGE anode buffer, and Dark Blue Native-PAGE cathode Buffer were used to perform the electrophoretic run (150 V for 30 minutes, 250 V for 150 minutes in cold room at 4 °C). After 30 minutes, the Dark Blue Native-PAGE cathode Buffer was changed to the Light Blue Native-PAGE cathode Buffer. Then, the gel was stained with Coomassie staining colloidal solution following the manufacturer’s instructions. Next, proteins were transferred to the PVDF membrane using the iBlot2 Gel transfer Device (20 V for 12 min). The membrane was then fixed with 8% acetic acid, washed with 100% methanol, and incubated with the blocking solution (5% BSA in 0.1% TBS-Tween20) for 1 hour. Primary antibodies were maintained for 90 minutes at room temperature, while the secondary antibodies were kept for 60 min. After washing, membranes were incubated with ECL substrate for band detection.

Primary antibodies were diluted as follows: OXPHOS BN-PAGE cocktail 1:2000 (cat.no. ab110413, Abcam), NDUFA9 1:250 (cat.no. ab14713, Abcam), UQCRC2 1:2000 (cat.no. ab14745, Abcam).

### Targeted metabolomics and tracing analyses

Cells were washed twice in ice-cold PBS and then lysed in 250 µL of ice-cold methanol/acetonitrile/water (v/v, 55/25/20) containing 1 ng/µL of [U-^13^C_6_]-Glucose (cat.no. 389374, Sigma-Aldrich) and [U-^13^C_5_]-Glutamine (cat.no. 605166, Sigma-Aldrich), 0.2 ng/µL of [U-^13^C_2_, ^15^N]-GSH (cat.no. 683620, Sigma Aldrich) as internal standards. Samples were spun at 15,000 g for 15 min at 4 °C, and the supernatant was collected and dried under N_2_ flow at 40 °C. Samples were resuspended in 110 µL of MeOH/H_2_O (v/v, 70:30) for subsequent analysis. For each sample, 10 µL are taken for amino acids analysis. In detail, amino acids quantification was performed through previous derivatization. Briefly, 50 µL of 5% phenyl isothiocyanate (PITC) in 31.5% EtOH and 31.5% pyridine in water were added to 10 µL of each sample. Mixtures were then incubated with PITC solution for 20 min at RT, dried under N_2_ flow, and suspended in 100 µL of 5 mM ammonium acetate in MeOH/H_2_O (v/v, 1:1).

Quantification of different metabolites was performed with a liquid chromatography/tandem mass spectrometry (LC-MS/MS) method using a C18 column (maintained at 50 °C, Biocrates) for amino acids, ZORBAX Stable Bond CN (2.1 × 150 mm, 5 μm; Agilent) for acyl-carnitine and cyano-phase LUNA column (50 × 4.6 mm, 5 μm, maintained at 35 °C; Phenomenex) for metabolites, cofactors and nucleotides, respectively.

Samples were analyzed by different runs in positive (amino acids and acyl-carnitine) and negative (all other metabolites) ion mode (Electrospray ionization, ESI), and a total of 99 analytes were detected. The mobile phases for amino acids and acyl-carnitine analysis were phase A: 0.2% formic acid in water and phase B: 0.2% formic acid in acetonitrile. The gradient was T_0min_ 100%A, T_5.5 min_ 5%A, T_7min_ 100%A with a flow rate of 500 µL/min and 300 µL/min, respectively. The mobile phases for negative ion mode analysis (all other metabolites) were phase A: Water and B: 2 mM ammonium acetate in MeOH. The gradient was 50%B for all the analysis with a flow rate of 600 µL/min. The identity of all metabolites was confirmed using pure standards.

The metabolomics data were acquired using an API-3500 triple quadrupole mass spectrometer (AB Sciex) coupled with an HPLC system. Data analysis and peak review were performed using MultiQuant™ software (version 3.0.2). Raw areas were normalized to the median, followed by log transformation and Pareto scaling. The data processing and analysis were then completed using the MetaboAnalyst 6.0 web tool.^45^

For the metabolic tracing analyses, cells were individually incubated with 5 mM [U-^13^C_6_]-D-Glucose for 6 hours, 1 mM [U-^13^C_5_]-Glutamine (cat.no. 605166, Sigma-Aldrich) or [U-^13^C_4_]-Aspartate (cat.no. 605573, Sigma-Aldrich) for 24 hours before harvesting (U = uniformly labeled metabolite). Following incubation, cells were processed for metabolite extraction (as described above), and the MRM transitions were increased to analyze different isotopomers. Data were expressed as Mass Isotopomer Distribution (MID).

### Phospholipidomics and fatty acids analyses

Cells were washed twice in ice-cold PBS and then lysed in 250 µL of ice-cold methanol/acetonitrile (v/v, 1:1) containing 0.2 ng/µL [U-^13^C_1__6_]-Palmitic acid (cat.no. 389374, Sigma-Aldrich) and [U-^13^C_18_]-Linoleic acid (cat.no. 605166, Sigma-Aldrich) as internal standard. Samples were spun at 15,000 g for 15 min at 4 °C. The supernatant was collected and dried under N_2_ flow at 40 °C. The levels of phospholipids and fatty acids were evaluated by LC-MS/MS analysis. Phospholipids were analyzed in both negative and positive ionization through an API-4000 triple quadrupole mass spectrometer (AB Sciex, Framingham, MA, USA) coupled with an HPLC system (Agilent) and CTC-PAL HTS autosampler (PAL System). For negative ionization analysis, lipids were separated through a cyano-phase LUNA column (50 mm × 4.6 mm, 5 µm; Phenomenex), and the mobile phase was 5 mM ammonium acetate in MeOH, flow rate 500 µL/min. For the positive ionization, analytes were separated on an XTerra RP18 Column (100 × 4.6 mm, 3.5 µm; Waters), and the mobile phase was 0.1% formic acid in MeOH. Free fatty acids were analyzed in negative ion mode on a Hypersil Gold C8 column (100 × 3 mm, 3 μm, maintained at 40 °C; Life Technologies), with these mobile phases A: 10 mM diisopropylethylamine and 15 mM acetic acid in H_2_O/MeOH 97:3 and B: methanol. The gradient was T_0min_ 20%A, T_20min_ 1%A, T_25min_ 1%A, T_25.1 min_ 20%A and T_30min_ 20%A, with a flow rate of 500 µL/min.

Pure standards were used to identify various analytes and generate the quantification curves in fatty acid analysis. Class-specific standards were utilized for phospholipids and a semi-quantitative analysis was performed. The data were then normalized to the total protein content of each sample.

### Transsulfuration pathway analysis

Cells were washed twice in ice-cold PBS and then lysed in 500 µL of ice-cold extraction solution (90% methanol, 0.03% trifluoroacetic acid, 1.3 mM DTT) and incubated for 10 minutes at room temperature. Samples were spun at 16,000 g for 10 min at 20 °C, and the supernatant was collected and dried under N_2_ flow at 22 °C. Samples were reconstituted with 100 µL of ACN/H_2_O (v/v, 90:10), 0.5% formic acid, and 1 mM ammonium formate. The levels of metabolites involved in the transsulfuration pathway were evaluated by LC-MS/MS analysis using an API3500 triple quadrupole mass spectrometer (AB Sciex, Framingham, MA, USA) coupled with an HPLC system ExionLC AC (AB Sciex, USA). The run was performed in ESI positive ion mode, and the mobile phases were A: water, 0.5% formic acid, 1 mM ammonium formate, and B: 90:10 ACN/Water (v/v), 0.5% formic acid, 1 mM ammonium formate. The column used was: Raptor Polar X, 100 mm×2.1 mm ID, 2.7 μm, 90 Å (Restek), with a column guard (Raptor Polar X EXP guard column cartridge 5 mm, 2.1 mm, 2.7μm (Restek). The oven temperature was set to 35 °C. The flow rate was 300 µL/min with the following gradient: T_0min_ 4%A, T_2min_ 4%A, T_10min_ 70%A, T_10.01 min_ 95%A, T_11min_ 95%A, T_11.01 min_ 4%A, T_13min_ 4%A. Pure standards were used to identify various analytes, and the data was then normalized to the total protein content of each sample.

### D- and L-lactate quantification

D- and L-lactate identification in human plasma was performed following a published protocol.^46^ Briefly, 50 µL of plasma was added to 950 µL of cold organic solvent MeOH/ACN (v/v, 1:1) and incubated at low temperature for a brief period (15 min) to enhance protein precipitation. Then, samples were centrifugated at 15,000 g for 10 min at 4 °C, and the supernatant, containing the extracted D-/L-lactate, was moved into clean tubes. Samples were dried under a gentle stream of N_2_ and reconstituted in a mixture of 5% acetic acid, 10% water, and 85% ACN.

D- and L-lactate were identified through a chiral separation using an Astec CHIROBIOTIC® R Chiral Column, 150 × 2.1 mm I.D., 5 µm (Product No. 13019AST, Supelco) with a mobile phase composed by 15% (v/v) 33.3 mM ammonium acetate in H_2_O and 85% (v/v) acetonitrile and a flow rate of 700 µL/min. The column temperature was set at 4 °C. The run was conducted using a quadrupole-time of flight (QTOF) X500R mass spectrometer (ABSciex) coupled to an Agilent 1290 Infinity II UHPLC system (Agilent). The analysis was performed in negative ESI mode, using the same MRM transition for both analytes. Pure standards were used to identify and quantify metabolite amounts in each sample.

### Untargeted lipidomics on FBS and FBS-LD

Untargeted lipidomics analysis was performed on FBS and FBS Lipid Depleted (FBS-LD). A total of 10 µL of each sample was extracted using 250 µL of an ice-cold solution of MeOH/H_2_O/ACN (v/v, 55:25:20). The mixture was vortexed briefly and spun at 15,000 g for 15 min at 4 °C. The resulting supernatants were collected and transferred into a 96-well plate for the analysis. The untargeted LC-MS analysis was performed using an Agilent 1290 Infinity II UHPLC system (Agilent, Santa Clara, CA, USA) coupled to a quadrupole-time of flight (QToF) X500R mass spectrometer (AB Sciex LLC, Framingham, MA, USA). Chromatographic separation was achieved with a Kinetex F5 column (2.6 µm, 100 Å, 150 × 2.1 mm; Phenomenex). The mobile phases consisted of A: water with 0.1% formic acid and B: acetonitrile with 0.1% formic acid. A gradient elution method was applied at a flow rate of 200 µL/min as follows: T_0min_ 99.8% A, T_2min_ 99.8% A, T_5min_ 98% A, T_11min_ 75% A, T_13min_ 2% A, T_17min_ 2% A, T_18min_ 99.8% A, T_20min_ 99.8%A.

Mass spectrometry data were acquired using an ESI source operated in both positive and negative ionization modes with a SWATH-DIA (data independent acquisition) method. Data processing and lipid identification were performed using MS-DIAL software (version 5.3.240719).^47^

### Extraction and quantification of coenzyme Q (CoQ)

The protocol for CoQ extraction and quantification was adapted from a previously published method.^48^ Briefly, cells in culture were washed and then scraped into ice-cold PBS. The cell suspension (200 μL) was transferred to an extraction solution containing 200 μL acidified methanol (0.1% w/v HCl) and 300 μL hexane, vortexed thoroughly, and centrifuged at 17,000 × g for 5 min at 4 °C. The upper hexane layer, containing CoQ, was separated and dried under N_2_. The dried samples were resuspended in 100 μL methanol containing 2 mM ammonium formate and analyzed using an API3500 triple quadrupole mass spectrometer in positive ion mode. The chromatographic separation was performed at 45 °C using a Kinetex C18 column (2.1 × 50 mm, 2.6 μm; Phenomenex) with an isocratic mobile phase of 2 mM ammonium formate in methanol at a flow rate of 0.350 mL/min over 5 min. Pure standards were used to identify CoQ and CoQH_2_, and the data were normalized to the total protein content of each sample.

All mass spectrometry raw data were analyzed using MultiQuant™ software (version 3.0.2) (https://sciex.com/products/software/multiquant-software). Detailed LC-MS methods used for compound quantification are summarized in Supplementary Table 2.

### Glycerol quantification

#### Extracellular media

A glycerol assay kit (cat.no. MAK117, Sigma-Aldrich) was used to quantify glycerol release in extracellular media after sorafenib treatment. The assay was used according to the manufacturer’s instructions. Briefly, 100 μL of the working solution was added to 10 μL of cell supernatant and incubated for 20 min at room temperature. Then, the absorbance was measured at 570 nm. Glycerol concentration was determined using a standard curve, and the total amount of produced glycerol was normalized based on the cell number.

#### Human plasma

20 μL of human plasma were analyzed using a Free Glycerol Assay Kit (cat.no. ab65337, Abcam) following manufacturing instructions. Samples were brought to 50 μL using Assay Buffer, and then 50 μL of reaction mix were added. Then, the solution was incubated at RT for 30 minutes and protected from light. The absorbance was measured at 570 nm, and glycerol was quantified using a standard curve.

### Oxygen consumption rate and extracellular acidification rate assays

All OCRs and ECARs measures were performed using the Seahorse XFe96 Extracellular Flux Analyzer (Seahorse, Agilent) and results were analyzed with Wave Software (Agilent Technologies, Wave for Desktop, version 2.6.1.53).

#### Cell mito stress test

Briefly, the day before the assay, 20,000 cells were seeded into 96-well Seahorse microplates with 80 μL of growth medium. The following day, cells were washed twice with assay media (DMEM supplemented with 10 mM glucose, 1 mM glutamine, 1 mM sodium pyruvate, pH 7.4, cat.no. 103680, Agilent) and incubated for 1 hour in a non-CO_2_ incubator at 37 °C. To evaluate the effect of sorafenib after 1 hour of treatment, the drug was added directly to the assay media just before the incubation began.

The Cell Mito Stress Test was performed following the manufacturer’s instructions, sequentially adding oligomycin (final concentration 1.5 μM, cat.no. O4876, Sigma-Aldrich), FCCP (final concentration 1 μM, cat.no. SML2959, Sigma-Aldrich), and antimycin A/rotenone (final concentration 0.5 μM, cat.no. A8674, cat.no. R8875, Sigma-Aldrich). To assess the acute effect of sorafenib, the vehicle (DMSO) or the drug were added in port A and used at the final concentration of 8.5 μM, followed by the standard injection strategy described above. The parameters of cell respiration were calculated according to the Mito Stress Test protocol. At the end of the assay, cells were lysed using RIPA buffer, and protein levels were measured using BCA kit. Mito Stress data were normalized by the amount of total protein (μg).

#### Complex I, II, and IV activities assay

Cells were plated 2 days before the assay, with 3000 cells per well for DMSO treatment and 6000 cells per well for sorafenib treatment. On the day of the assay, cells were washed twice with MAS solution (mannitol 220 mM, sucrose 70 mM, KH_2_PO_4_ 10 mM, MgCl_2_ 5 mM, HEPES 2 mM, EGTA 1 mM, brought to pH 7.2 using KOH) and then incubated with assay media (MAS solution supplemented with 0.2% BSA-free fatty acids, 4 mM ADP, 10 mM pyruvate, 0.5 mM malate, and 1 nM Plasma Membrane Permeabilizer (Seahorse XF Plasma Membrane Permeabilizer, PMP, cat.no. 102504-100, Agilent)).

To assess mitochondrial complex activities (specifically complex I, II and IV), the injection strategy was as follows: port A: rotenone (final concentration 2 μM, Sigma-Aldrich), port B: succinate (final concentration 10 mM, cat.no. S7501, Sigma-Aldrich), port C: antimycin A (final concentration 4 μM Sigma-Aldrich), and port D: ascorbate/TMPD (final concentration 10 mM/0.2 mM, cat.no A5960, cat.no. T7394, Sigma-Aldrich). Due to the toxicity of PMP to cells, the assay included just 2 repeat measurements after each injection. OCR levels were normalized on total protein content (μg), and the activities of the complexes were calculated using the following OCR values:

- Complex I: OCR basal (Measurement 2) - OCR after rotenone (Measurement 4)
- Complex II: OCR after succinate (Measurement 6)
- Complex IV: OCR after ascorbate/TMPD (Measurement 10)

#### Uncoupling assay

Cells were plated 2 days before the assay, following the protocol of complex activities described above. On the day of the assay, cells were washed twice with MAS solution and then incubated with assay media, which varied depending on the complex analyzed. For complex I, the media consisted of MAS solution supplemented with 0.2% BSA-free fatty acids (cat.no. A3803, Sigma-Aldrich), 1 nM Plasma Membrane Permeabilizer, and either DMSO or sorafenib. For complex II, the media was the same with the addition of 2 μM rotenone. To determine whether sorafenib acts as a mitochondrial uncoupler, ADP and the substrates for each complex were omitted from the assay media. The injection strategy for complex I was: port A: pyruvate and malate (final concentration 5 mM and 2 mM, respectively; cat.no. M0875, Sigma-Aldrich), port B: ADP (final concentration 2.5 mM; cat.no A5285, Sigma-Aldrich), port C: oligomycin (final concentration 1.5 µM), and port D: rotenone (final concentration 0.5 µM). For complex II, the injection strategy was the same as for complex I in ports B and C, while port A contained succinate (final concentration 5 mM), and port D contained antimycin A (final concentration 0.5 µM). For this assay, OCRs were not normalized to protein content, as equal cell numbers were plated and treatments (DMSO, sorafenib, and FCCP) were added just before the measurements began. OCR rates were used to calculate all the different parameters.

- Complex activity w/o ADP: Measurement 4 - Measurement 10
- Complex activity after ADP: Measurement 6 - Measurement 10
- Proton Leak: Minimum rate measurement after Oligo injection - Measurement 10
- ATP production: Last rate measurement before Oligo injection - Minimum rate measurement after Oligo injection

#### Real-time ATP rate assay

Measurements of glycolytic and mitochondrial real-time ATP production were performed using the XF Real-Time ATP Rate Assay Kit (cat.no. 103591–100, Agilent Technologies) following the manufacturer’s instructions. Basal OCR and ECAR were measured at the basal level and after the injection of oligomycin (final concentration 1.5 μM, Sigma-Aldrich) and antimycinA/rotenone (final concentration 0.5 μM, Sigma-Aldrich).

Data were obtained using the Seahorse XF Real-Time ATP Rate Assay Report Generator and normalized to total protein content (µg).

#### Basal respiration and ECAR after drug injection

To assess the effect of sorafenib or FCCP on OCR and ECAR, we used the Seahorse instrument. After three baseline measurements, the instrument injected the compound under investigation. Changes in OCR and ECAR were then monitored for 80 min (13 measurements), and the data obtained were normalized to the baseline values before drug injection.

### JC-1 - mitochondrial membrane potential assay

Cells (1 × 10^6^) were incubated with 2 µM MitoProbe JC-1 (cat.no. M34152, Life technologies) for 15 minutes at 37 °C, 5% CO_2_ in the dark. As a positive control, cells were treated with 35 µM FCCP before the staining. Successively, cells were washed with 0.5 mM EDTA in PBS and resuspended in PBS for the FACS analysis. 20,000 live single cells were analyzed with a cytofluorimeter (FACSCelesta, BD) at different wavelengths: for the DAPI, a laser 405 nm was used, and the emission was collected with a 450/40 nm bandpass filter; for the JC-1, a laser 488 nm has been used and the emission was collected with a 575/26 nm bandpass filter (red) and 530/30 nm bandpass filter (green).

### Mitochondrial and total ROS evaluation

Cells (1 × 10^6^) were incubated with 5 µM MitoSOX Red mitochondrial superoxide indicator (cat.no. M36008, Thermo Fisher Scientific) for the evaluation of the mitochondrial ROS, and with 5 µM CM-H_2_DCFDA for the general oxidative stress quantification (cat.no. C6827, Thermo Fisher Scientific) for 20 min at 37 °C, 5% CO_2_ in the dark. As a positive control, before the staining, cells were treated with 100 µM H_2_O_2_. Successively, cells were washed 3 times with 0.5 mM EDTA in PBS and resuspended in PBS for the FACS analysis. 50,000 live single cells were analyzed with a cytofluorimeter (FACSCelesta, BD) at different wavelengths: for the DAPI, a laser 405 nm was used and the emission was collected with a 450/40 nm bandpass filter; for the ROS evaluation, a laser 488 nm has been used and the emission was collected with a 610/20 nm bandpass filter for the MitoSOX (red) and 530/30 nm bandpass filter for the CM-H_2_DCFDA (green).

### BODIPY^TM^ 581/591 C11 - quantification of lipid peroxidation

Lipid peroxidation was quantified by staining cells with 5 µM BODIPY™ 581/591 C11 (cat.no. D3861, Life technologies). Cells were pretreated with sorafenib/DMSO for 48 hours, followed by the addition of the probe along with the treatment for 1 hour. As positive control cells were treated with 5 µM RSL3 for 1 hour.

After staining, cells were harvested using trypsin and washed twice with PBS. Before analysis, cells were resuspended in ice-cold PBS supplemented with DAPI to exclude dead cells.

Samples were analysed by flow cytometry using MACSQuant Analyzer (Miltenyi Biotec, Bergisch Gladbach, Germany). The fluorescence from DAPI (excitation at 405 nm) and BODIPY^TM^ 581/591 C11 (excitation at 488 nm) was collected with 450/50 nm and 525/50 nm bandpass filters, respectively. Lipid peroxidation was expressed as ratio among treated on untreated cells counted.

Data were acquired and analysed using FlowJo software (version 10.6.1; BD Biosciences, Franklin Lakes, NK, USA).

### Nuclei staining protocol for cellular proliferation assays

Cells were fixed by incubating in 4% paraformaldehyde (PFA) for 10 min at room temperature. Following fixation, cells were washed twice with PBS to remove residual fixative. Next, permeabilization was carried out by incubating the cells in a solution of 0.5% Triton X-100 in PBS for 10 min at room temperature. After permeabilization, cells were washed twice again with PBS.

For nuclear staining, cells were incubated with DAPI at a 1:5,000 dilution in PBS for 30 min at room temperature, protected from light. After the incubation, the DAPI solution was carefully removed, and PBS was added to the samples. Imaging was performed using a Leica THUNDER fluorescent microscope (DMI8, inverted), equipped with a 10X objective (HC PL FluoTAR 10x dry) and LAS X software. For each well, four images were captured to ensure comprehensive coverage. To facilitate nuclei quantification, a custom macro was developed using Fiji software (https://imagej.net/software/fiji/) and StarDist plugin (https://imagej.net/plugins/stardist), enabling automatic counting of nuclei across all samples.

### Mitochondrial morphology and network analysis

Cells were cultured on Nunc glass-based dishes (cat.no. 150682, 27 mm, Thermo Fisher Scientific), and mitochondrial morphology was analyzed at different timepoints (48 hours and 7 days). On the day of the assay, cell culture medium was replaced with staining medium (DMEM medium without phenol red, without FBS, and supplemented by 1% glutamine, 1% glucose, and 1% sodium pyruvate) containing different probes used to stain mitochondria and nuclei. To stain mitochondria, Mitotracker Deep Red FM (500 nM, λ Ex/Em: 644/665 nm) (cat.no. 8778, Cell Signaling) was used. Nuclei were stained by incubating cells with Hoechst 33342 (1:10,000, cat.no. H3570, Invitrogen). Cells were incubated with this solution for 30 min at 37 °C, 5% CO_2_ in the dark. After staining, cells were washed in fresh medium (without probes) and imaged using a Yokogawa Spinning Disk Field Scanning Confocal System (Nikon CSU-W1) equipped with a 100X oil immersion objective.

Image analysis was performed using a custom macro developed in Fiji software (https://imagej.net/software/fiji/), which enabled the automatic quantification of various mitochondrial parameters, including branch number, junction number, maximum branch length, and average branch length.

### Cell viability assay for sorafenib IC_50_ determination

Cell viability was measured using MTT assay. Briefly, an MTT solution (5 mg/mL in PBS) was diluted 1:50 and added to adherent cells at the end of each treatment. Cells were incubated for 1 hour at 37 °C to allow formazan crystal formation. The crystals were solubilized using a 90:10 (v/v) isopropanol:DMSO solution. Finally, absorbance was measured at 570 nm, with values directly correlating to cell viability.

### Very Low-Density Lipoprotein (VLDL) and Low-Density Lipoprotein (LDL) isolation

Blood samples were collected in EDTA tubes. Serum and plasma were obtained by centrifugation at 3,000 rpm for 12 minutes and immediately supplemented with protease inhibitor (cat.no. 87786, Thermo Fisher Scientific). The density of serum was then adjusted from 1.006 g/cm^3^ to 1.019 g/cm^3^ with a solution of KBr 1.35 g/cm^3^. A volume of 500 µL of the KBr density 1.019 g/cm^3^ was then carefully stratified on the top of the plasma in the centrifuge tube. The tubes were centrifuged for 2 hours at 100,000 rpm at 4 °C in a TLA-100.3 Fixed-Angle Rotor (Beckman Coulter, Brea, CA, USA) with a Beckman Optima TL100 Ultracentrifuge (Beckman Coulter, Brea, CA, USA).

The floating VLDLs were then manually isolated, desalted in a PD-10 Desalting Column, with Sephadex G-25 resin with PBS and filtered in a 0.22 µm filter under a biological hood. The bottom part, remaining from VLDL isolation, was then resuspended and the density was adjusted from 1.019 g/cm^3^ to 1.063 g/cm^3^ with the KBr solution at 1.35 g/cm^3^. A total of 2.5 mL of plasma was then put in the ultracentrifuge tube, and 500 µL of 1.063 g/cm^3^ density were carefully layered on the top. The tubes were centrifuged for 2 hours and 30 minutes at 100,000 rpm at 4 °C.

Following the centrifugation, the floating LDLs were collected, desalted and filtered as described for the VLDL. Protein concentration was measured using a BCA Protein Assay Kit following the manufacturer’s instruction. Cells were treated with VLDL at the protein concentration of 20 µg/µL or 50 µg/µL, or with LDL at 50 µg/µL or 100 µg/µL, in media containing lipid depleted FBS for 48 hours in combination with sorafenib or DMSO.

### Triglycerides and cholesterol quantification

TGs and cholesterol and were measured in FBS, FBS-LD (cat.no. ECS0198L, Euroclone), and isolated VLDL and LDL using the clinical-grade reagent. Quantification was performed according to the manufacturer’s protocol for cholesterol (cat.no. A11A01634, ABX Pentra, HORIBA Medical, CH200) and triglycerides (cat.no. A11A01640, ABX Pentra, HORIBA Medical, TR210).

### Use of artificial intelligence

ChatGPT (https://openai.com/index/chatgpt/) was used in this manuscript for English language revision.

### Statistics and reproducibility

A value of *P* < 0.05 was considered statistically significant. The sample sizes and statistical tests are detailed in the figure legends. The minimum sample size for representative experiments was n = 3. Data distribution was assumed to be normal but this was not formally tested. Statistical analyses were performed using Student’s t-tests. In cases with multiple t-tests, the Holm-Sidak method was applied to correct for multiple comparisons. For RNA sequencing data and integrated pathway analyses, only results with a false discovery rate (FDR) < 0.05 were considered significant to limit false positives in large datasets. Comparisons among more than two experimental groups were performed using one-way ANOVA followed by Tukey’s post hoc test or Dunnett’s correction for multiple comparisons. Two-way ANOVA with Dunnett’s or Tukey’s multiple comparisons tests was also employed, depending on the experimental design. Ordinary one-way ANOVA with Dunnett’s or Sidak’s corrections, as well as unpaired t-tests, were used for specific comparisons. For patient data involving multiple conditions and timepoints, a two-way repeated-measures ANOVA was conducted, with Fisher’s Least Significant Difference (LSD) test applied for pairwise comparisons.

Unless otherwise specified, statistical analyses were conducted using GraphPad Prism (version 10.3.1) with significance thresholds set at *P* < 0.05.

## Supplementary information

Supplementary material

Uncropped Western blot

Gating strategies for flow cytometry analysis

MS peak areas

## Data Availability

The data presented in this manuscript have been deposited in NCBI’s Gene Expression Omnibus (GEO) and are accessed through the following GEO Series accession numbers: Hepatoblast (*p53*^−/−^; *Myc*): https://www.ncbi.nlm.nih.gov/geo/query/acc.cgi?acc=GSE273892 Huh7 Parental and IR: GSE158458.^20,23,49^ Kaplan Meier survival plots for GPD1L and GPD2 were generated using the online KMplot tool (https://kmplot.com/analysis/) with data sourced from TCGA database.^30^ All the data are available within the paper and its Supplementary Materials.
