# Supplementary material for: D-lactate and glycerol as potential biomarkers of sorafenib activity in hepatocellular carcinoma
Source: Signal Transduct Target Ther. 2025 Jun 27;10:200. doi: 10.1038/s41392-025-02282-z (PMC12202795; doi:10.1038/s41392-025-02282-z)
Supplement: Supplementary file 1 — Supplementary material [file 41392_2025_2282_MOESM1_ESM.docx]

Supplementary Materials for

**D-lactate and glycerol as potential biomarkers of sorafenib activity in hepatocellular carcinoma**

Silvia Pedretti^1,2^, Francesca Palermo^1^, Miriana Braghin^1^, Gabriele Imperato^1^, Pasquale Tomaiuolo^1^, Meral Celikag^1^, Marta Boccazzi^1^, Veronica Vallelonga^2^, Lorenzo Da Dalt^1^, Giuseppe Danilo Norata^1^, Giorgia Marisi^3^, Ilario Giovanni Rapposelli^4^, Andrea Casadei-Gardini^5^, Serena Ghisletti^1,2^, Maurizio Crestani^1^, Emma De Fabiani^1^, and Nico Mitro^1,2*^.

Correspondence to: [nico.mitro@unimi.it](mailto:nico.mitro@unimi.it)

**This PDF file includes:**

Figures. S1 to S9

Tables. S1 to S2

Figure. S1.

**
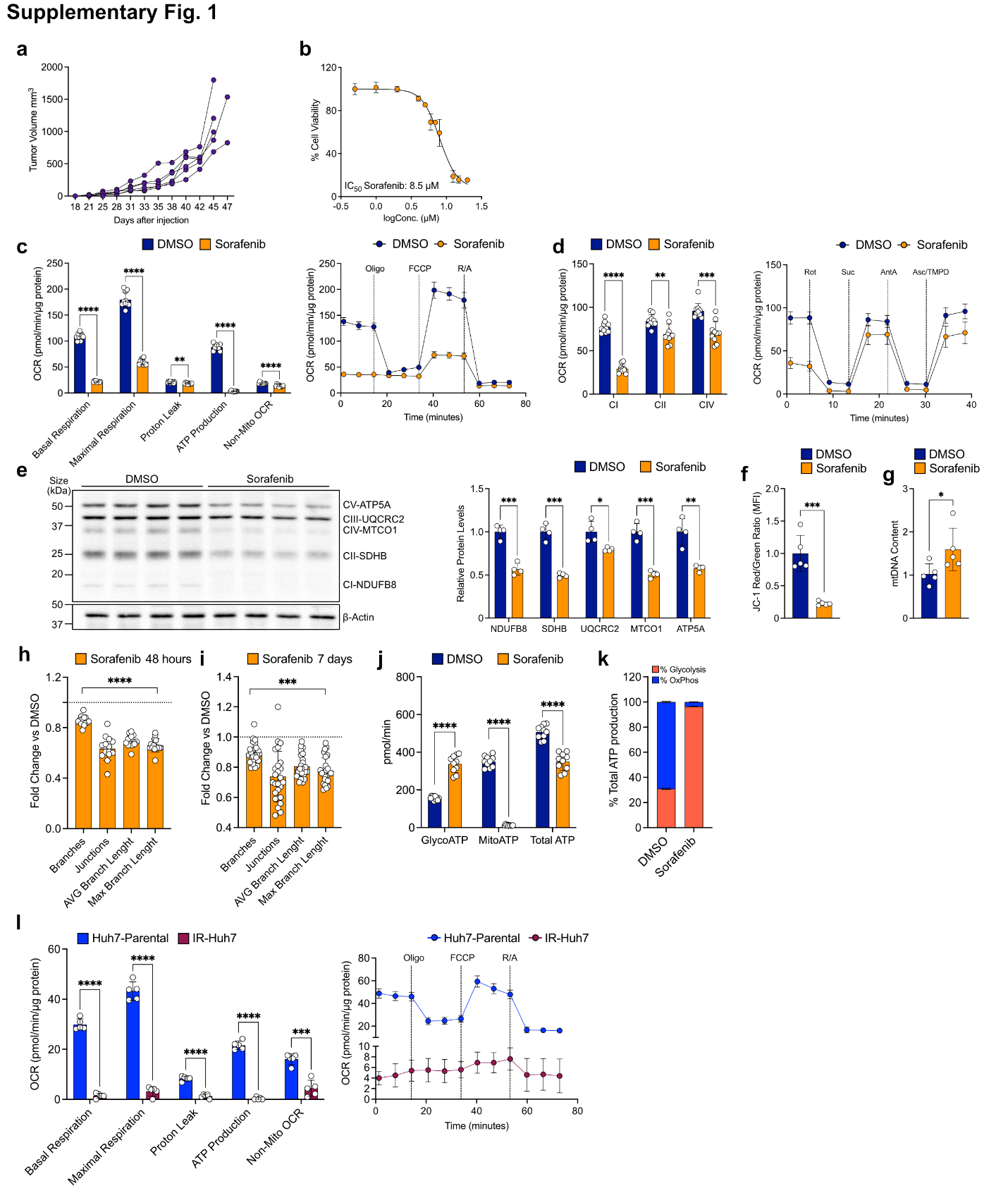
a** Tumor volume measurement following subcutaneous injection of *p53^-/-^*; *Myc* hepatoblasts into mice to assess tumor formation *in vivo*.

**b** IC_50_ determination for sorafenib after 24 hours of treatment, using doses ranging from 0.5 to 20µM.

**c** Seahorse Mitostress Test after short exposure (48 hours) to sorafenib (8.5µM) or vehicle (DMSO).

**d** Mitochondrial respiratory complex activity measured at 48 hours of sorafenib (8.5µM) or DMSO treatment.

**e** Levels of some OxPhos subunit proteins after 48 hours of treatment with sorafenib (8.5µM) or DMSO, with relative quantification (right).

**f** Mitochondrial membrane potential assessed with the JC-1 probe after 48 hours of DMSO or sorafenib (8.5µM) exposure.

**g** Quantification of mtDNA following 48 hours of sorafenib treatment (8.5µM) compared to control condition (DMSO).

**h** and **i** Mitochondrial network analysis after 48 hours (**h**) and 7 days (**i**) of sorafenib treatment (8.5µM), respectively, with DMSO-treated samples used as control. The dotted line represents a value of 1, corresponding to DMSO-treated cells.

**j** and **k** Real-Time ATP Production Assay after 48 hours of DMSO or sorafenib treatment (8.5µM), showing total ATP production contributions from glycolytic ATP (glycoATP) and mitochondrial ATP (mitoATP), with both absolute quantities (**j**) and percentages relative to total ATP (**k**).

**l** Seahorse Mitostress Test on Huh7 parental and IR-Huh7 cells.

For **a**, n=6 samples; **b**, **e**, n=4 samples; **c**, **d**, **j**, **k**, n=10 samples; **f**, **g**, **l**, n=5 samples; **h**, n=15; **i**, n=25, n represents the number of different images analyzed among different experiments. Experiments were conducted on *p53*^-/-^; *Myc* hepatoblasts (**b**, **c**, **d**, **e**, **f**, **g**, **h**, **i**, **j**, **k**), Huh7 parental and IR-Huh7 cells (**l**), or in CD1-*Foxn1^nu^* mice (**a**). Data are represented as mean ± SD or as fold change relative to the control condition (DMSO), with p-values determined using multiple t-test corrected for multiple comparisons via the Holm-Sidak method (**c**, **d**, **e**, **h**, **i**, **j**, **l**). For **f**, **g**, unpaired two-tailed t-test were used. Statistical significance is indicated as follows: * corresponds to p<0.05, ** to p<0.01, *** to p<0.001, and **** to p<0.0001.

Oligo = oligomycin, FCCP = carbonyl cyanide 4-(trifluoromethoxy) phenylhydrazone, R/A = rotenone and antimycin A., Rot = rotenone, Suc= succinate, AntA= antimycin A, Asc = ascorbate, TMPD = N,N,N′,N′-Tetramethyl-p-phenylenediamine.

Figure. S2.

**
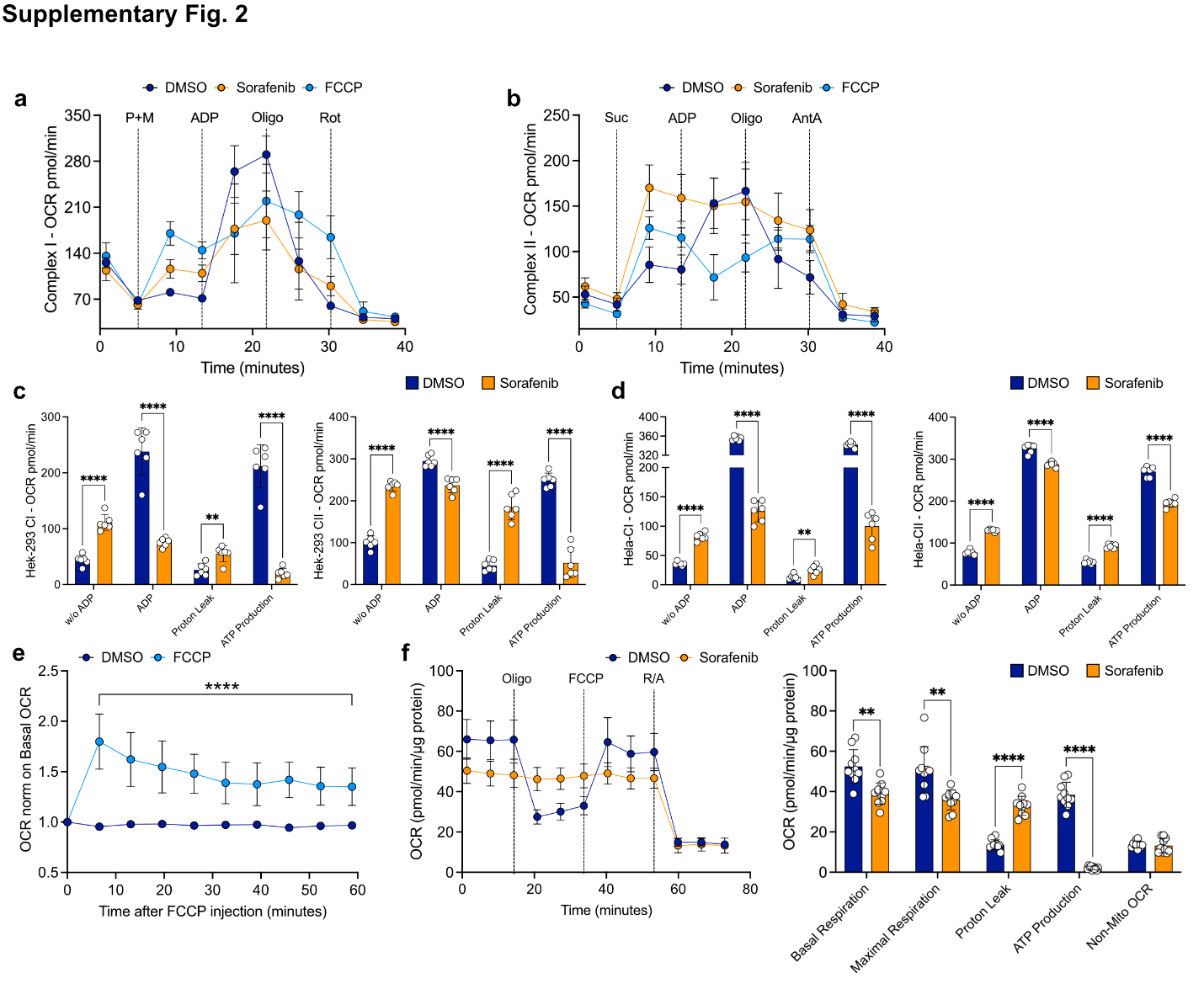
a**, **b** OCR levels relative to the experiment of complex I (**a)** and complex II (**b)** activities (Fig. 2**a**, **b**) to evaluate uncoupling potential of sorafenib (8.5µM). FCCP was used as positive control (1µM) and DMSO as vehicle/control.

**c**, **d** Complex I and complex II activity in Hek-293 (**c**) and in HeLa (**d**) cells to evaluate uncoupling potential of acute treatment with sorafenib (8.5µM) or vehicle (DMSO).

**e** OCR levels after DMSO or FCCP injection (1µM) showing the acute metabolic response.

**f** Mitostress test assay after 1-hour exposure to sorafenib (8.5µM) or vehicle (DMSO).

For **a**, **b**, **c**, **d**, n=6 samples; **e**, **f**, n=10 samples. Experiments were conducted on *p53*^-/-^; *Myc* hepatoblasts (**a**, **b**, **e**, **f**) or on Hek-293 (**c**) and HeLa (**d**) cell lines. Data are represented as mean ± SD, with p-values determined using multiple t-test corrected for multiple comparisons via the Holm-Sidak method. Statistical significance is indicated as follows: ** corresponds to p<0.01, and **** to p<0.0001.

P+M = pyruvate + malate, Suc = succinate, Oligo = oligomycin, Rot = rotenone, AntA = antimycin A, FCCP=carbonyl cyanide 4-(trifluoromethoxy) phenylhydrazone. R/A = rotenone and antimycin A.

Figure. S3.

**
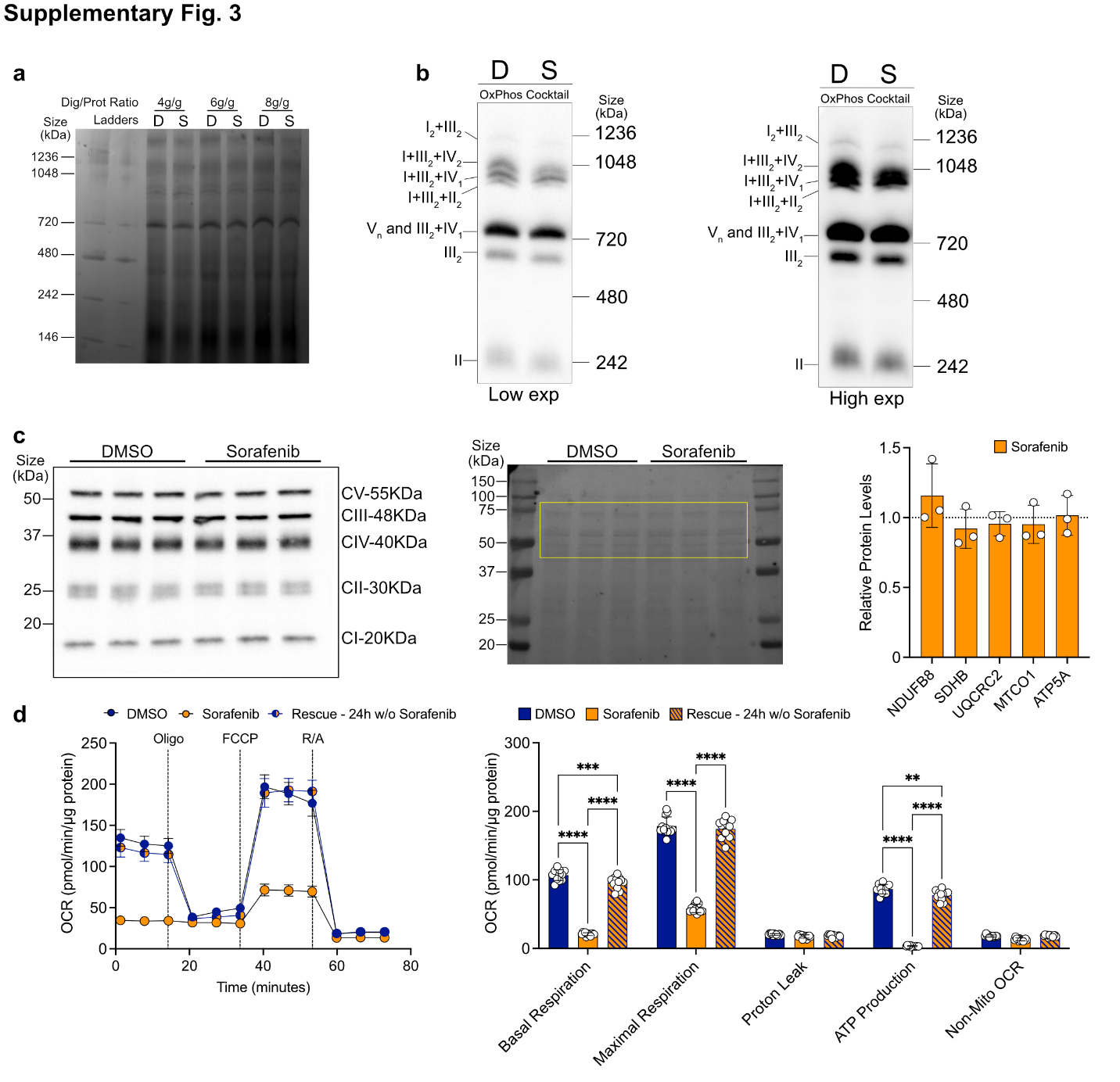
a** Coomassie-stained gel from BN-PAGE analysis to evaluate mitochondrial supercomplex assembly. Different lanes represent mitochondrial lysates from cells under different experimental conditions (D = DMSO, S = sorafenib, 8.5µM). Mitochondria were solubilized using increasing digitonin (Dig)/protein ratios (4, 6, or 8 g/g) to optimize membrane disruption.

**b** BN-PAGE analysis using an OxPhos cocktail antibody to detect mitochondrial supercomplexes in cells treated for 1-hour with DMSO (D) or sorafenib (S) 8.5µM. The same membrane was shown with low exposure on the left or high exposure on the right, to better visualize signal intensities. The membrane was initially probed with an antibody against UQCRC2 (Figure 2g) and then probed with OXPHOS cocktail antibody.

**c** Protein levels of the indicated OxPhos subunits (CV = ATP5A, CIII = UQCRC2, CIV = MTCO1, CII = SDHB, CI = NFUFB8) after 1 hour of treatment with DMSO or sorafenib treatment (8.5µM) (left), with a Ponceau-stained membrane used as the loading control for the Western blot experiment (center), and relative proteins expression quantification (right). The yellow square indicates the portion of the membrane used for the normalization of the Western blot.

**d** Mitostress test in cells treated with DMSO or sorafenib (8.5µM) for 48 hours and in drug washed-out cells, where the drug was removed after 48 hours treatment and cells were allowed to recover for 24 hours before the assay (DMSO and sorafenib treated cells are the same showed in Supplementary Fig. 1c).

For **c**, n=3 samples; **d**, n=12 samples. All experiments were conducted on *p53*^-/-^; *Myc* hepatoblasts. Data are represented as mean ± SD or as fold change relative to the control condition (DMSO), with p-values determined using multiple t-test corrected for multiple comparisons via the Holm-Sidak method (**c**) or two-way ANOVA with Tukey’s multiple comparisons test (**d**). Statistical significance is indicated as follows: ** corresponds to p<0.01, *** to p<0.001, and **** to p<0.0001.

Oligo = oligomycin, FCCP=carbonyl cyanide 4-(trifluoromethoxy) phenylhydrazone, R/A = rotenone and antimycin A.

Figure. S4.

**
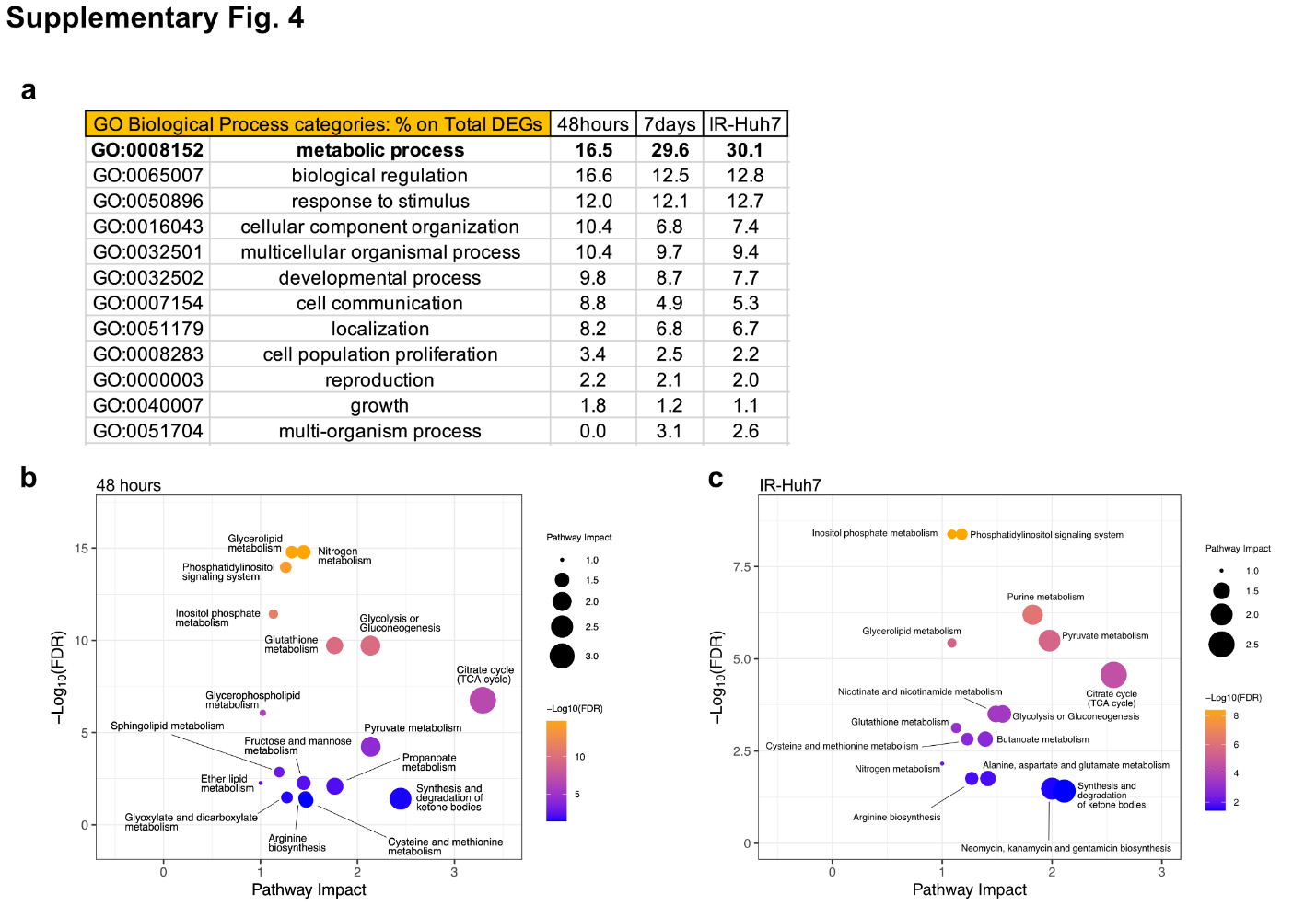
a** Percentage of total DEGs within enriched GO biological process categories, displayed in a table. The first column lists the GO biological process categories, and the following columns show the percentage of DEGs in each category across experimental groups (*p53*^-/-^; *Myc* hepatoblasts after 48 hours or 7 days of sorafenib treatment (8.5µM), as well as for the IR-Huh7 cell line).

**b** and **c** Metabolic pathway impact maps based on transcriptomics (mRNA-seq) and metabolomics (targeted metabolomics) results in *p53*^-/-^; *Myc* hepatoblasts after 48 hours of sorafenib treatment (8.5µM), and IR-Huh7 cell line compared to their relative controls (DMSO or Huh7 parental cells). Only pathways with FDR<0.05 are shown.

Figure. S5.

**
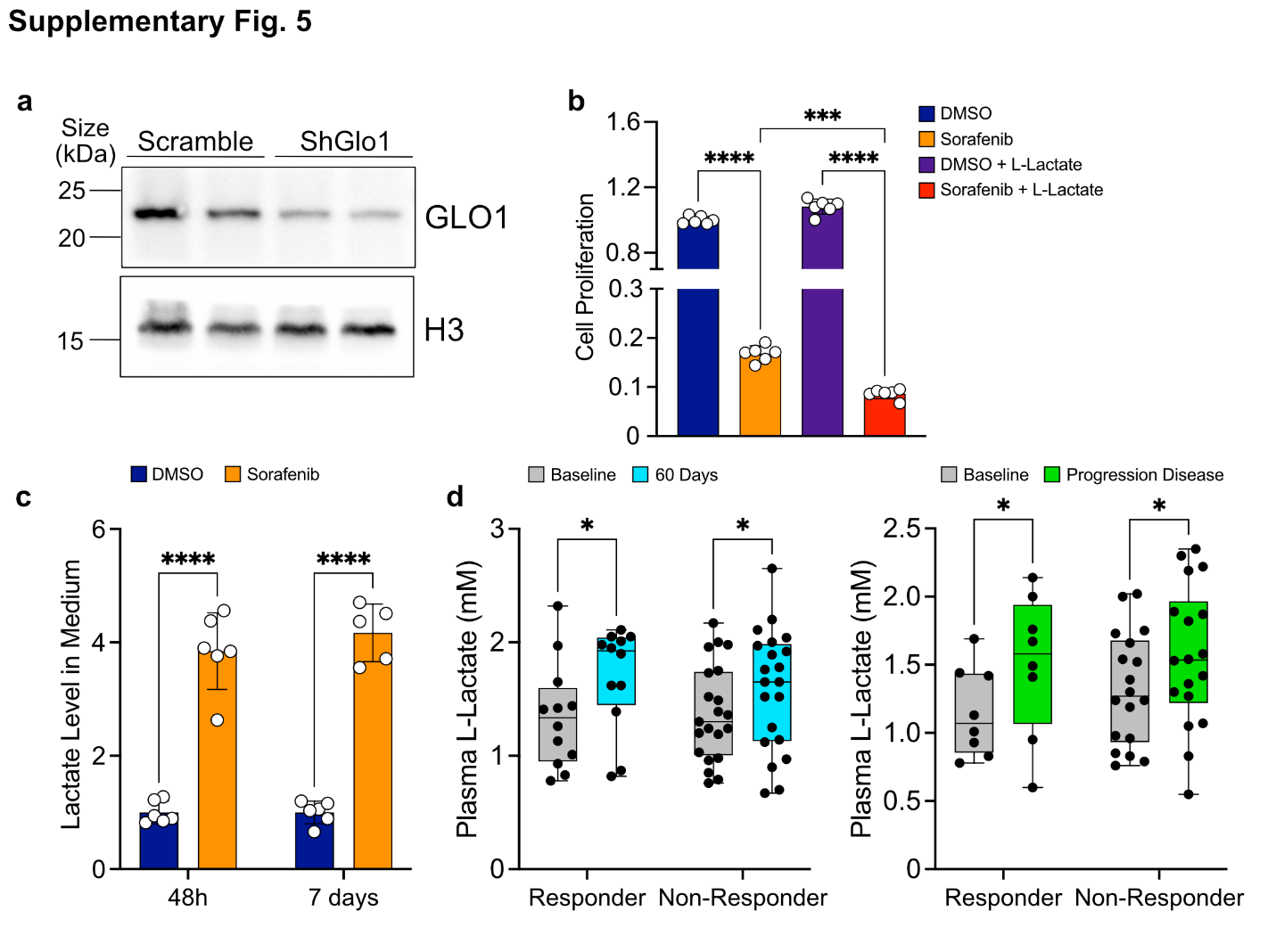
a** GLO1 protein levels in Scramble and ShGlo1 cells.

**b** Cell proliferation following L-lactate treatment (16mM) in combination with either DMSO or sorafenib (8.5µM) for 48 hours. Data are expressed as fold change relative to the DMSO + vehicle group.

**c** Lactate quantification in extracellular media after 48 hours or 7 days treatment with DMSO or sorafenib (8.5µM). The lactate concentration was determined without distinguishing between D- and L-lactate isomers. Lactate amount was normalized on total protein for each sample.

**d** Plasma levels of L-lactate (mM) in HCC patients, comparing responder and non-responder to sorafenib treatment at baseline and after 60 days of administration (left) or at progression disease (right).

For **a**, n=2 samples; **b**, **c**, n=6 samples for 48 hours treatment, n=5 samples for 7 days of sorafenib exposure; **d**, 60 days, n=12 Responder, n=21 Non-Responder patients, PD, n=8 Responder, n=18 Non-Responder patients. Experiments were conducted on *p53*^-/-^; *Myc* hepatoblasts (**a**, **b**, **c**) or using human plasma derived from HCC patients (**d**). Data are represented as mean ± SD, with p-values determined using Ordinary one-way ANOVA corrected for multiple comparisons using Tukey (**b**), multiple t-test corrected for multiple comparisons via the Holm-Sidak method (**c**), or Two-way Repeated Measures ANOVA (**d**). Statistical significance is indicated as follows: * corresponds to p<0.05, *** to p<0.001, and **** to p<0.0001.

Figure. S6.

**
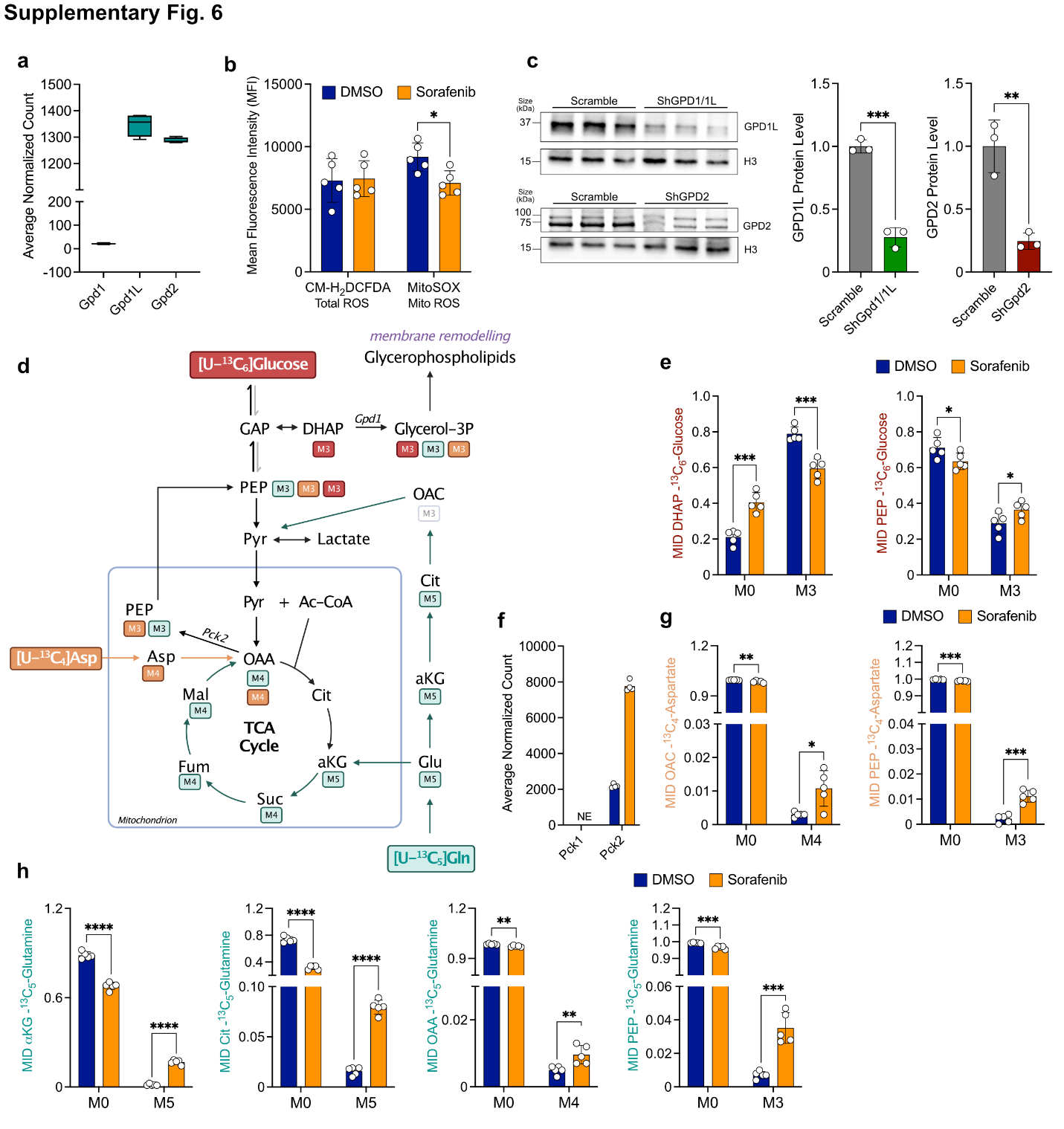
a** Normalized mRNA-seq expression of *Gpd1*, *Gpd1l*, and *Gpd2*, reported as the average counts in control condition.

**b** Quantification of total (CM-H_2_DCFDA probe) and mitochondrial (MitoSOX probe) ROS levels, measured as mean of fluorescence intensity (MFI) in cells treated for 48 hours with DMSO or sorafenib (8.5µM).

**c** Western blot validation of GPD1/1L (upper panel) and GPD2 (lower panel) downregulation following ShRNA treatment, with corresponding quantification on the right.

**d** Schematic representation of tracing analysis using various ^13^C-labeled substrates - glucose, aspartate and glutamine - to track the generation of G3P. Black arrows indicate the metabolism of glucose through glycolysis into the TCA cycle, as well as mitochondrial gluconeogenesis leading to the formation of cytosolic PEP. The final steps of gluconeogenesis are indicated by gray arrows. Green arrows represent the oxidative and reductive pathways of glutamine metabolism. Orange arrows illustrate the internalization and metabolism of aspartate. Boxes displaying different isotopomers (M+n) show the metabolite labeled with n = number of ^13^C atoms within the molecule. If the color matches the metabolite from which the label originates, it indicates significant metabolic changes; if the box is transparent, there is no significant difference between the DMSO and sorafenib-treated conditions. Red boxes correspond to metabolites derived from [U-^13^C_6_]-Glucose, orange from [U-^13^C_4_]-Aspartate and green from [U-^13^C_5_]-Glutamine.

Image created with BioRender.com with permission (agreement number: OZ289LT2S8; citation to use: <https://BioRender.com/l78t783>).

**e** Mass isotopomer distribution (MID) of DHAP or PEP derived from [U-^13^C_6_]-Glucose, after a 6-hour labeling period in cells treated with DMSO or sorafenib (8.5µM) for 48 hours. Sorafenib-treated cells showed reduced M3 DHAP levels and increased M3 PEP, indicating glucose-derived carbons utilization toward both G3P and PEP production.

**f** Normalized mRNA-seq expression of *Pck1* and *Pck2* in *p53^-/-^; Myc* hepatoblasts, reported as average counts in control condition (DMSO) or sorafenib (8.5µM) treatment for 48 hours. (NE = not expressed).

**g** MID of OAA or PEP derived from [U-^13^C_4_]-Aspartate, following a 24-hour labeling period in cells treated with DMSO or sorafenib (8.5µM) for 48 hours. Sorafenib enhanced aspartate transamination to OAA, generating the M4 isotopomer, which is then converted to M3 PEP by *Pck2*.

**h** MID of alpha-ketoglutarate (αKG), citrate (Cit), oxaloacetate (OAA), or phosphoenolpyruvate (PEP) derived from [U-^13^C_5_]-Glutamine, after a 24-hour labeling period in cells treated with DMSO or sorafenib (8.5µM) for 48 hours, indicating that both oxidative (mitochondrial) and reductive (cytosolic) pathways contribute to M3 PEP and G3P generation in sorafenib-treated cells. Both pathways stem from α-ketoglutarate (α-KG), which is processed via transaminase activity to yield the M5 isotopomer. In mitochondria, M5 α-KG is converted to M4 OAA and subsequently to M3 PEP via *Pck2*. In the reductive pathway, M5 α-KG generated M5 citrate, which is cleaved to M3 OAA by ATP citrate lyase and ultimately converted to M3 PEP through pyruvate carboxylase and *Pck2*.

For **b**, **e**, **g**, **h**, n = 5 samples; **a**, **f**, n = 4 samples; **c**, n=3 samples. All the experiments were conducted on *p53*^-/-^; *Myc* hepatoblasts. Data are represented as mean ± SD. Statistical analyses were performed using multiple unpaired t-test (**e**, **g**, **h**), with Holm-Sidak correction (**b**), unpaired t-test (**c**). Statistical significance is indicated as follows: * corresponds to p<0.05, ** to p<0.01, *** to p<0.001, and **** to p<0.0001.

Figure. S7.


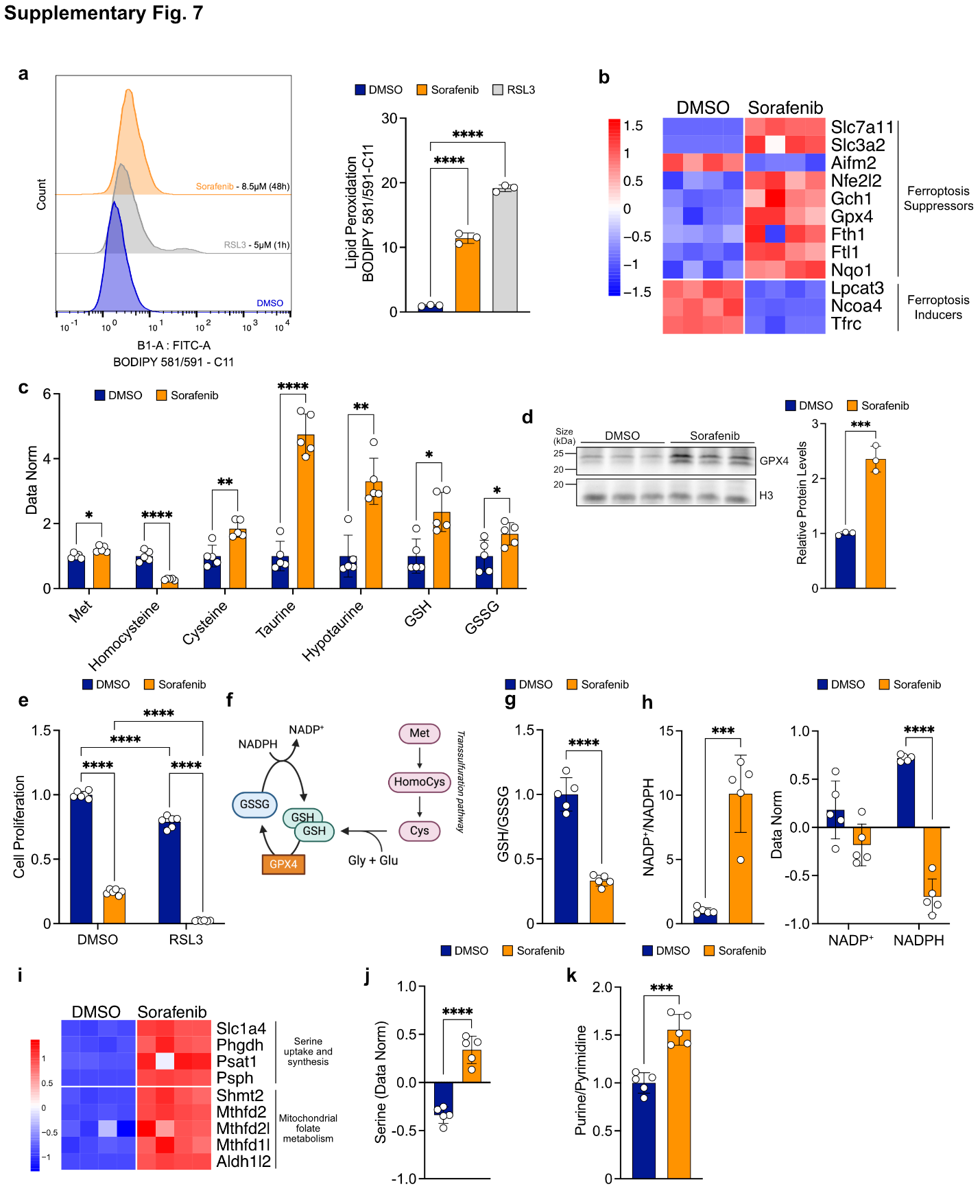
**a** Lipid peroxidation was assessed using BODIPY^TM^ 581/591 C11. Representative flow cytometry histograms illustrating the fluorescence shift in sorafenib-treated (48 hours) and RSL3-treated (positive control, 5 µM for 1 hour) cells compared to the DMSO control (left panel). The right panel showed the quantification of BODIPY^TM^ 581/591 C11-positive cells, indicating increased lipid peroxidation in response to sorafenib and RSL3 treatment.

**b** Heatmap showing the expression of ferroptosis suppressors and inducers after 48 hours of DMSO or sorafenib treatment (8.5µM), including only genes with FDR < 0.05.

**c** Relative quantification of transsulfuration pathway metabolites after 48 hours of DMSO or sorafenib treatment (8.5µM).

**d** GPX4 protein levels after 48 hours of DMSO or sorafenib treatment (8.5µM), with relative quantification (right).

**e** Cell proliferation following RSL3 treatment (GPX4 inhibitor, 25nM) combined with either DMSO or sorafenib (8.5µM) for 48 hours. Data are expressed as fold change relative to the DMSO + vehicle group.

**f** Schematic representation of the transsulfuration pathway’s role in GSH synthesis and redox homeostasis.

Image created with BioRender.com with permission (agreement number: SY289LUDCS; citation to use: <https://BioRender.com/aqith8y>).

**g** GSH/GSSG ratio expressed as fold change relative to DMSO after short-term sorafenib treatment (8.5µM, 48 hours).

**h** NADP^+^/NADPH ratio (fold change relative to DMSO) and normalized NADP^+^ and NADPH levels after 48 hours of DMSO or sorafenib treatment (8.5µM).

**i** Heatmap of genes related to serine uptake, serine synthesis, and mitochondrial folate metabolism after 48 hours of DMSO or sorafenib treatment (8.5µM), showing only genes with FDR < 0.05.

**j** Relative intracellular serine levels after 48 hours of DMSO or sorafenib treatment (8.5µM).

**k** Purine/pyrimidine ratio expressed as fold change relative to DMSO after short-term treatment with sorafenib (8.5µM, 48 hours).

For **a**, **d**, n=3 samples; **b**, **i**, n=4 samples; **c**, **g**, **h**, **j**, **k**, n=5 samples; **e**, n=6 samples. All the experiments were conducted on *p53*^-/-^; *Myc* hepatoblasts. Data are represented as mean ± SD. Statistical significance was assessed using an unpaired t-test (**d**, **g**, **h** left panel, **j**, **k**), two-way ANOVA with Tukey’s correction (**e**), Ordinary one-way ANOVA with Dunnet’s correction (**a**) or multiple t-tests with Holm–Sidak correction (**c**, **h** right panel). Statistical significance is indicated as follows: * corresponds to p<0.05, ** to p<0.01, *** to p<0.001, and **** to p<0.0001.

Figure. S8.

**
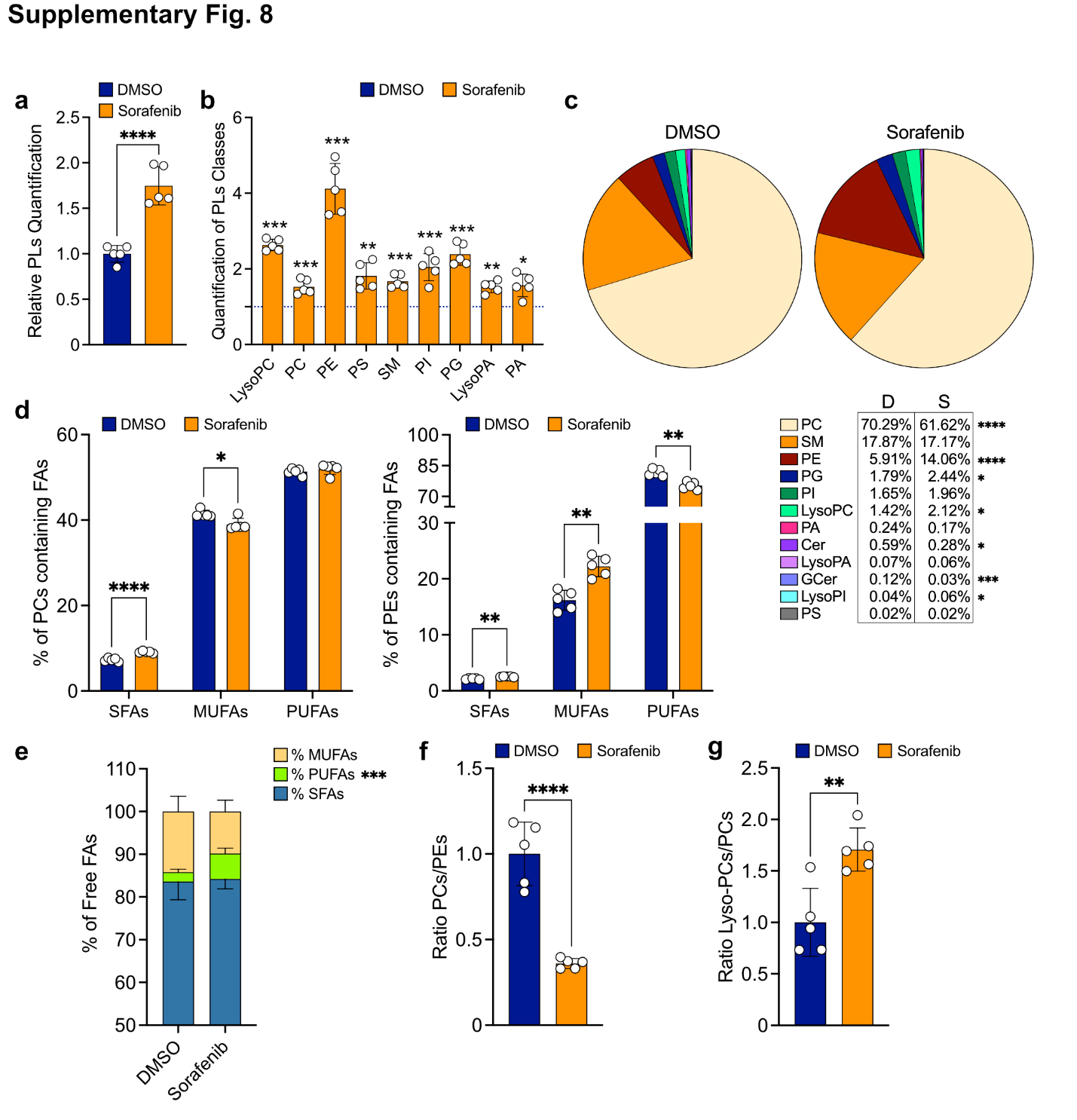
a** Relative quantification of total phospholipids (PLs) detected in cells treated with DMSO or sorafenib for 7 days (8.5µM).

**b** Relative quantification of phospholipids (PLs) by class. The graph shows the fold change relative to control condition, with bars representing sorafenib-treated cells (8.5µM, 7 days) and a dotted line set to 1 corresponding to DMSO-treated cells.

**c** Distribution of phospholipids classes (%) in cells treated with DMSO or sorafenib (8.5µM) for 7 days, shown as a pie chart with values reported in the table below.

**d** Percentage of SFAs, MUFAs, and PUFAs bound in PC (left) and PE (right) after 7 days of sorafenib treatment (8.5µM) compared to DMSO.

**e** Percentage of SFAs, MUFAs, and PUFAs detected as free fatty acids in control and sorafenib-treated cells (8.5µM, 7 days).

**f** Ratio of PCs to PEs after 7 days of DMSO or sorafenib treatment (8.5µM).

**g** Ratio lyso-PCs to PCs after 7 days of DMSO or sorafenib treatment (8.5µM).

For **a**, **b**, **c**, **d**, **e**, **f**, **g**, n=5 samples. All the experiments were conducted on *p53*^-/-^; *Myc* hepatoblasts. Data are represented as mean ± SD, with p-values determined using unpaired t-test (**a**, **f**, **g**), multiple t-test (**c**, **e**), multiple t-test corrected for multiple comparisons via the Holm-Sidak method (**d**), the False Discovery Rate (FDR) method with a two-stage step-up procedure (Benjamini, Krieger, and Yekutieli) (**b**). Statistical significance is indicated as follows: * corresponds to p<0.05, ** to p<0.01, *** to p<0.001, and **** to p<0.0001.

Figure. S9.

**
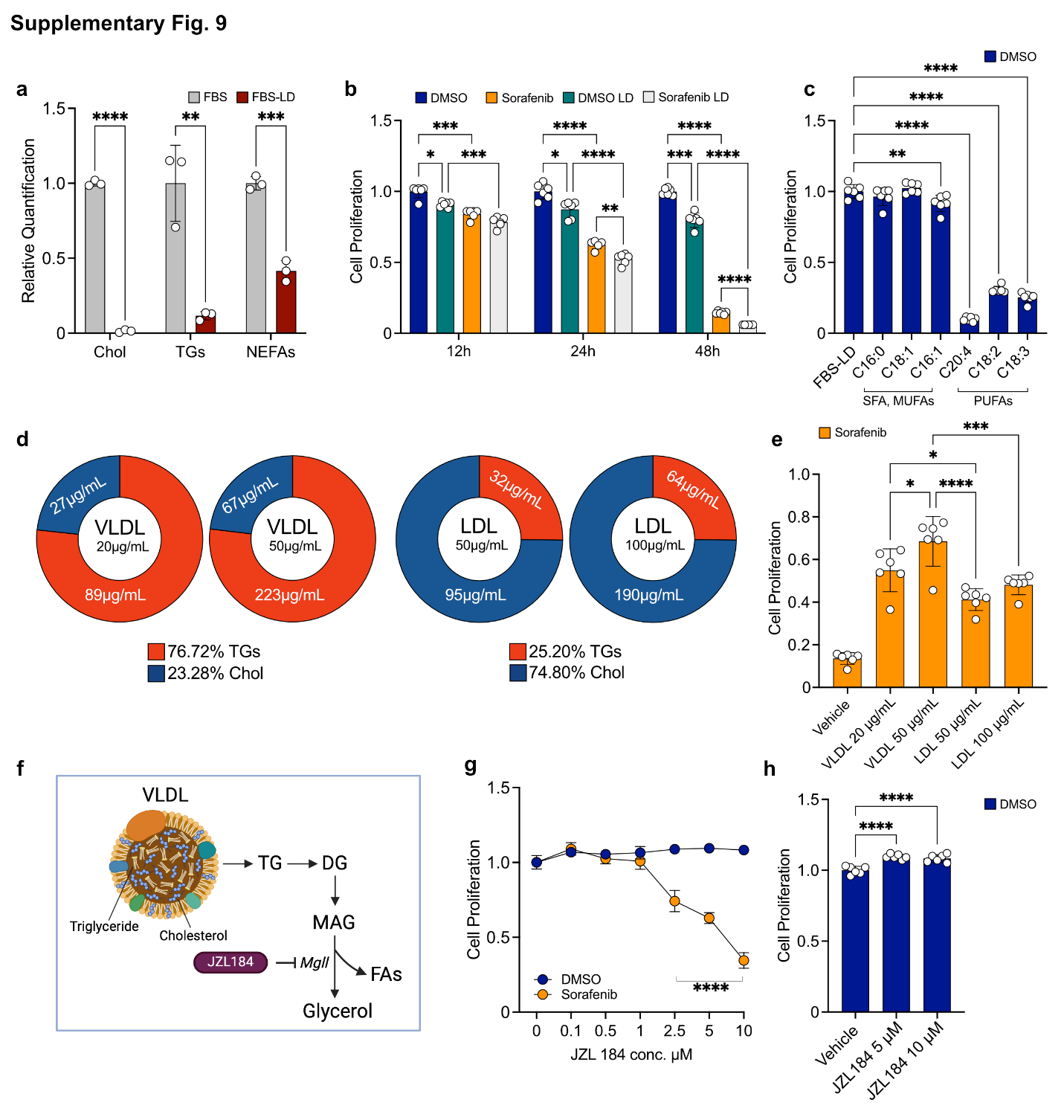
a** Relative quantification of TGs, NEFAs, and cholesterol in FBS-LD compared to FBS.

**b** Kinetic effect on cell viability in culture medium containing 10% FBS-LD in combination with DMSO or 8.5µM sorafenib compared to 10% FBS containing medium. Time points used are 12, 24, and 48 hours.

**c** Cell proliferation following the addition of different fatty acids (SFA, MUFAs, and PUFAs; 100µM) in combination with medium containing 10% FBS-LD and DMSO treatment for 48 hours. Data are expressed as fold change relative to DMSO in FBS-LD.

**d** Quantification of TGs and cholesterol levels, along with their respective percentages, in VLDL and LDL.

**e** Extended statistical analysis from Figure 7d, highlighting the significant effects of VLDL/LDL administration in combination with FBS-LD containing medium and 8.5µM sorafenib treatment (48 hours). Data are expressed as fold change relative to DMSO in FBS-LD containing medium.

**f** Schematic representation of VLDL metabolism, illustrating the release of free fatty acids and glycerol.

Image created with BioRender.com with permission (agreement number: WW289LUS02; citation to use: <https://BioRender.com/f4rjbfl>).

**g** Dose-response effect on cell proliferation following JZL184 (Mgll inhibitor) treatment (from 0.1 to 10µM) in combination with either DMSO or sorafenib (8.5µM) for 48 hours.

**h** Cell proliferation assay with JZL184 (5 or 10µM) in combination with DMSO for 48 hours. Data are expressed as fold change relative to the DMSO + vehicle group.

For **a**, n=3 samples; **b**, **c**, **e**, **g**, **h**, n=6 samples. All the experiments were conducted on *p53*^-/-^; *Myc* hepatoblasts. Data are presented as mean ± SD. Statistical analyses were performed using multiple unpaired t-tests with Holm-Sidak correction (**a**, **g**), two-way RM ANOVA with Tukey’s correction (**b**), ordinary one-way ANOVA with Dunnett’s (**c**, **h**) or Tukey’s correction (**e**). Statistical significance is indicated as follows: * corresponds to p<0.05, ** to p<0.01, *** to p<0.001, and **** to p<0.0001.

**Table. S1.**

Volcano plot - Targeted metabolomic analysis on cells treated with sorafenib for 7 days.

| **Metabolite** | **FC** | **log_2_(FC)** | **raw.pval** | **-LOG_10_(p)** |
| --- | --- | --- | --- | --- |
| Isobutyryl-L-carnitine | 0.0485 | -4.3662 | 0.0000 | 5.5650 |
| Iso-Citrate | 0.0562 | -4.1545 | 0.0000 | 5.2314 |
| Pentanoylcarnitine | 0.0610 | -4.0353 | 0.0000 | 6.1958 |
| Citrate | 0.0923 | -3.4380 | 0.0000 | 5.6196 |
| UTP | 0.1218 | -3.0373 | 0.0001 | 3.8916 |
| GTP | 0.2049 | -2.2867 | 0.0034 | 2.4624 |
| Asp | 0.2129 | -2.2318 | 0.0001 | 4.1448 |
| Dodecanoylcarnitine | 0.2449 | -2.0299 | 0.0003 | 3.5173 |
| CTP | 0.2565 | -1.9629 | 0.0007 | 3.1814 |
| UDP | 0.2802 | -1.8355 | 0.0005 | 3.2753 |
| ITP | 0.2851 | -1.8107 | 0.0024 | 2.6146 |
| L-Acetylcarnitine | 0.2872 | -1.7998 | 0.0014 | 2.8494 |
| Propionylcarnitine | 0.2872 | -1.7998 | 0.0014 | 2.8494 |
| ATP | 0.2907 | -1.7825 | 0.0029 | 2.5364 |
| dGTP | 0.2920 | -1.7758 | 0.0025 | 2.6027 |
| NADPH | 0.3709 | -1.4307 | 0.0013 | 2.8783 |
| Succinate | 0.4334 | -1.2063 | 0.0019 | 2.7282 |
| GSSG | 0.4468 | -1.1624 | 0.4813 | 0.3176 |
| Citrulline | 0.4469 | -1.1619 | 0.0000 | 4.8341 |
| Tetradecanoylcarnitine | 0.4710 | -1.0863 | 0.0000 | 4.7270 |
| O-palmitoleoylcarnitine | 0.5009 | -0.9975 | 0.0006 | 3.2556 |
| Fumarate | 0.5083 | -0.9764 | 0.0038 | 2.4197 |
| Hexanoyl-L-carnitine | 0.5266 | -0.9254 | 0.0023 | 2.6477 |
| SAH | 0.5445 | -0.8771 | 0.1006 | 0.9976 |
| cAMP | 0.5513 | -0.8590 | 0.0680 | 1.1678 |
| Malate | 0.5762 | -0.7954 | 0.0152 | 1.8192 |
| dCTP | 0.6110 | -0.7108 | 0.0260 | 1.5847 |
| GDP | 0.6204 | -0.6888 | 0.0668 | 1.1751 |
| Ornithine | 0.6435 | -0.6360 | 0.0602 | 1.2207 |
| Succinyl-CoA | 0.6584 | -0.6030 | 0.1669 | 0.7776 |
| Oxalacetate | 0.6724 | -0.5726 | 0.0293 | 1.5334 |
| Spermidine | 0.7035 | -0.5075 | 0.8909 | 0.0502 |
| His | 0.7331 | -0.4480 | 0.0298 | 1.5261 |
| Met | 0.7781 | -0.3620 | 0.0630 | 1.2004 |
| Oleoylcarnitine | 0.7842 | -0.3507 | 0.1944 | 0.7114 |
| Tyr | 0.7854 | -0.3486 | 0.0249 | 1.6041 |
| Trp | 0.7870 | -0.3455 | 0.0615 | 1.2113 |
| SAM | 0.8070 | -0.3093 | 0.4238 | 0.3728 |
| ADP | 0.8082 | -0.3072 | 0.0458 | 1.3393 |
| Met SO | 0.8262 | -0.2755 | 0.0206 | 1.6868 |
| Carnitine | 0.8416 | -0.2487 | 0.0512 | 1.2909 |
| dTTP | 0.8538 | -0.2280 | 0.7066 | 0.1508 |
| Acetylornithine | 0.8643 | -0.2104 | 0.1438 | 0.8423 |
| dGDP | 0.8726 | -0.1966 | 0.0758 | 1.1200 |
| OH-Proline | 0.9218 | -0.1175 | 0.2958 | 0.5290 |
| NAD^+^ | 0.9243 | -0.1136 | 0.5085 | 0.2937 |
| dITP | 0.9418 | -0.0865 | 0.7361 | 0.1330 |
| Arg | 0.9435 | -0.0839 | 0.6331 | 0.1985 |
| Glutathione | 0.9487 | -0.0760 | 0.8294 | 0.0812 |
| dUMP | 0.9516 | -0.0716 | 0.8845 | 0.0533 |
| aKG | 0.9610 | -0.0574 | 0.6744 | 0.1711 |
| dCDP | 0.9774 | -0.0330 | 0.9474 | 0.0235 |
| Leu | 0.9819 | -0.0264 | 0.7946 | 0.0999 |
| Val | 0.9955 | -0.0065 | 0.9296 | 0.0317 |
| Phe | 1.0084 | 0.0121 | 0.9115 | 0.0402 |
| Ile | 1.0095 | 0.0137 | 0.9153 | 0.0384 |
| Gly | 1.0102 | 0.0146 | 0.7982 | 0.0979 |
| Asn | 1.0222 | 0.0317 | 0.7899 | 0.1024 |
| Lys | 1.0314 | 0.0446 | 0.6996 | 0.1552 |
| L-Palmitoylcarnitine | 1.0319 | 0.0453 | 0.7990 | 0.0975 |
| Glu | 1.0720 | 0.1004 | 0.2833 | 0.5478 |
| Norepinephrine | 1.0930 | 0.1283 | 0.3835 | 0.4163 |
| Carnosine | 1.0989 | 0.1361 | 0.3406 | 0.4677 |
| Thr | 1.1106 | 0.1514 | 0.0274 | 1.5624 |
| Putrescine | 1.1159 | 0.1583 | 0.4835 | 0.3156 |
| Taurine | 1.1204 | 0.1641 | 0.1173 | 0.9308 |
| CoA | 1.1330 | 0.1802 | 0.3596 | 0.4442 |
| Proline | 1.1433 | 0.1932 | 0.1358 | 0.8671 |
| dIMP | 1.1559 | 0.2091 | 0.6801 | 0.1674 |
| Cysteine | 1.1617 | 0.2162 | 0.1875 | 0.7270 |
| Acetyl Spermidine | 1.1887 | 0.2494 | 0.0051 | 2.2943 |
| Cystine | 1.2160 | 0.2821 | 0.1346 | 0.8711 |
| Acetyl Spermine | 1.2648 | 0.3390 | 0.0333 | 1.4776 |
| Dihydroxyacetone phosphate | 1.2689 | 0.3436 | 0.0170 | 1.7690 |
| Stearoylcarnitine | 1.2716 | 0.3466 | 0.0579 | 1.2375 |
| Ala | 1.2968 | 0.3750 | 0.0021 | 2.6781 |
| Sarcosine | 1.3018 | 0.3805 | 0.0026 | 2.5813 |
| dAMP | 1.3182 | 0.3985 | 0.0715 | 1.1454 |
| Fructose 1,6-bisphosphate | 1.3296 | 0.4110 | 0.0125 | 1.9020 |
| Spermine | 1.3390 | 0.4212 | 0.4084 | 0.3889 |
| CMP | 1.3535 | 0.4367 | 0.0145 | 1.8381 |
| dCMP | 1.3863 | 0.4712 | 0.0170 | 1.7708 |
| UMP | 1.4390 | 0.5251 | 0.0110 | 1.9580 |
| PEP | 1.4786 | 0.5642 | 0.1022 | 0.9905 |
| Pyruvate | 1.5192 | 0.6033 | 0.2330 | 0.6327 |
| Ac-CoA | 1.5243 | 0.6081 | 0.2817 | 0.5502 |
| Ser | 1.5554 | 0.6373 | 0.0001 | 4.2380 |
| NADH | 1.6033 | 0.6811 | 0.0472 | 1.3258 |
| dGMP | 1.7428 | 0.8014 | 0.0014 | 2.8404 |
| dADP | 1.7890 | 0.8392 | 0.0053 | 2.2789 |
| FAD^+^ | 1.8285 | 0.8706 | 0.0704 | 1.1524 |
| AMP | 1.9423 | 0.9578 | 0.0006 | 3.2528 |
| GMP | 2.2198 | 1.1504 | 0.0000 | 5.9368 |
| Glucose 6-Phosphate | 2.2772 | 1.1873 | 0.0010 | 3.0006 |
| Ribu-5P | 2.4965 | 1.3199 | 0.0001 | 4.1024 |
| Erythrose 4-phosphate | 2.6257 | 1.3927 | 0.0001 | 4.0512 |
| NADP | 2.7096 | 1.4381 | 0.0000 | 5.2961 |
| dTMP | 2.9655 | 1.5683 | 0.0014 | 2.8696 |
| Sedoheptulose 7-phosphate | 3.6478 | 1.8670 | 0.0000 | 5.6161 |
| Lactate | 3.7711 | 1.9150 | 0.0005 | 3.2918 |
| Gluconate 6P | 5.1364 | 2.3607 | 0.0031 | 2.5077 |
| IMP | 6.6800 | 2.7398 | 0.0000 | 4.9574 |
| Glucose | 9.3901 | 3.2311 | 0.0005 | 3.3310 |
| Glutamine | 13.3170 | 3.7352 | 0.0002 | 3.7432 |

Metabolites decreased in sorafenib-treated cells are shown in red, while those increased after 7 days of treatment are shown in blue. Statistically significant changes (p < 0.05) are highlighted in dark red. Data are ordered by Fold Change (FC).

Table. S2.

| **Metabolite/Lipid Class** | **MS Instrument** | **Ionization Mode** | **Column/Separation** | **Mobile Phase** | **HPLC Methods** | **Flow Rate** | **Detection Mode** | **Derivatization** | **Quantification Method** |
| --- | --- | --- | --- | --- | --- | --- | --- | --- | --- |
| Energetic metabolites (Glycolysis, TCA cycle, Pentose Phosphate Pathway), Cofactors (NAD+, NADPH), Nucleotides (ATP, ADP, AMP) | Triple Quad 3500 (Sciex) | ESI neg | Luna CN, 5µm, 100Å, LC Column, 50 x 4.6mm | A: H_2_O , B: 2mM NH_4_CH_3_CO_2_ in MeOH | Isocratic (50%) | 0.6mL/min | MRM | No | Metaboanalyst |
| Amino Acids and Biogenic Amines | Triple Quad 3500 (Sciex) | ESI pos | C18 column | A: 0.2% HCOOH in H_2_O, B: 0.2% HCOOH in ACN | Gradient: T0min 100%A, T5.5min 5%A, T7min 100%A | 0.5mL/min | MRM | Yes, 5% PITC in EtOH, Pyridine, H_2_O (1:1:1, v/v) | Metaboanalyst |
| Acyl-Carnitine, SAM/SAH and GSH/GSSG | Triple Quad 3500 (Sciex) | ESI pos | ZORBAX Stable Bond CN, 5μm, LC Column, 2.1 x 150mm | A: 0.2% HCOOH in H_2_O, B: 0.2% HCOOH in ACN | Gradient: T0min 100%A, T5.5min 5%A, T7min 100%A | 0.3mL/min | MRM | No | Metaboanalyst |
| Phopholipids (Cer, PE, SP, LysoPC, PC, SM) | Triple Quad 4000 (Sciex) | ESI pos | XTerra RP18, 3.5 µm, LC Column, 100 × 4.6mm | 0.1% HCOOH in MeOH | Isocratic | 0.5mL/min | MRM | No | Pure Standards |
| Phopholipids (PG, PA, PI) | Triple Quad 4000 (Sciex) | ESI neg | Luna CN, 5µm, 100Å, LC Column, 50 x 4.6mm | 5mM NH_4_CH_3_CO_2_ in MeOH | Isocratic | 0.5mL/min | MRM | No | Pure Standards |
| Fatty Acids | Triple Quad 4000 (Sciex) | ESI neg | Hypersil Gold C8, 3μm, LC Column, 100 × 3mm | A: 10mM DIPEA, 15mM CH₃COOH in H_2_O/MeOH 97:3 and B: MeOH | Gradient: T0min 20%A, T20min 1%A, T25min 1%A, T25.1min 20%A, T30min 20%A | 0.5mL/min | SIM/SIM | No | Pure Standards |
| Transsulfuration Pathway Metabolites | Triple Quad 3500 (Sciex) | ESI pos | Raptor Polar X, 2.7 μm, 90 Å, LC Column, 100 x 2.1mm | A: 0.5% HCOOH, 1mM NH_4_HCO_2_ in H_2_O, B: 0.5% HCOOH, 1mM NH4HCO2 in ACN/H_2_O (90:10) | Gradient: T0min 4%A, T2min 4%A, T10min 70%A, T10.01min 95%A, T11min 95%A, T11.01min 4%A, T13min 4%A | 0.3mL/min | MRM | No | Pure Standards |
| D-/L-lactate | QTOF X500R (Sciex) | ESI neg | Astec CHIROBIOTIC® R Chiral Column, 5µm, 150 x 2.1mm | 15% (v/v) 33.3mM NH_4_CH_3_CO_2_ in H_2_O and 85% (v/v) ACN | Isocratic | 0.7mL/min | MRM | No | Pure Standards |
| Coenzyme Q | Triple Quad 3500 (Sciex) | ESI pos | Kinetex C18 column,2.6 μm, 2.1 × 50 mm | 2mM NH_4_HCO_2_ in MeOH | Isocratic | 0.350mL/min | MRM | No | Pure Standards |
| Untargeted Lipidomics | QTOF X500R (Sciex) | ESI neg/pos | Kinetex F5 column, 2.6µm, 100 Å, 150 × 2.1mm | A: 0.1% HCOOH in H_2_O, B: 0.1% HCOOH in ACN | Gradient: T0min 99.8% A, T2min 99.8% A, T5min 98% A, T11min 75% A, T13min 2% A, T17min 2% A, T18min 99.8% A, T20min 99.8%A | 0.2mL/min | SWATH-DIA | No | MS-DIAL |

Comprehensive overview of the LC-MS methods used for compounds quantification presented in the results section. The table includes detailed information on the metabolite or lipid class analyzed, MS instrument used, ionization mode, chromatographic column and separation conditions, mobile phase composition, HPLC method parameters, flow rate, detection mode, derivatization (if applicable), and the quantification method, based either on comparison to authentic standards or on relative quantification using normalized peak areas.
