# Supplementary material for: D-lactate and glycerol as potential biomarkers of sorafenib activity in hepatocellular carcinoma
Source: Signal Transduct Target Ther. 2025 Jun 27;10:200. doi: 10.1038/s41392-025-02282-z (PMC12202795; doi:10.1038/s41392-025-02282-z)
Supplement: Supplementary file 2 — Uncropped Western blot [file 41392_2025_2282_MOESM2_ESM.pptx]

## Slide 1
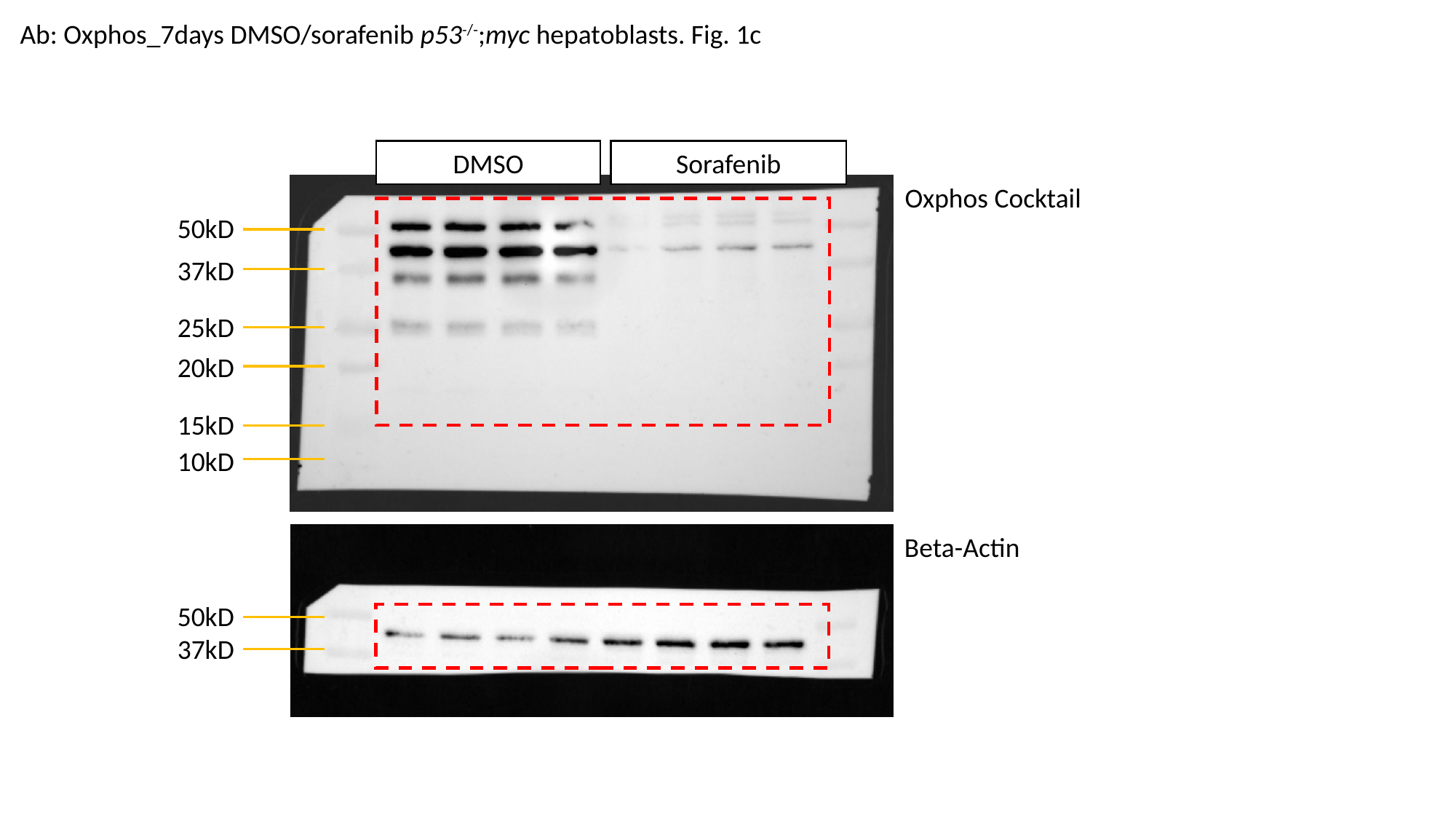

Ab: Oxphos_7days DMSO/sorafenib p53-/-;myc hepatoblasts. Fig. 1c
DMSO
Sorafenib
Oxphos Cocktail
50kD
37kD
25kD
20kD
15kD
10kD
Beta-Actin
50kD
37kD

## Slide 2
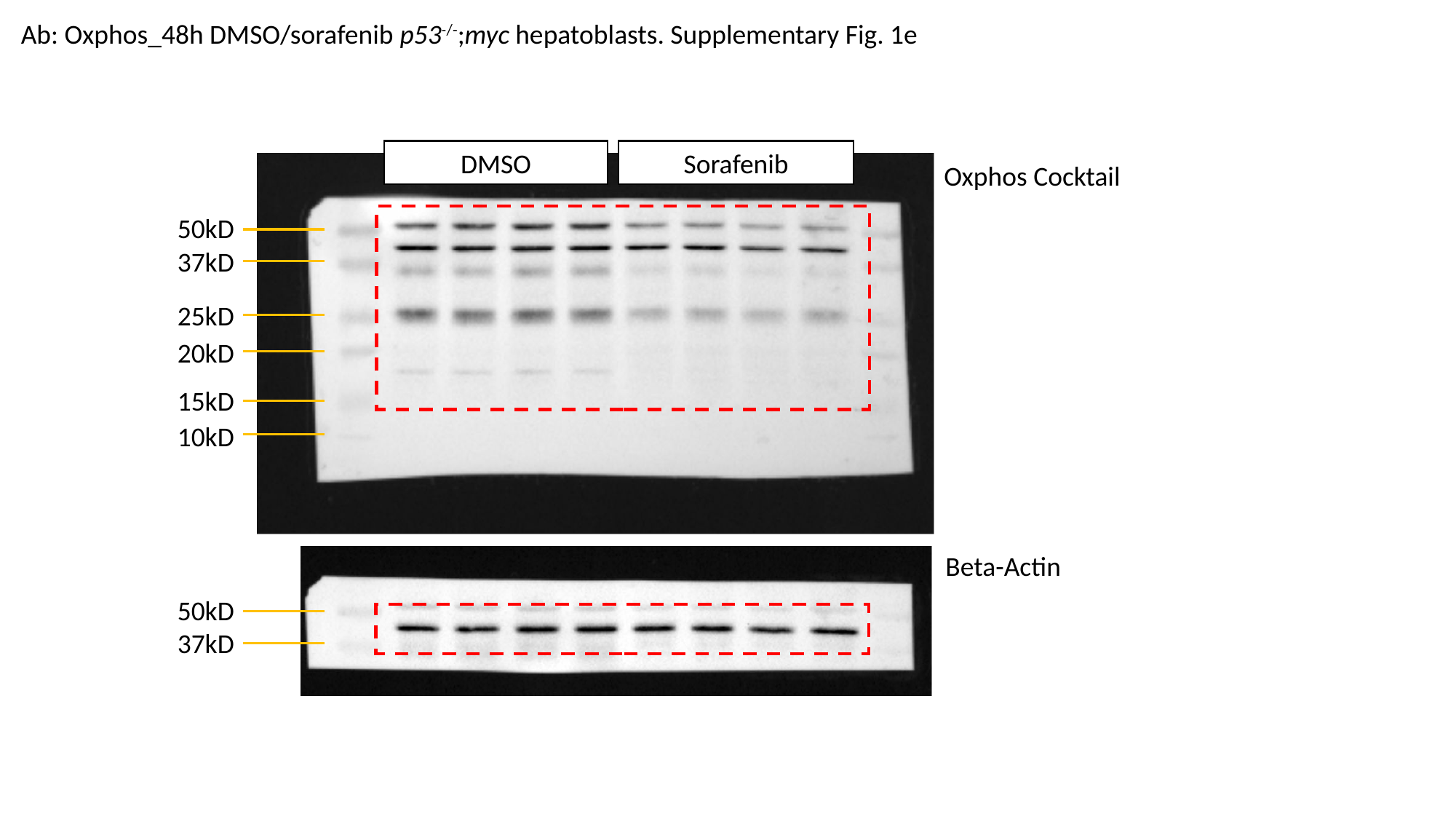

Ab: Oxphos_48h DMSO/sorafenib p53-/-;myc hepatoblasts. Supplementary Fig. 1e
DMSO
Sorafenib
Oxphos Cocktail
50kD
37kD
25kD
20kD
15kD
10kD
Beta-Actin
50kD
37kD

## Slide 3
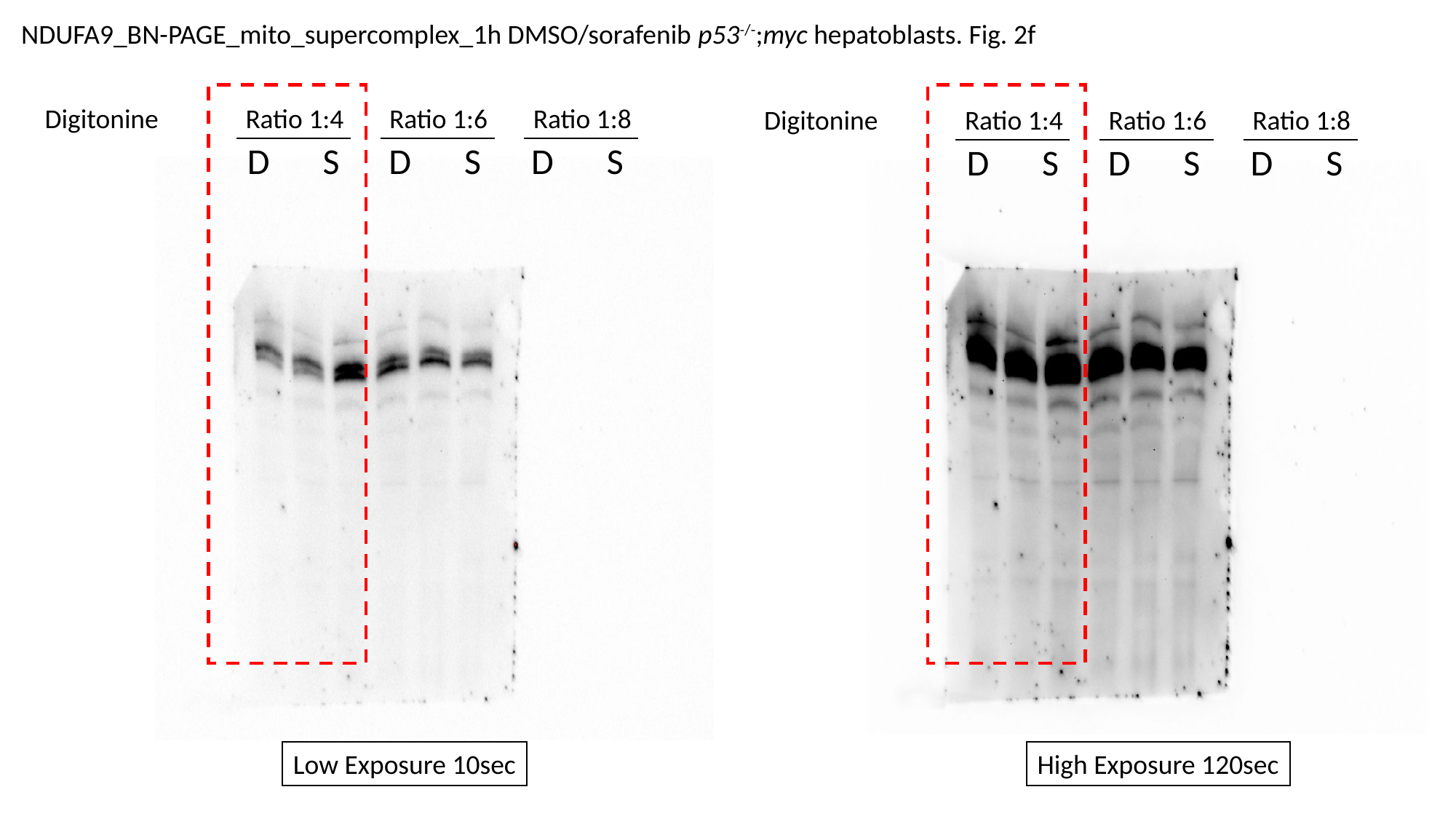

NDUFA9_BN-PAGE_mito_supercomplex_1h DMSO/sorafenib p53-/-;myc hepatoblasts. Fig. 2f
Digitonine
Ratio 1:4
Ratio 1:6
Ratio 1:8
Digitonine
Ratio 1:4
Ratio 1:6
Ratio 1:8
D
S
D
S
D
S
D
S
D
S
D
S
Low Exposure 10sec
High Exposure 120sec

## Slide 4
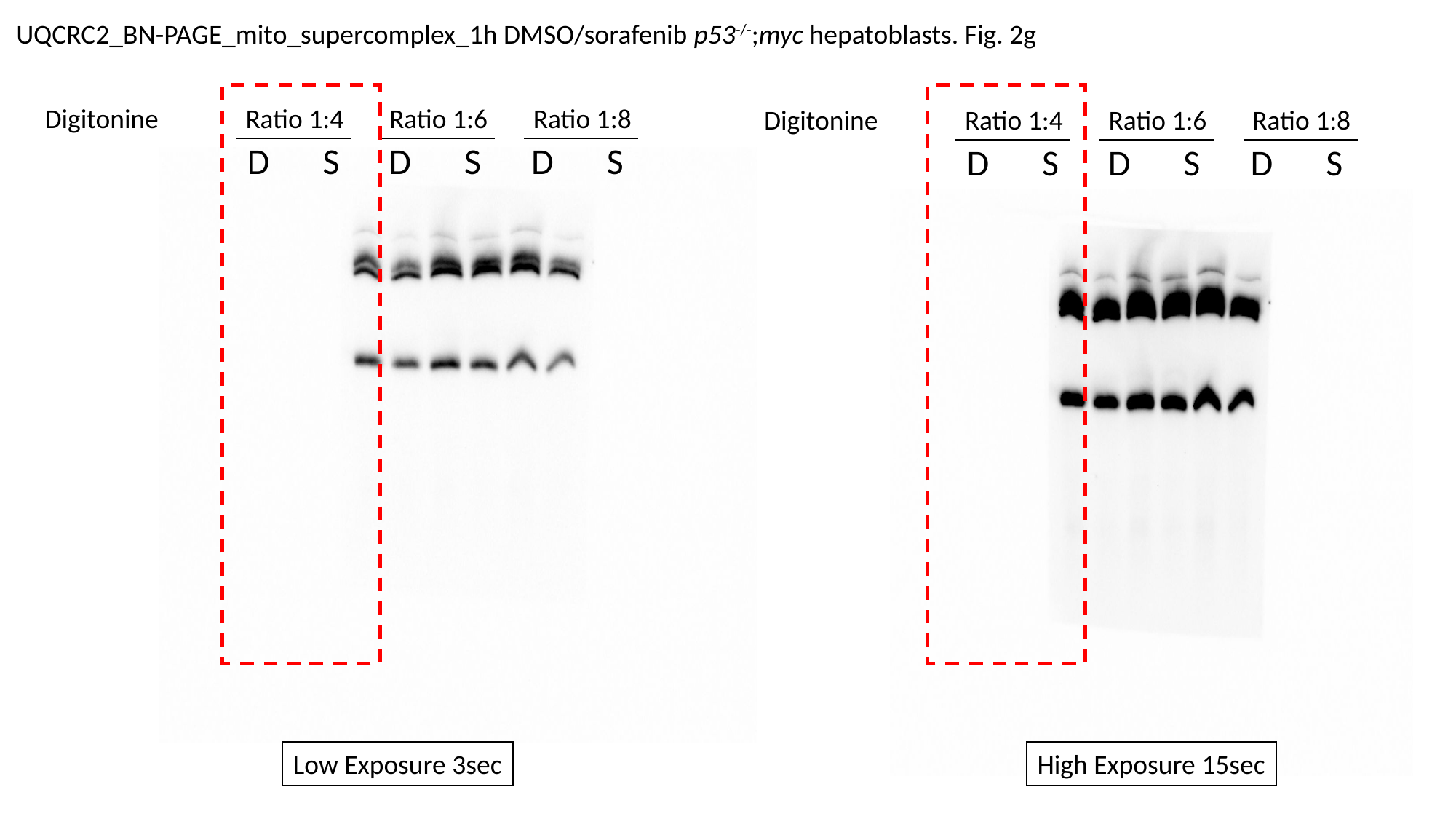

UQCRC2_BN-PAGE_mito_supercomplex_1h DMSO/sorafenib p53-/-;myc hepatoblasts. Fig. 2g
Digitonine
Ratio 1:4
Ratio 1:6
Ratio 1:8
Digitonine
Ratio 1:4
Ratio 1:6
Ratio 1:8
D
S
D
S
D
S
D
S
D
S
D
S
Low Exposure 3sec
High Exposure 15sec

## Slide 5
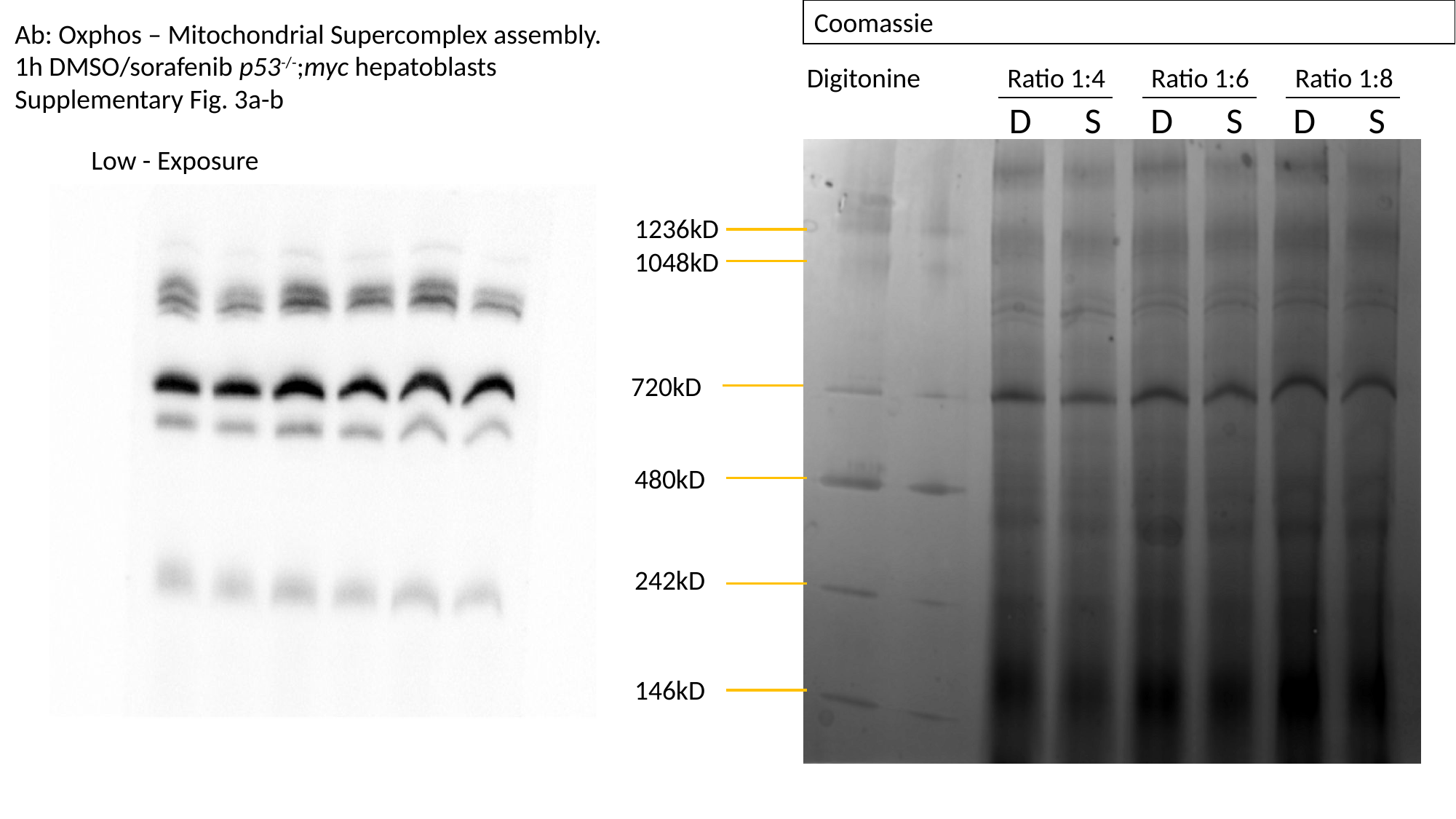

Coomassie
Ab: Oxphos – Mitochondrial Supercomplex assembly.
1h DMSO/sorafenib p53-/-;myc hepatoblasts
Supplementary Fig. 3a-b
Digitonine
Ratio 1:4
Ratio 1:6
Ratio 1:8
D
S
D
S
D
S
Low - Exposure
1236kD
1048kD
720kD
480kD
242kD
146kD

## Slide 6
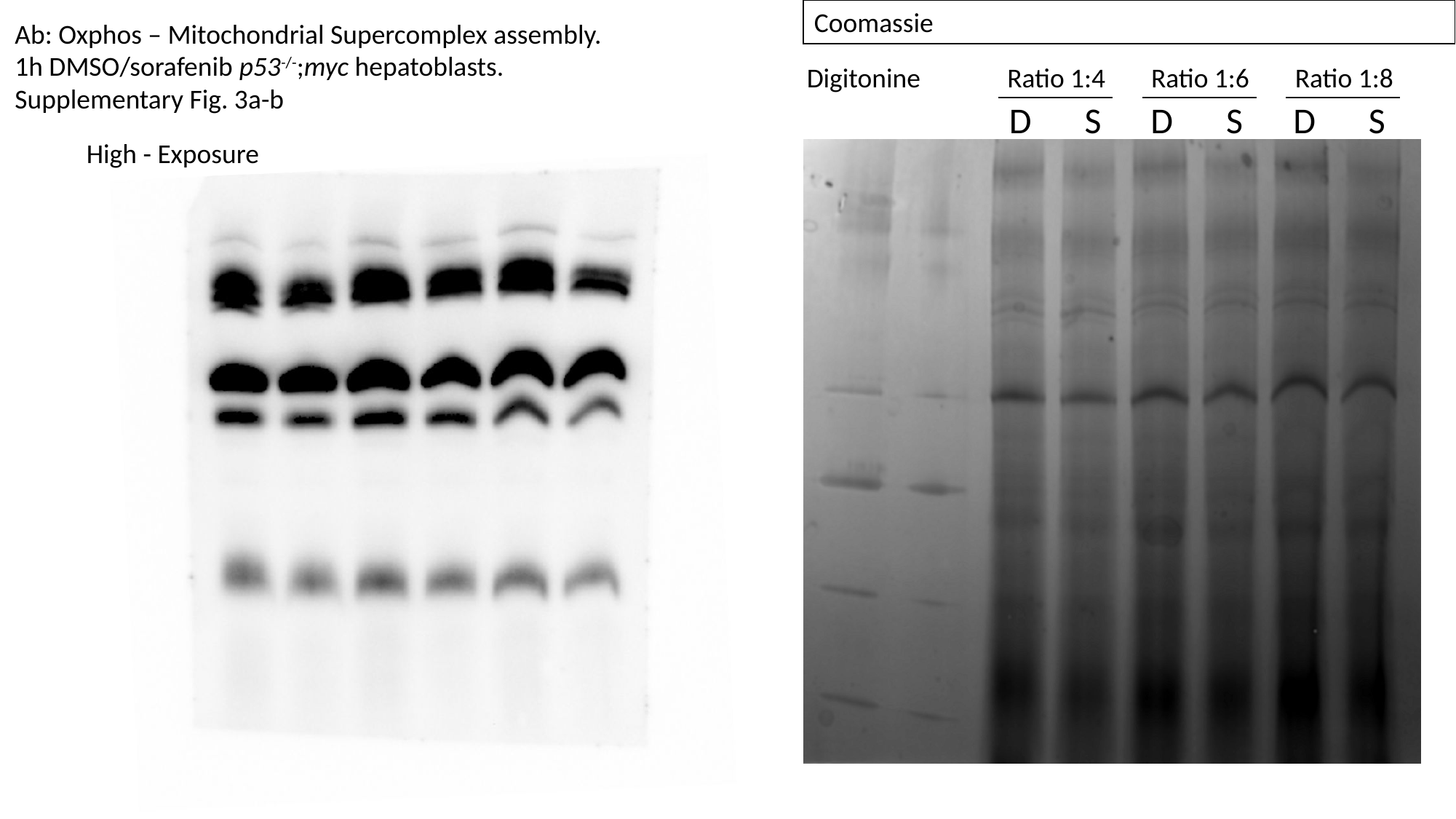

Coomassie
Ab: Oxphos – Mitochondrial Supercomplex assembly.
1h DMSO/sorafenib p53-/-;myc hepatoblasts.
Supplementary Fig. 3a-b
Digitonine
Ratio 1:4
Ratio 1:6
Ratio 1:8
D
S
D
S
D
S
High - Exposure

## Slide 7
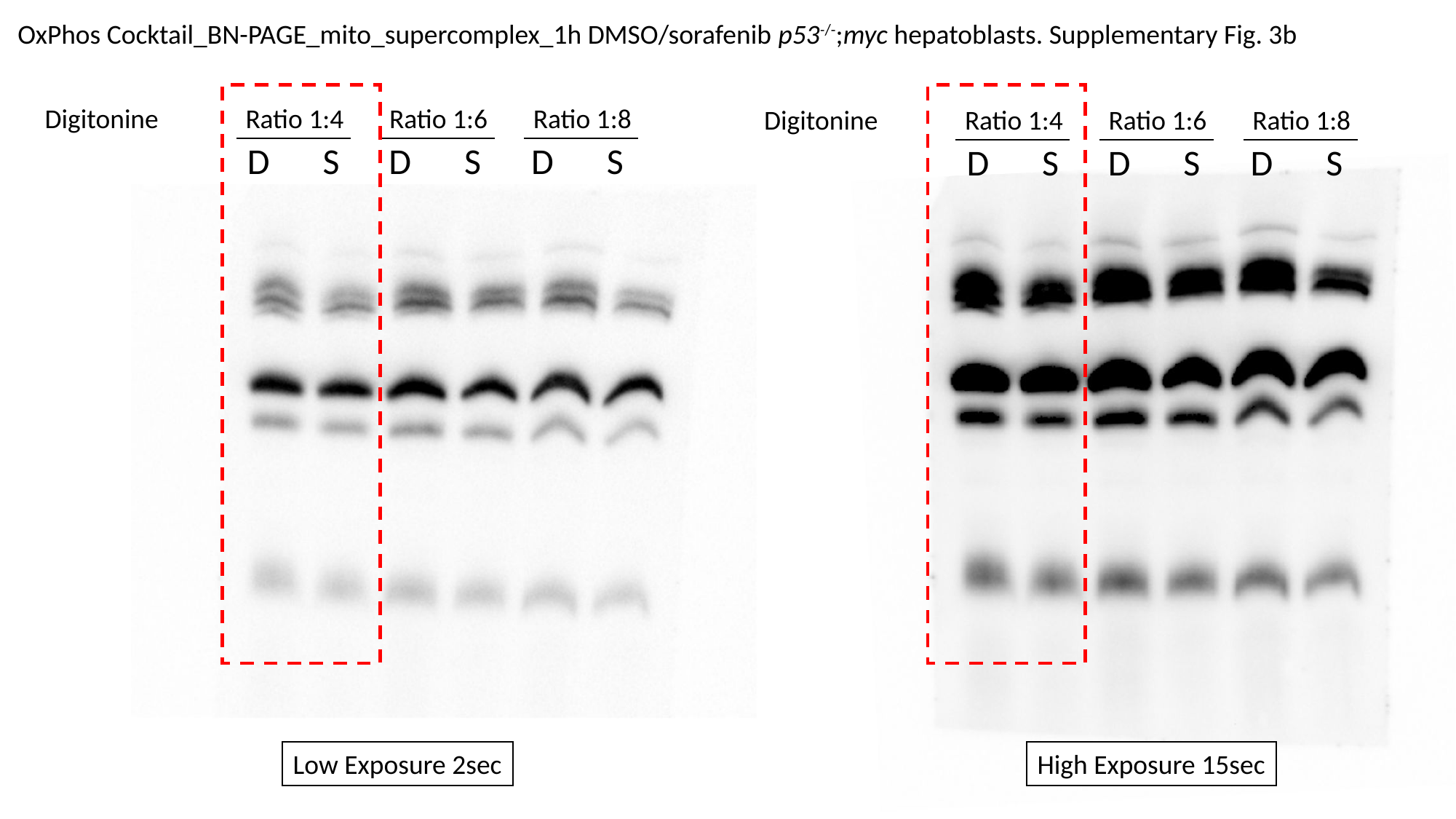

OxPhos Cocktail_BN-PAGE_mito_supercomplex_1h DMSO/sorafenib p53-/-;myc hepatoblasts. Supplementary Fig. 3b
Digitonine
Ratio 1:4
Ratio 1:6
Ratio 1:8
Digitonine
Ratio 1:4
Ratio 1:6
Ratio 1:8
D
S
D
S
D
S
D
S
D
S
D
S
Low Exposure 2sec
High Exposure 15sec

## Slide 8
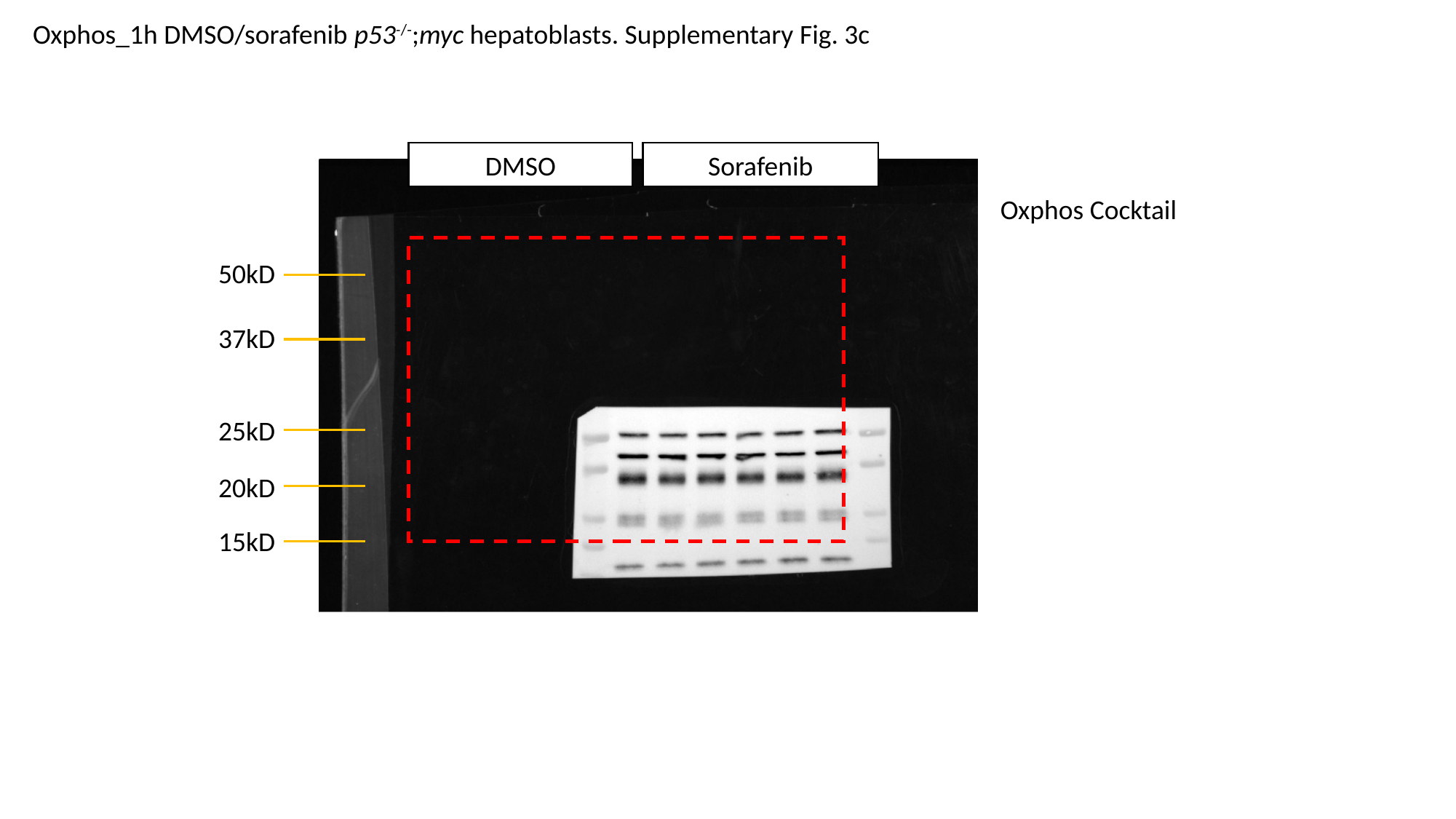

Oxphos_1h DMSO/sorafenib p53-/-;myc hepatoblasts. Supplementary Fig. 3c
DMSO
Sorafenib
Oxphos Cocktail
50kD
37kD
25kD
20kD
15kD

## Slide 9
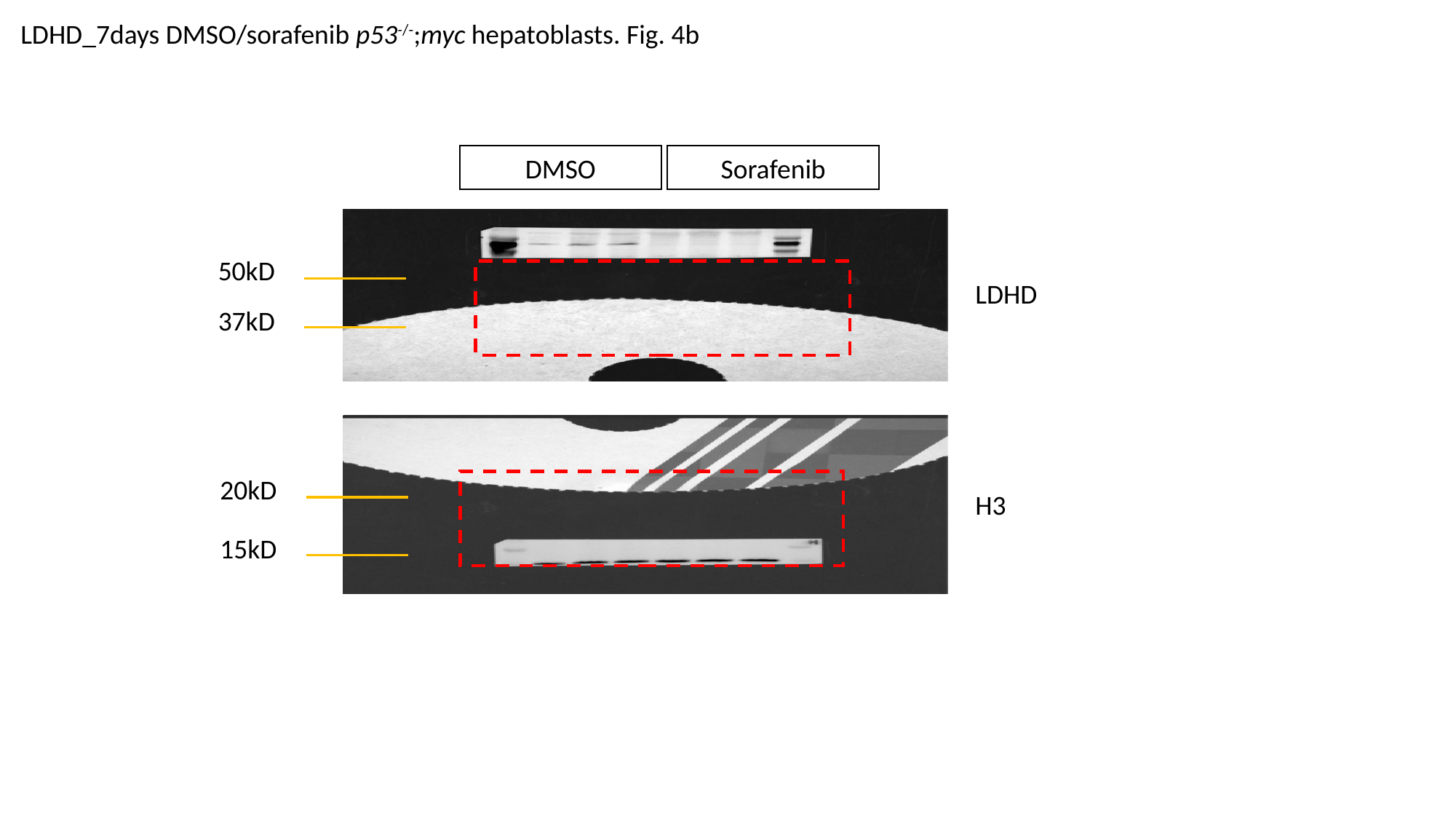

LDHD_7days DMSO/sorafenib p53-/-;myc hepatoblasts. Fig. 4b
DMSO
Sorafenib
50kD
LDHD
37kD
20kD
H3
15kD

## Slide 10
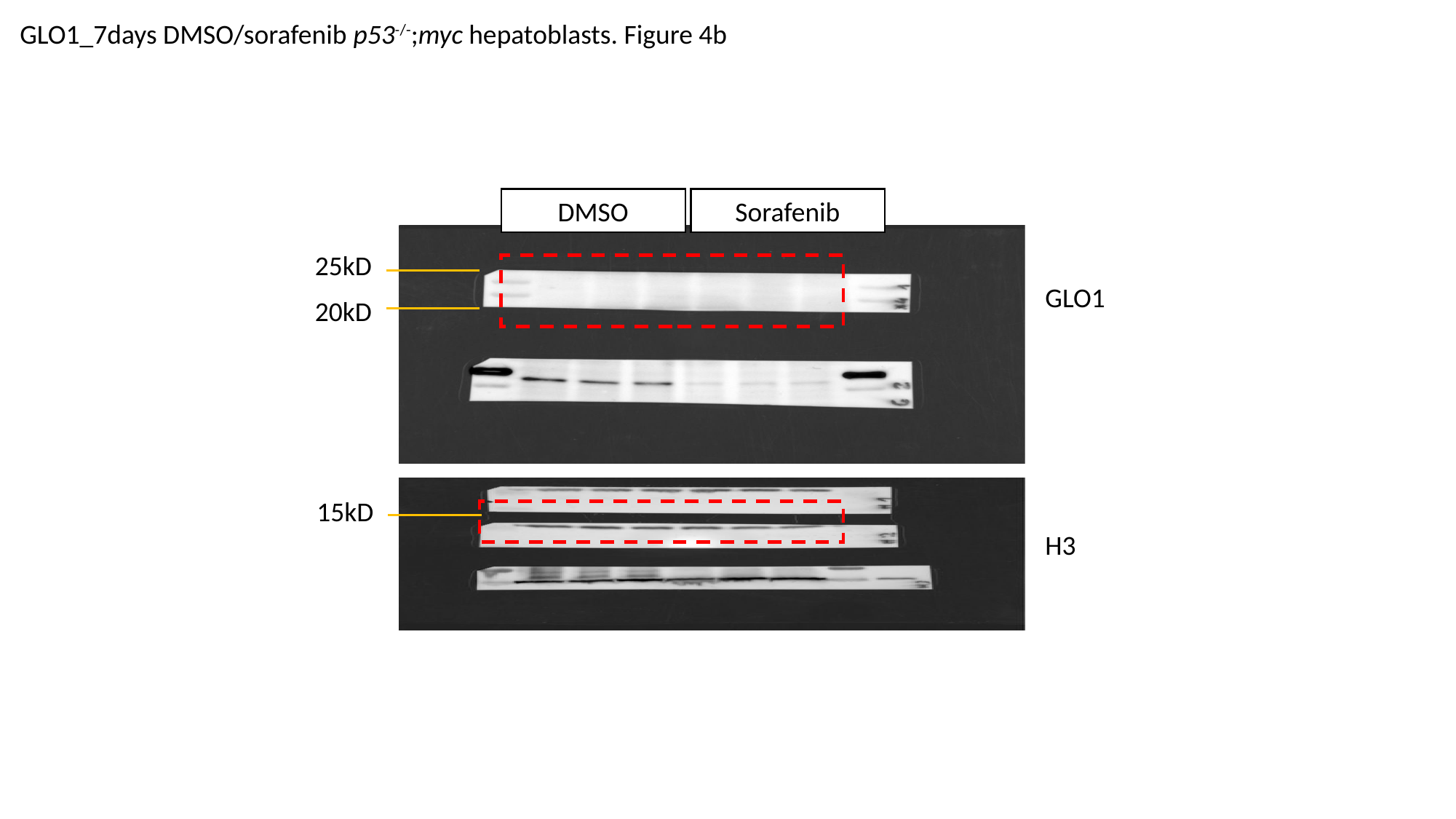

GLO1_7days DMSO/sorafenib p53-/-;myc hepatoblasts. Figure 4b
DMSO
Sorafenib
25kD
GLO1
20kD
15kD
H3

## Slide 11
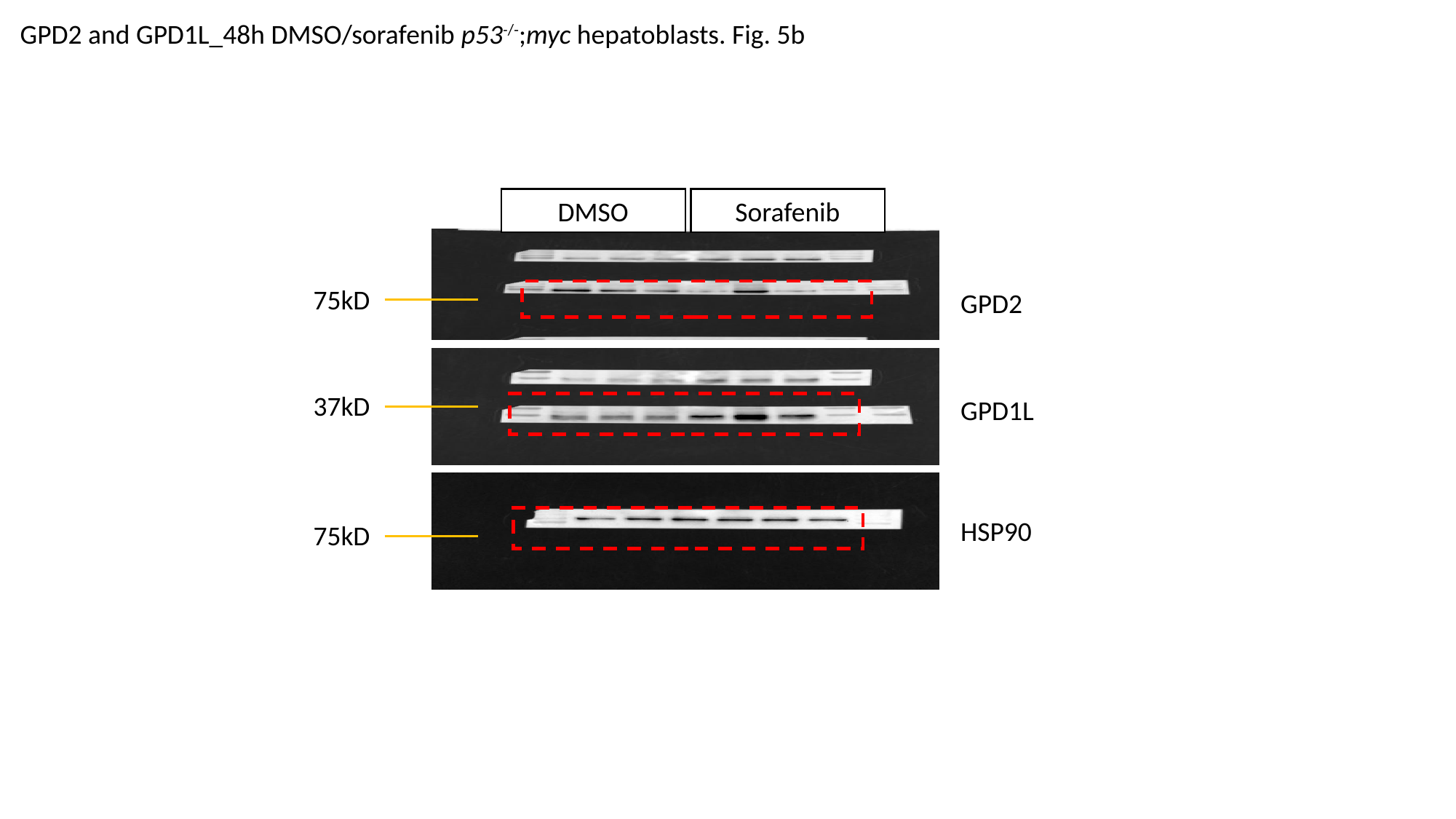

GPD2 and GPD1L_48h DMSO/sorafenib p53-/-;myc hepatoblasts. Fig. 5b
DMSO
Sorafenib
75kD
GPD2
37kD
GPD1L
HSP90
75kD

## Slide 12
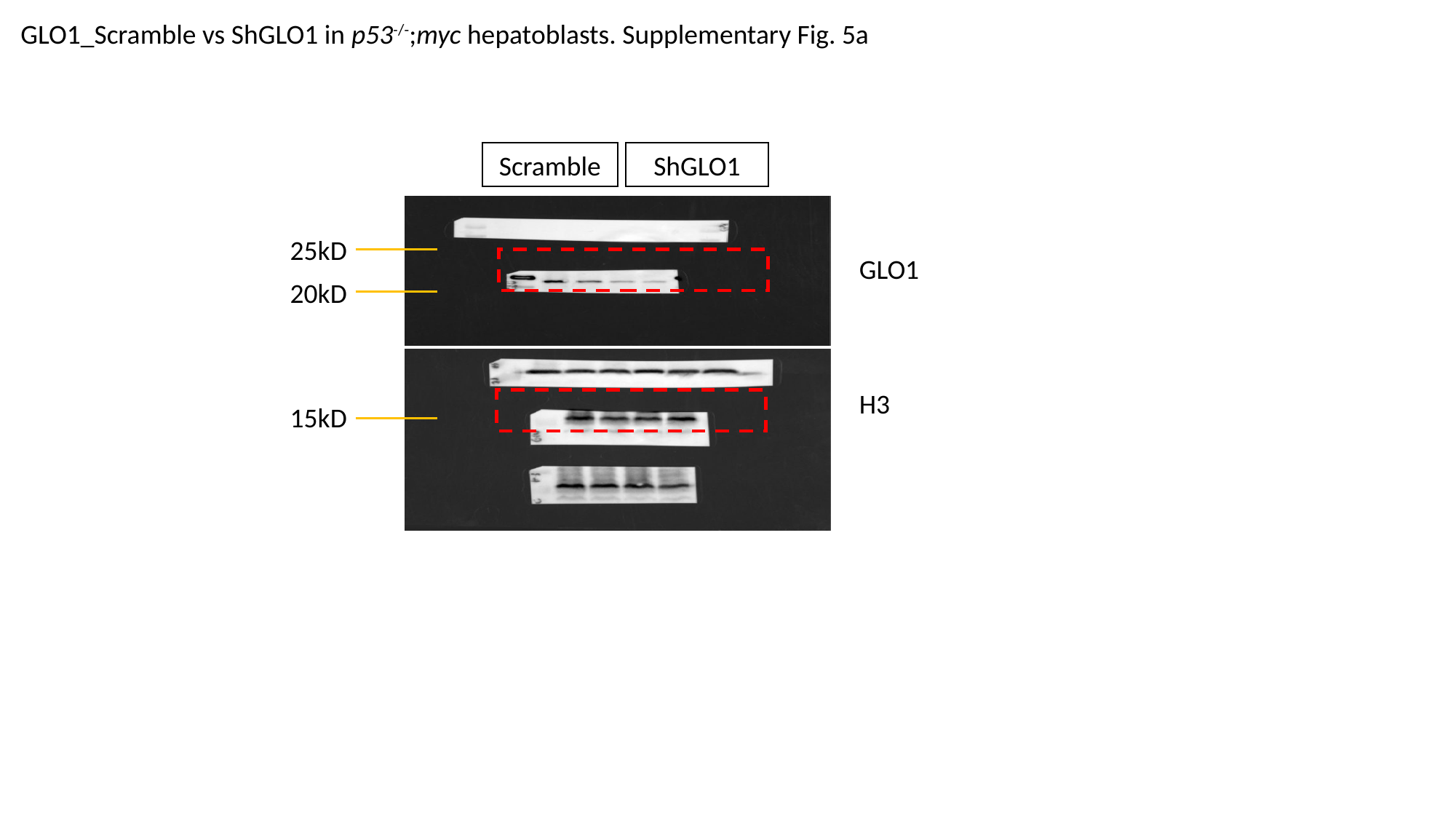

GLO1_Scramble vs ShGLO1 in p53-/-;myc hepatoblasts. Supplementary Fig. 5a
Scramble
ShGLO1
25kD
GLO1
20kD
H3
15kD

## Slide 13
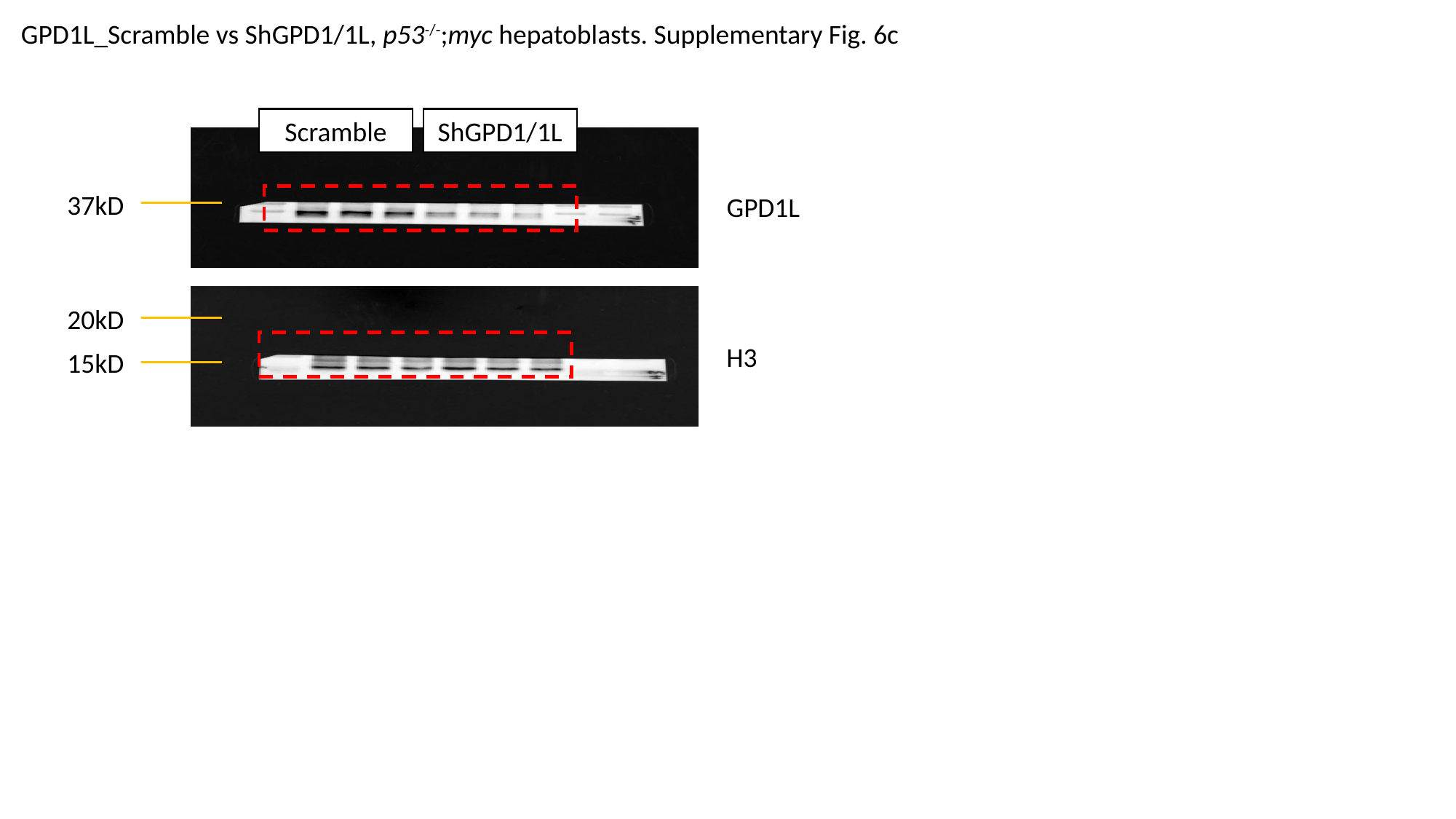

GPD1L_Scramble vs ShGPD1/1L, p53-/-;myc hepatoblasts. Supplementary Fig. 6c
Scramble
ShGPD1/1L
37kD
GPD1L
20kD
H3
15kD

## Slide 14
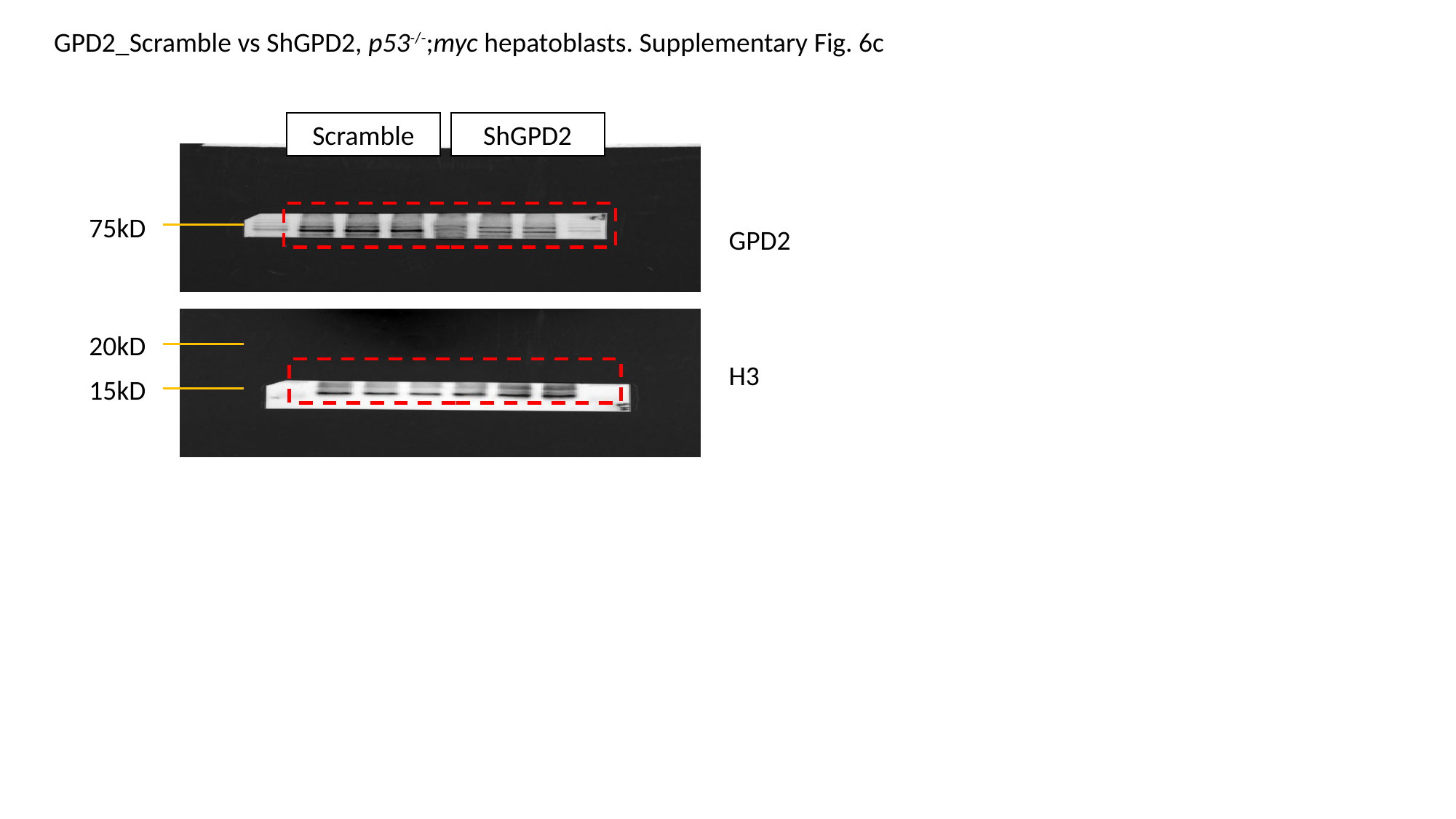

GPD2_Scramble vs ShGPD2, p53-/-;myc hepatoblasts. Supplementary Fig. 6c
Scramble
ShGPD2
75kD
GPD2
20kD
H3
15kD

## Slide 15
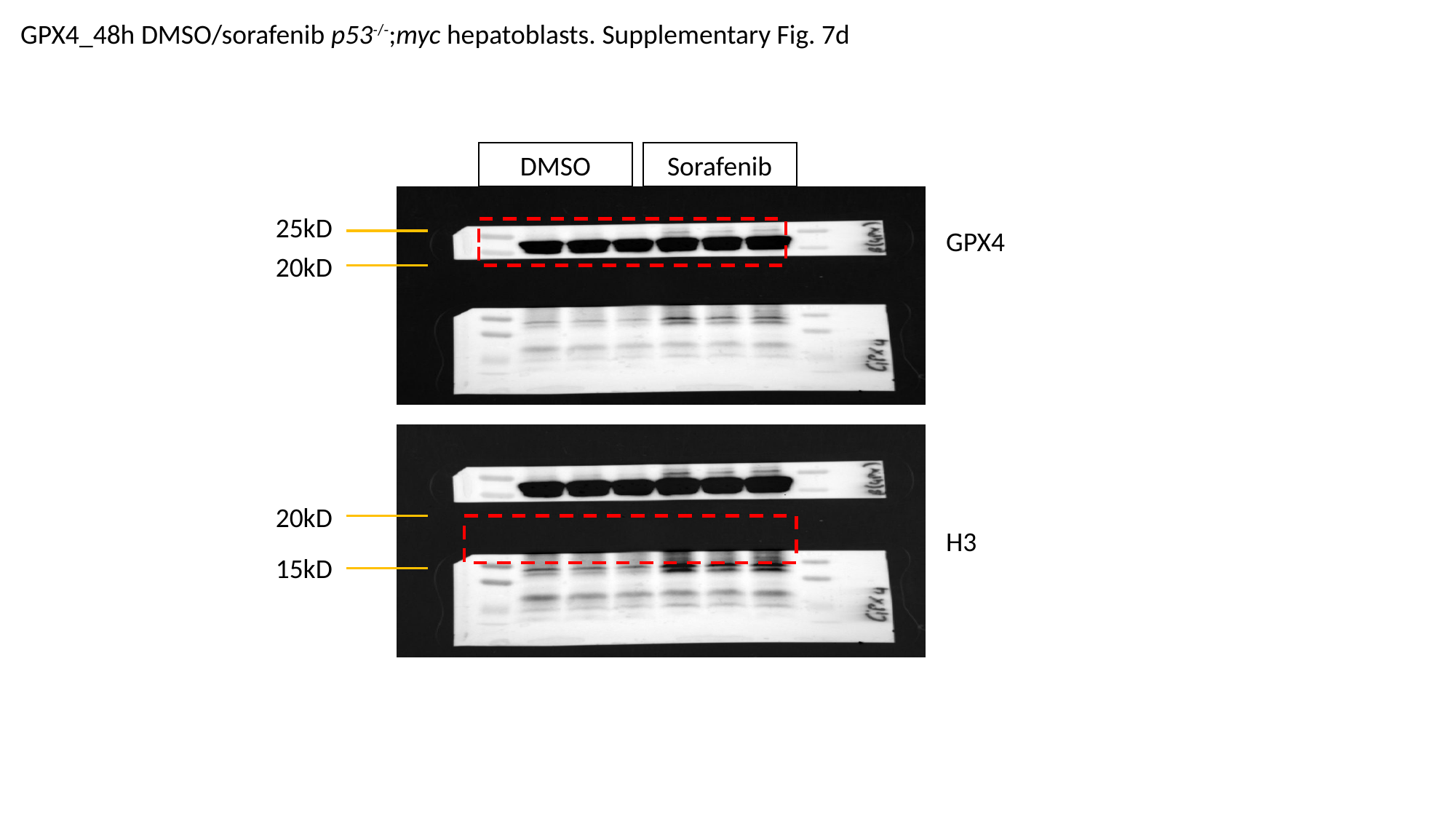

GPX4_48h DMSO/sorafenib p53-/-;myc hepatoblasts. Supplementary Fig. 7d
DMSO
Sorafenib
25kD
GPX4
20kD
20kD
H3
15kD
