# Supplementary material for: D-lactate and glycerol as potential biomarkers of sorafenib activity in hepatocellular carcinoma
Source: Signal Transduct Target Ther. 2025 Jun 27;10:200. doi: 10.1038/s41392-025-02282-z (PMC12202795; doi:10.1038/s41392-025-02282-z)
Supplement: Supplementary file 3 — Gating strategies for flow cytometry analysis [file 41392_2025_2282_MOESM3_ESM.pptx]

## Slide 1
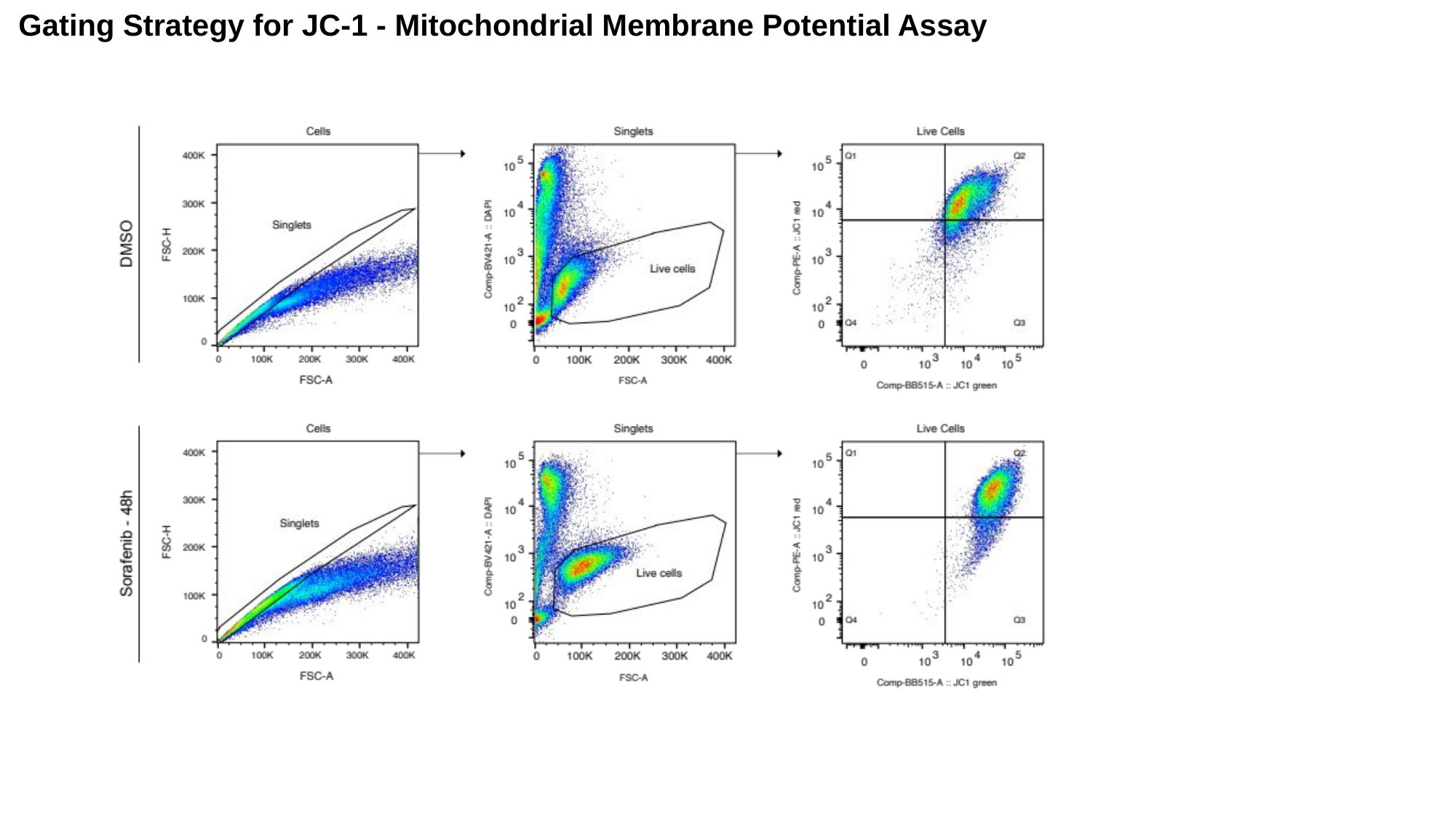

Gating Strategy for JC-1 - Mitochondrial Membrane Potential Assay

## Slide 2
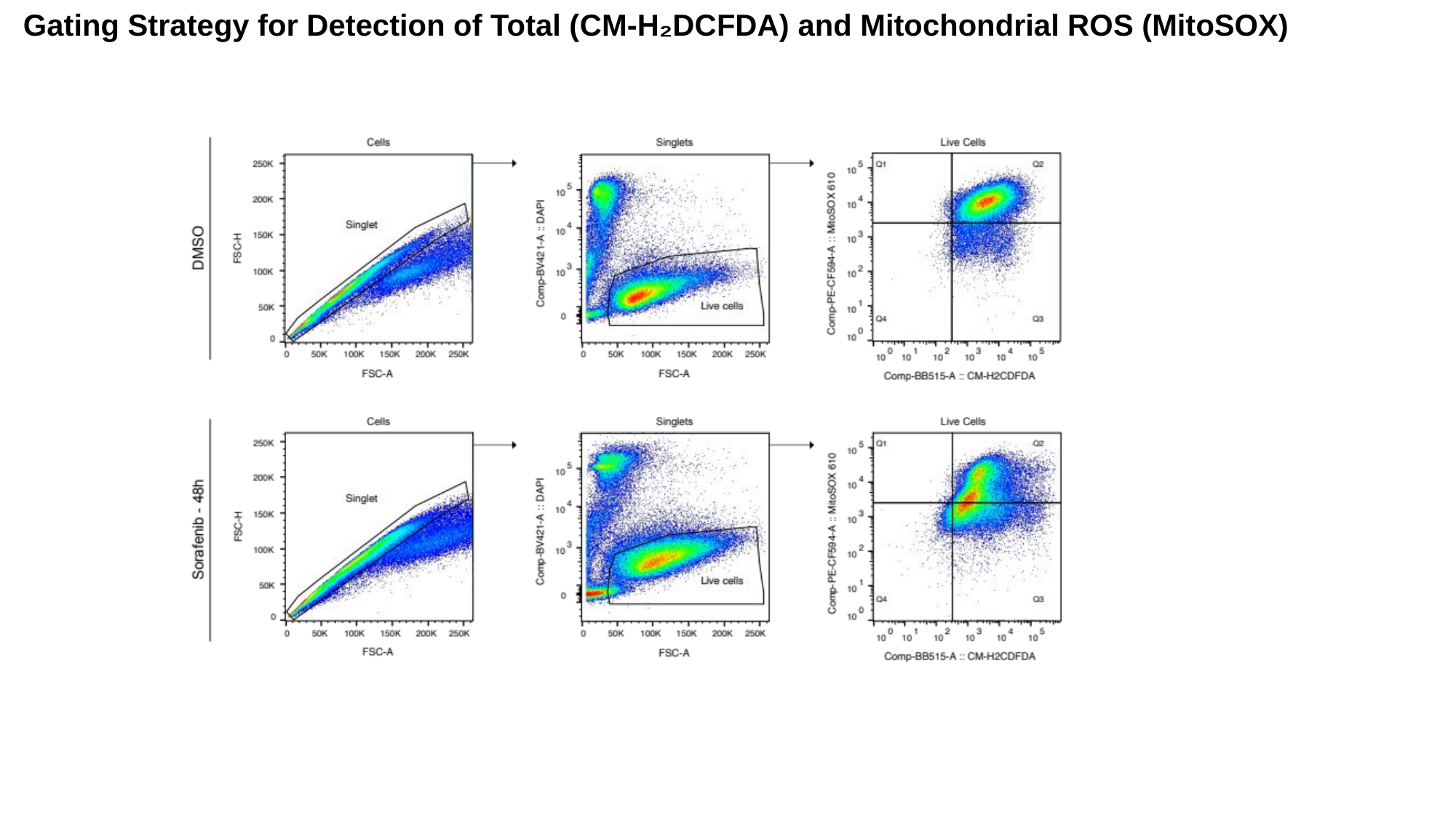

Gating Strategy for Detection of Total (CM-H₂DCFDA) and Mitochondrial ROS (MitoSOX)

## Slide 3
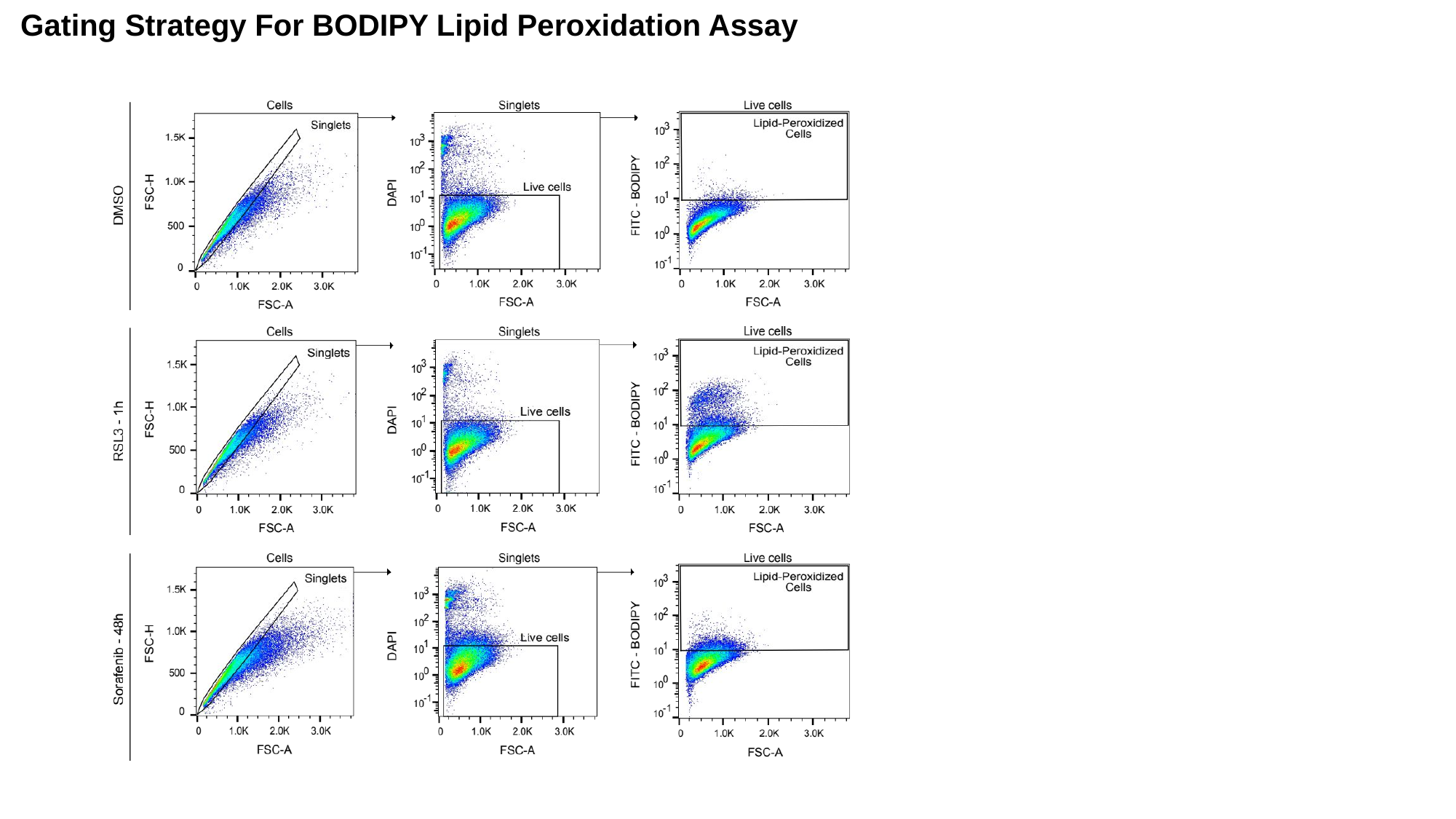

Gating Strategy For BODIPY Lipid Peroxidation Assay
